# HIV-1 envelope glycoprotein signatures that correlate with the development of cross-reactive neutralizing activity

**DOI:** 10.1186/1742-4690-10-102

**Published:** 2013-09-23

**Authors:** Tom L G M van den Kerkhof, K Anton Feenstra, Zelda Euler, Marit J van Gils, Linda W E Rijsdijk, Brigitte D Boeser-Nunnink, Jaap Heringa, Hanneke Schuitemaker, Rogier W Sanders

**Affiliations:** 1 Department of Experimental Immunology and Landsteiner Laboratory, Academic Medical Center, University of Amsterdam, 1105 AZ Amsterdam, the Netherlands; 2 Center for Integrative Bioinformatics VU (IBIVU) and Amsterdam Institute for Molecules, Medicine and Systems (AIMMS), VU University Amsterdam, 1081 HV Amsterdam, the Netherlands; 3 Netherlands Bioinformatics Center (NBIC), 6525 GA Nijmegen, the Netherlands; 4 Department of Medical Microbiology, Academic Medical Center, University of Amsterdam, 1105 AZ Amsterdam, the Netherlands; 5 Crucell Holland BV, 2333 CN Leiden, the Netherlands; 6 Department of Microbiology and Immunology, Weill Medical College, Cornell University, New York, NY 10065 USA

**Keywords:** Early HIV-1 variants, Envelope glycoprotein, Signatures, Cross-reactive neutralizing activity, Glycosylation, Broadly reactive neutralizing antibodies

## Abstract

**Background:**

Current HIV-1 envelope glycoprotein (Env) vaccines are unable to induce cross-reactive neutralizing antibodies. However, such antibodies are elicited in 10-30% of HIV-1 infected individuals, but it is unknown why these antibodies are induced in some individuals and not in others. We hypothesized that the Envs of early HIV-1 variants in individuals who develop cross-reactive neutralizing activity (CrNA) might have unique characteristics that support the induction of CrNA.

**Results:**

We retrospectively generated and analyzed *env* sequences of early HIV-1 clonal variants from 31 individuals with diverse levels of CrNA 2–4 years post-seroconversion. These sequences revealed a number of Env signatures that coincided with CrNA development. These included a statistically shorter variable region 1 and a lower probability of glycosylation as implied by a high ratio of NXS *versus* NXT glycosylation motifs. Furthermore, lower probability of glycosylation at position 332, which is involved in the epitopes of many broadly reactive neutralizing antibodies, was associated with the induction of CrNA. Finally, Sequence Harmony identified a number of amino acid changes associated with the development of CrNA. These residues mapped to various Env subdomains, but in particular to the first and fourth variable region as well as the underlying α2 helix of the third constant region.

**Conclusions:**

These findings imply that the development of CrNA might depend on specific characteristics of early Env. Env signatures that correlate with the induction of CrNA might be relevant for the design of effective HIV-1 vaccines.

## Background

The identification of the human immunodeficiency virus type-1 (HIV-1), as the causative agent of the acquired immunodeficiency syndrome (AIDS), initiated a long-term but as of yet unsuccessful search for an effective and safe HIV-1 vaccine. Ideally a vaccine should elicit both humoral and cellular immunity [1]. In particular the induction of cross-reactive neutralizing activity (CrNA) that is capable of neutralizing HIV-1 variants from different subtypes is appealing. Unfortunately, this has shown to be a difficult hurdle, and none of the vaccine candidates tested to date have been able to induce this type of humoral immunity [2-4]. The moderate efficacy against acquisition of infection in the RV144 vaccine trial [5], in which V1V2 IgG antibodies correlated inversely with the rate of infection [6], has lead to optimism in the HIV-1 vaccine field. In this trial, only very low titer, tier 1 neutralizing antibodies (NAbs) were detected and efficacy may improve with a vaccine that is capable of eliciting a cross-reactive neutralizing humoral immune response.

The HIV-1 envelope glycoprotein complex (Env) mediates entry into host cells and is the sole target for NAbs [7,8]. A functional Env consists of heterotrimers of three gp120 subunits, non-covalently linked to three gp41 molecules that anchor the Env spike in the viral membrane. Gp120 has five conserved (C1-C5) and five variable regions (V1-V5) [9,10]. The conserved regions form the core of the protein and are crucial for binding to the CD4-receptor on target cells [11-13]. The variable regions are highly diverse in sequence as a consequence of high replication rates, recombination, deletions, insertions and mutations [14]. Gp120 and gp41 contain 20–35 and 3–5 potential *N*-linked glycosylation sites (PNGS), respectively. *N*-linked glycans compose approximately half of the molecular mass of gp120 [15] and are required for correct protein folding, binding to lectin receptors on immune cells, as well as for immune evasion [16-20].

Within weeks to months after primary infection, HIV-1 Env specific antibodies appear [21] that are generally limited in their neutralizing activity and restricted to early autologous viruses [22-25]. The first antibodies are directed against gp41, predominantly to the immunodominant epitope, followed by non-NAbs against gp120, which in clade B-infected individuals often target the V3 region [26]. Subsequently, weakly neutralizing V3 antibodies capable of neutralizing heterologous tier 1 HIV-1 isolates appear [27] followed by NAbs with other epitope specificities from which the virus rapidly escapes through sequence changes in the variable loops and an increasing number of PNGS. Specifically, an increase in length and number of PNGS of the V1V2 region plays a role in HIV-1 resistance to NAbs [22,28-32].

Within 2–4 years post-seroconversion (post-SC), 10-30% of the HIV-1 infected individuals develop CrNA [33-40], which is directed against conserved epitopes such as the CD4-binding site (CD4BS), the membrane proximal region (MPER) in gp41 and glycan dependent epitopes that often include the glycans at positions 160 or 332 [41,42]. Over the years, a number of broadly reactive neutralizing monoclonal antibodies (bNAbs) have been isolated from HIV-infected individuals with CrNA. These antibodies show broad and potent activity against different HIV-1 subtypes. As HIV-1 can rapidly escape from autologous humoral immunity with high levels of CrNA [25,34], this immune response is not associated with prolonged asymptomatic survival of the individuals that make them [8,21,25,36,43,44]. Nonetheless, bNAbs can protect non-human primates against viral challenge in passive immunization studies [45-49], which supports the idea that an HIV-1 vaccine that elicits CrNA could be effective against HIV-1 acquisition in humans.

BNAbs delineate sites of vulnerability on the Env spike. These sites can be divided into four bNAb epitope clusters located at different positions on the Env trimer [50-53]. Three epitope clusters are located on gp120, and one on gp41. The first epitope cluster is the CD4BS, which is the epitope for multiple bNAbs such as b12, VRC01, VRC-PG04, NIH45–46 and 3BNC117 [54-58]. The more recently discovered PGT121-130 and PGT135 bNAbs, as well as 2G12, target the outer domain on gp120 and are N332 glycan dependent [52,59-63]. BNAbs PG9, PG16, PGT145 and CH01-CH04 target conserved epitopes within V1V2 that are expressed on trimeric Env [52,53,64-66]. Preferential binding of PG9 to the quaternary structure of trimeric Env could be explained by its binding to an epitope at the apex of the trimer that constitutes elements from two protomers [67]. PG9, PG16 and PGT145 neutralization is dependent on the presence of glycans, especially at position N160 [64,68]. One epitope cluster is located in the MPER of gp41, which contains the binding sites for 2F5, 4E10 and 10E8 [69-72].

The extensive glycosylation of gp120, amounting to ~50% of its molecular weight, was long thought to contribute to an immunologically “silent face” and serve as a “glycan shield” [8,73]. The recent identification of many glycan-dependent bNAbs shows that the silent face may not be so immunologically silent after all, and that this shield can be penetrated and/or used by bNAbs. The epitope cluster on gp120 which is targeted by the bNAbs PGT121-130, PGT135 and 2G12 [60-62,67,74], requires a specific glycan at position 332, although there is a difference in how the different bNAbs approach this glycan. In addition, neutralization activity in serum of infected humans and macaques, which developed CrNA, is dependent on recognition of the epitope involving this glycan at position 332 [36,75,76]. Interestingly, Moore *et al*. recently showed that the 332 glycan was absent on the Env of early viruses from two clade C infected individuals but emerged as a means of escape from autologous neutralizing responses, thereby creating 332 glycan-dependent bNAb epitopes [77]. For these reasons it seems that the glycosylation at position 332 plays a substantial role in the development of CrNA.

Although the existence of bNAbs in natural infection is testimony that the native Env spike can elicit bNAbs, it remains unknown why this occurs in only 10-30% of HIV-1 infected individuals. Here, we hypothesized that specific properties of early Envs could contribute to the development of CrNA. Information on such properties would obviously be valuable for vaccine design aimed at generating similar CrNA [78]. To test our hypothesis, we retrospectively examined *env* sequences from early HIV-1 clonal variants in clade B infected individuals that developed diverse levels of CrNA later on during infection. We found that CrNA development correlated with early HIV-1 variants with shorter V1 regions, lower probability of glycosylation, and specific amino acid usage. These properties might open up avenues for vaccine design.

## Results

### Short V1 sequences correlate with the development of cross-reactive neutralizing activity

**Figure 1:**
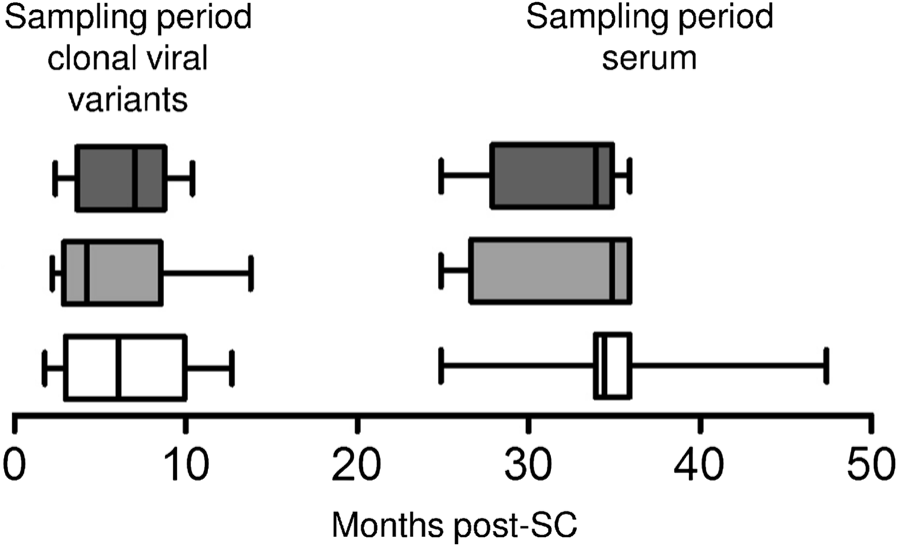
**Sampling of viruses and sera.** Time bars showing the period in which viruses were isolated and the period in which sera were obtained for assessment of neutralizing activity. The white, grey and dark grey bars represent the individuals with non-CrNA (n=9), intermediate CrNA (n=10) and CrNA (n=12), respectively. The x-axis represents the months post-SC. The box-plots represent the sampling periods with minimum and maximum time points indicated by whiskers. The median time points of virus and sera sampling for the 31 individuals are indicated by solid vertical lines in the boxes.

**Figure 2:**
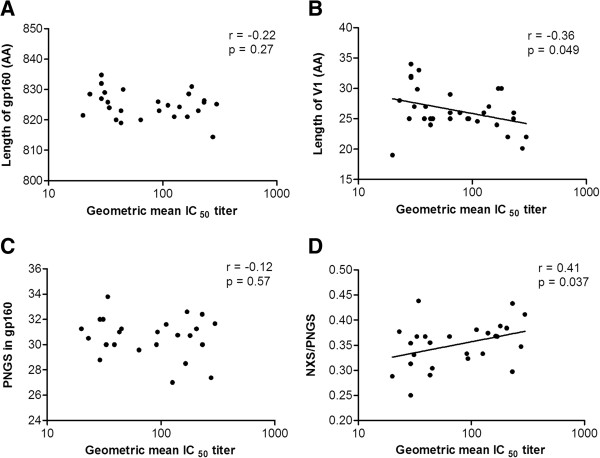
**Short V1 sequences and lower probability of overall glycosylation correlate with the development of cross**-**reactive neutralizing activity.** Scatter plots of individual’s geometric mean IC_50_ titer across the 6 virus panel (x-axis) versus sequence characteristics of the gp160s from clonal virus isolates on the y-axis. **(A)** length of gp160 in amino acids (AA); **(B)** length of variable region 1 (V1) in amino acids (AA); **(C)** total number of PNGS in gp160; **(D)** number of NXS motifs relative to the total number of PNGS.

To study Env determinants that may influence the induction of CrNA, we retrospectively generated *env* sequences from early HIV-1 variants in 31 individuals who at 2–4 years post-SC had diverse levels of CrNA in their serum (Figure 1) [35,79]. We chose this experimental setup because contemporaneous viruses usually already have escaped from CrNA [25,34] and early viruses are proposed to be a major determinant for the induction of CrNA [80]. The individuals were matched for time between SC and CrNA measurement, time between SC and clonal virus isolation, CD4^+^ T cells/μl blood at set-point and viral load at set-point (Additional file 1: Figure S1). Data on HIV-1 neutralizing activity in serum were available from previous studies (n = 292) [35,40,79]. In short; sera were tested by Monogram Biosciences [81] for cross-reactive neutralizing activity in a pseudovirus assay involving six tier two viruses with Envs from primary isolates of HIV-1 subtypes A (94UG103), B (92BR020 and JRCSF), C (93IN905 and MGRM-C-026) and CRF01_AE (92TH021). This six viral panel covered 93% of the variation in neutralization of a larger pseudovirus panel (n = 15) [39]. It has been shown that classification of CrNA in sera, as determined on this six virus panel, was highly correlated with CrNA determination on a larger 23 virus panel [35]. The geometric mean IC_50_ titers in the sera of the selected individuals against a panel of 6 HIV-1 variants varied from 20 to 297; with an average of 98 (see Table 1 for details). Phylogenetic analysis of all sequences, using either neighbour-joining or maximum-likelihood methods, revealed clustering of sequences per individual, excluding contamination, but, clustering of HIV-1 Env sequences from individuals with similar geometric mean IC_50_ titer was not observed (Additional file 2: Figure S2). We observed that the geometric mean IC_50_ titer in serum correlated weakly with the mean length of V1 ((Figure 2B, r = −0.36; p = 0.049) but not with overall mean gp160 length (Figure 2A), nor with the total length of either the variable or the conserved regions (data not shown, r = −0.21; p = 0.25 and r = −0.052; p = 0.80, respectively).

**Table 1:** **Individual**’**s data summary**

**Individual**	**Geometric mean IC**_**50 **_**titer**^**a**^	**Number of viruses neutralized**^**b**^	**Date of SC**^**c**^	**Months from SC to CVI**^**d**^	**CD4 count at SP**^**e**^	**Viral load (log) at SP**^**f**^	**Env sequences obtained**^**g**^	**Neutralization assay performed**^**h**^	**SH analysis performed**^**i**^
ACH19829	297	6	26-jan-86	10.5	440	3.9	gp160	+	+
ACH18969	275	5	29-Oct-87	1.8	520	4.3	gp160	+	+
ACH19308	231	5	14-jul-91	3.3	380	4.4	gp160	+	+
ACH18814	230	6	27-jul-87	3.2	500	5.1	gp160	+	+
ACH18818	205	4	26-jan-86	8.2	510	5.4	gp160	+	+
ACH19885	180	5	28-dec-94	2.9	580	3.3	gp160	+	+
ACH11668	169	5	28-jun-86	7.9	380	4.5	gp160	+	+
ACH19463	164	5	14-jul-86	2.1	350	4.8	gp160	+	+
ACH19507	140	6	23-apr-88	7.9	400	5.0	gp160	+	+
ACH19474	126	5	27-jul-87	12.7	410	3.0	gp160	+	+
ACH19999	111	4	26-aug-85	4.3	1060	4.6	gp160	+	+
ACH19793	93	4	28-jun-86	12.3	1000	4.5	gp160	+	+
ACH19588	91	4	20-nov-85	13.8	600	5.3	gp160	-	-
ACH19566	78	5	11-Mar-85	13.2	610	3.5	C1-V5	-	-
ACH19768	64	5	6-apr-86	2.2	370	3.7	gp160	-	-
ACH18860	64	2	25-aug-86	2.6	550	4.9	C1-V5	-	-
ACH18839	64	3	17-Oct-86	3.3	330	3.8	C1-V5	-	-
ACH19542	45	3	23-jun-85	4.5	310	3.9	gp160	-	-
ACH18766	43	1	15-jul-88	5.1	760	4.3	gp160	-	-
ACH19861	43	3	24-feb-89	3.0	470	4.3	gp160	+	+
ACH19453	39	3	23-dec-85	7.0	810	3.0	gp160	-	-
ACH18829	38	3	2-feb-87	4.0	770	2.6	C1-V5	-	-
ACH19792	34	2	8-nov-85	6.9	450	4.9	gp160	+	+
ACH19961	31	0	14-sep-87	2.4	530	4.5	gp160	+	+
ACH19329	31	1	28-dec-94	4.3	780	3.9	gp160	+	+
ACH19974	29	2	26-jan-86	8.8	550	3.5	gp160	+	+
ACH19489	29	0	30-sep-85	7.4	550	3.1	gp160	+	+
ACH18887	29	1	7-dec-89	8.8	670	4.6	gp160	+	+
ACH19342	28	2	14-jul-86	3.0	700	3.9	C1-V5	-	-
ACH19576	23	1	2-feb-88	7.0	500	4.5	gp160	+	+
ACH18880	20	0	28-jun-86	10.4	860	4.8	gp160	+	+

^a^Neutralization titers are expressed as the geometric mean of the reciprocal plasma dilution that inhibited virus infection by 50% for the panel of 6 heterologous viruses.

^b^The number of viruses neutralized, at least 3-fold higher than the IC_50_ of the same serum with MLV, from the panel of 6 heterologous viruses from different subtypes.

^c^Date of seroconversion (SC).

^d^Months post SC to time of clonal virus isolation (CVI).

^e^CD4^+^ T-cell (CD4^+^) count at setpoint (SP).

^f^Log viral load at setpoint (SP).

^**g**^Env fragment sequenced.

^h^Individuals from which viruses were used in the neutralization sensitivity assay with MAbs and polyclonal HIVIg pools are indicated with +.

^I^Individuals from which viral sequences were used in the Sequence Harmony model are indicated with +.

### Lower probability of overall glycosylation correlates with the induction of cross-reactive neutralizing activity

We did not observe a correlation between the geometric mean IC_50_ titer in serum, and the mean total number of PNGS in gp160 (Figure 2C) or the mean number of PNGS in the individuals variable or constant regions (data not shown). It has been shown that NXT motifs have a two to three times higher probability of becoming glycosylated than NXS motifs [82,83]. The total number of PNGS may therefore not entirely reflect the actual extent of glycosylation of a given Env molecule. Interestingly, the mean number of NXS acceptor motifs relative to the mean total number of PNGS correlated positively with the geometric mean IC_50_ titer in serum (Figure 2D, r = 0.41; p = 0.037), while the opposite was true for the mean number of NXT acceptor motifs relative to the mean total number of PNGS. Thus, a higher relative number of NXS over NXT motifs, i.e. less probability of glycosylation was associated with the development of CrNA. However, we note that we cannot make statements on the actual glycosylation of individual NXS and NXT motifs.

### Lower probability of glycosylation at position 332 correlates with the induction of cross-reactive neutralizing activity

**Figure 3:**
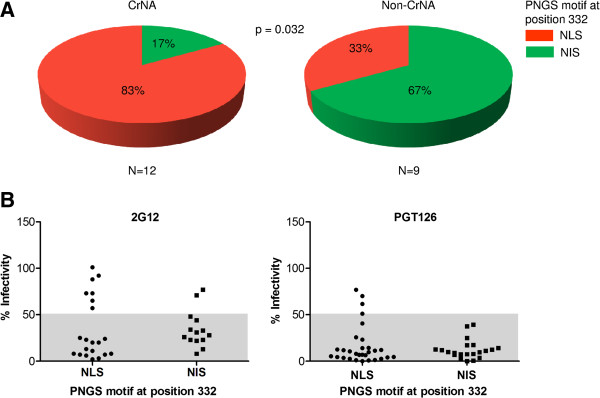
**Lower probability of glycosylation at position 332 correlates with the induction of cross-reactive neutralizing activity. (A)** Pie charts representing the distribution of NLS and NIS PNGS motifs at position 332 between 12 individuals who developed and 9 individuals who did not develop CrNA. Red and green represent NLS and NIS motifs, respectively. **(B)** Residual virus infectivity in the presence of high concentrations of 2G12 (25 μg/ml), excluding viruses lacking one or more of the essential 2G12 glycans 295, 332, 386 or 392 [61,62,85], for virus clones that have NLS or NIS motif. **(C)** Residual virus infectivity in the presence of high concentrations of PGT126 (5 μg/ml), for virus clones that have NLS or NIS motifs. Values in the gray area are considered sensitive, while values in the white area are considered resistant.

We noted that the glycosylation motif at position 332, which is important for a number of bNAb epitopes [52,59-62,74], is always of the NXS type, and may thus not always be occupied by a glycan. In particular when the acceptor motif is NXS, the amino acid at position X is also important for determining the probability of glycosylation [84]. In all our sequences, the second position was occupied by either a leucine or an isoleucine. We found that individuals who developed CrNA had significantly more often an NLS motif at position 332, while individuals who did not develop CrNA had more frequently an NIS motif (Figure 3A, p = 0.032). Studies with rabies virus glycoprotein showed that an NLS motif has a two times lower probability of becoming glycosylated compared to an NIS motif [84], which might imply that a lower probability of *N*-glycan attachment to N332 is associated with the development of CrNA. There is no straightforward way to directly assess glycan occupancy of position 332 on virus isolates, but we can study this indirectly by investigating the neutralization sensitivity to bNAbs that are dependent on the presence of a glycan at position 332, such as 2G12 and PGT126. Thus, we tested the sensitivity of the viruses from our infected individuals against 2G12 and PGT126, and divided them into two groups, for the presence of NLS or NIS at position 332. For 2G12 we excluded viruses that lacked one or more of the essential 2G12 glycans 295, 332, 386 or 392 [61,62,85] (i.e. the selected viruses should in theory all be sensitive to 2G12). We defined resistance as >50% infectivity at the highest concentration of bNAb (25 μg/ml and 5 μg/ml for 2G12 and PGT126, respectively; Figure 3B&C). We found that 7 out of 21 viruses (33%) with an NLS motif were resistant to 2G12, while only 2 of 14 viruses (14%) with an NIS motif were resistant (Figure 3B). Furthermore, we found that 4 out of 30 viruses (13%) with an NLS motif were resistant to PGT126, while none of the viruses with an NIS motif were resistant (Figure 3C). All NLS containing viruses that were resistant to PGT126 were also resistant to 2G12. Although these differences did not reach statistical significance they are consistent with a lower probability of glycosylation of NLS motifs, of which the presence is associated with the development with CrNA.

### Sequence Harmony identifies specific amino acids that correlate with the induction of cross-reactive neutralizing activity

The Sequence Harmony (SH) method ([86] and http://www.ibi.vu.nl/programs/seqharmwww) was used to identify amino acid differences in Env between individuals who developed CrNA and who did not (Table 2). Low SH-scores indicate positions where the amino acid composition is different between the two groups; a score of 0 indicates that the amino acids at a given position are completely different between both groups, while the maximum score of 1 indicates that the amino acid compositions are indistinguishable. In addition, an empirical Z-score is calculated, reflecting the significance of the SH score obtained based on 100 random shuffling events of the sequences between the two groups. The SH analysis yielded 39 sites with a SH score below the defined cut-off values, indicating differences in amino-acids present at these sites between the CrNA and non-CrNA groups, all with high (negative) Z-scores indicating high significance (Table 2). A phylogenetic analysis showed the absence of sequence group clustering (Additional file 2: Figure S2), excluding spurious findings based on phylogeny. Individual sequences were weighted in the calculation of the SH-scores, such that each individual had equal weight (see Methods for details). For comparison, we repeated the analysis with a single consensus sequence per individual. This yielded essentially identical scores (the correlation between SH-scores and Z-scores is both r = 0.98, and p < 0.0001), but less detailed information on the specific amino acid changes (Additional file 3: Table S1 and Additional file 4: Figure S3). For the variable domains we used a cutoff of SH <0.7 and for the conserved domains a more relaxed cutoff of SH <0.85. This extended (higher) cut-off is necessary to find small differences within the conserved regions that have an overall higher sequence similarity. 22 sites were located in the variable regions, including 1 distinct site with the maximal score of 0.0 located in the V1 region which also contains 10 other sites scoring below 0.7 (z-scores ranging from −8 to −69). In the multiple sequence alignment, 6 insertions occur within the V1 region between positions 140 and 141 and are in agreement with the observed longer V1 region in individuals who did not develop CrNA (Figure 2B). One site is located in the V3 region (position 322), 8 sites in V4 (396, 399, 403–407 and 412) and 2 sites in V5 (461 and 464) all with z-scores ranging from −9 to −35. As conserved regions show inherently higher sequence conservation than the variable regions, a less strict cut-off of SH <0.85 was used. This resulted in the selection of 17 sites: 6 in the C1 region (32, 33, 85, 87, 97 and 130), 3 in C2 (268, 271 and 275), 6 in C3 (333, 336, 337, 343, 346 and 347), and 1 site in C4 (S440) (z-scores ranging from −3 to −16). The change at position 333 in C3 corresponds to the second position of the N332 glycosylation motif (see above). One additional relevant site was identified in the ectodomain of gp41 (621).

**Table 2:** Sequence Harmony results

				**Consensus string**^**d**^		
**Region**	**Amino acid position**^**a**^	**SH-scores**^**b**^	**Z-scores**^**c**^	**High CrNA**^**e**^	**Low CrNA**^**f**^	**Cluster**^**g**^
C1	E32	0.825	−5.5	Egd	Edkng	1
C1	K33	0.765	−6.7	KQn_e	QNdek	1
C1	V85	0.792	−4.8	Vkaei	Ver	2
C1	V87	0.843	−4.9	Ekg	Eadg	2
C1	K97	0.840	−5.1	Kint	Knr	3
C1	K130	0.843	−8.5	Ne	Nd	4
V1	L134	0.620	−11.4	Lf	VL_aim	5
V1	K135	0.346	−17.0	RKeqg	-hknwg	5
V1	N136	0.698	−12.9	Nt	N-t	5
V1	T138	0.585	−14.4	Tn_	-gast	6
V1	N139	0.650	−12.5	N_t	-nist	6
V1	T140	0.531	−12.0	Tiks_n	-alns	6
V1	.141	0.522	−13.2	T-spgn	SR-egk	6
V1	.141	0.368	−38.3	-tn	N	6
V1	.141	0.223	−24.4	-sktni	TAeiv	6
V1	.141	0.000	−69.1	-h	T	6
V1	.141	0.000	−45.6	-	Nads	6
V1	.141	0.664	−16.1	-ts	Tnsa_	6
V1	N141	0.418	−21.7	NTs	N-tip	6
V1	S142	0.697	−12.1	Ns	Nt_i	6
V1	.145	0.518	−17.3	W-g	Snw_	
V1	.146	0.507	−15.8	-m	LI-vg	
V1	K151	0.696	−8.3	E-GRtak	GK-qte	6
C2	E268	0.814	−4.6	Ekqg	Edgr	7
C2	V271	0.719	−15.3	Vi	Va	8
C2	V275	0.587	−9.0	Eknvad	Esnqk	3
V3	K322	0.611	−17.4	DE	Eaq	9
C3	I333	0.804	−15.5	Li	Il	10
C3	A336	0.684	−9.1	TAgvel	Aekv	10
C3	K337	0.686	−10.1	EQKntd	Knq	10
C3	K343	0.667	−8.4	Kqsg	EQKgnh	11
C3	A346	0.809	−15.9	AV	V	11
C3	S347	0.837	−3.1	TIndesk	NKEITr	11
V4	F396	0.578	−16.4	NT-wf	-NG	11
V4	T399	0.604	−10.9	-vt	RT-ans	11
V4	.403	0.686	−16.1	-t	N-s	
V4	.404	0.537	−11.7	-ksr	KT-epn	
V4	S405	0.661	−15.4	-Sdl	-evs	10
V4	N406	0.377	−34.9	N_t	-n	10
V4	N407	0.277	−18.9	N_srk	-thn	10
V4	.412	0.574	−13.5	-s	N-wde	
V4	.412	0.689	−11.6	-	-snt	
C4	S440	0.729	−6.8	Raks	KReg	12
V5	S461	0.650	−14.0	DE-sn	-n	
V5	.464	0.692	−9.4	NEK-	-rk	13
V5	.464	0.557	−15.2	-nda	-Nti	13
gp41	Q621	0.540	−9.4	Kestardq	EMYQNK	

^a^Amino acid numbering based on the HXB2 reference sequence.

^b^Sequence Harmony scores (SH-scores): SH-scores below a cut-off of <0.7 for variable regions and a cut-off of <0.85 for conserved regions are shown.

^c^The z-scores display the accuracy of a given result; the more negative the z-score the less likely it is that the results was found by chance.

^d^The ’consensus string’ shows the residues found in each group on that specific site ordered by frequency were a lower case letter is present less than half the amount of the most abundant residue (upper case).

^e^Individuals who developed CrNA.

^f^Individuals who did not develop CrNA.

^g^Numbers represent the subclusters as defined in Additional file 5: Table S2.

### Structural mapping of the Sequence Harmony results reveals clustering of amino acids that are associated with the induction of CrNA

**Figure 4:**
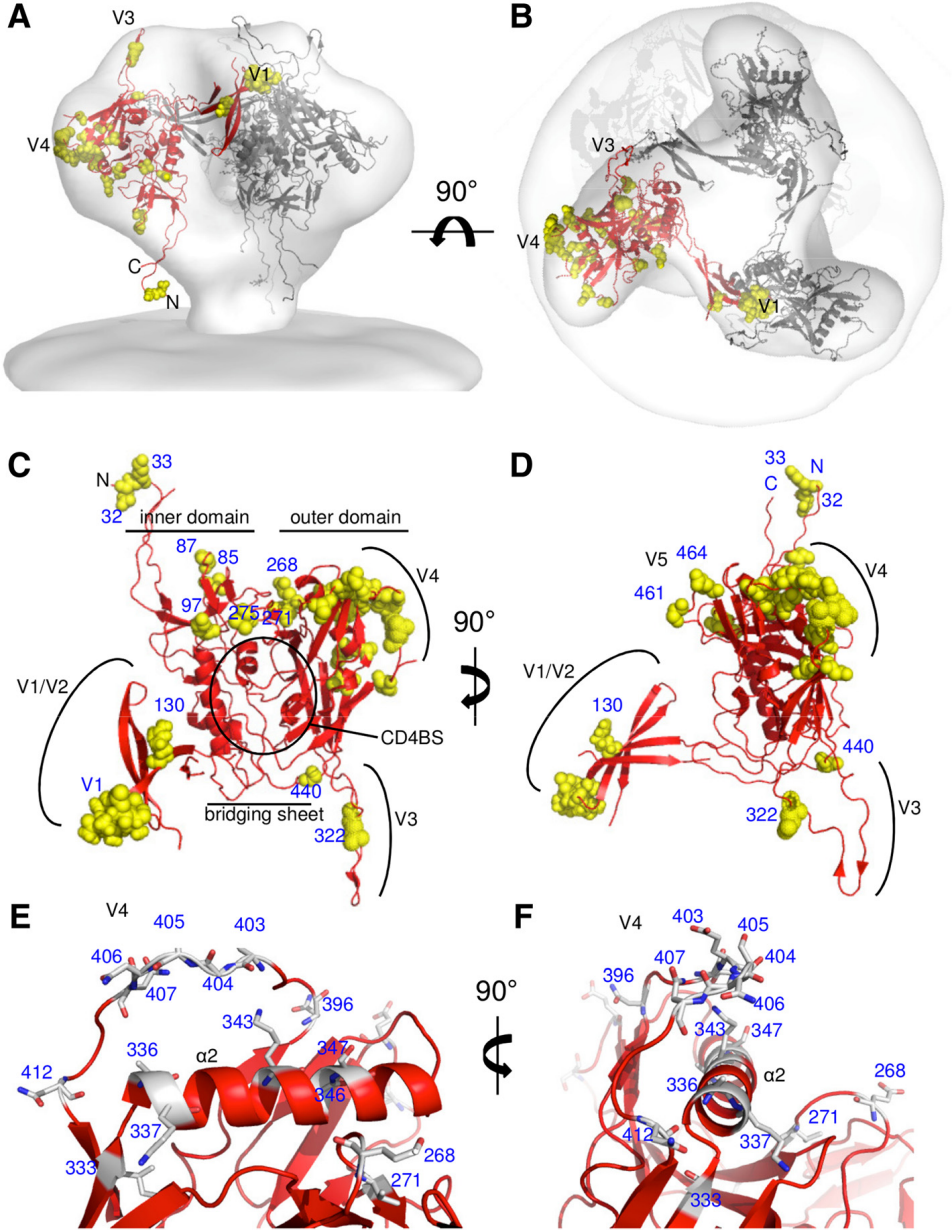
**Three dimensional clustering of amino acid positions that differ between individuals that induce CrNA and those that do not.** Side **(A)** and top **(B)** views of the Env spike. The structures of three gp120 protomers, including the entire gp120 sequence and modeled as described in the Methods section, were fitted into the cry-EM density of the virus-associated Env spike [87]. A backbone trace of one protomer is colored in red and the positions revealed by SH are indicated in yellow space filling in the same protomer. **(C**, **D)**. Model of gp120 with the same color-code as in A and B. Various Env subdomains are indicated in black font. Residues identified by SH are labeled in blue. The views are from the approach of CD4 **(C)** or rotated by 90° over the y-axis **(D)**. The viral membrane would be at the top and the target membrane at the bottom. **(E**, **F)**. Details of the V4 domain and its association with the α2 helix of the C3 domain. The residues identified by SH are indicated in sticks.

To better understand the impact of the results gained with SH, we mapped the positions that differed significantly on the three-dimensional structure of Env (Figure 4). A number of gp120 crystal structures were combined, as described in detail in the Methods section, to generate a structure of the Env spike that contains all gp120 residues (Figure 4A and B). Gp41 was not included as it contains only one position that was identified by SH (at position 621). Although this model may not be an accurate representation of the Env spike with correct positions and conformations of the variable domains, it is useful to visualize the residues identified by the SH analysis. The carbon trace is indicated in red, while the residues selected by the SH analysis are shown in yellow space filling. Mapping of the residues identified by SH on the 3D structure of Env reveals that the residues that differ between the Envs, which are derived from individuals that did develop and those that did not develop CrNA, cluster together in specific domains. First, a number of residues cluster in the V1 (Figure 4C and D). Second, a large cluster includes a major portion of the V4 as well as part of the underlying C3. Looking into these positions in more detail it is evident that a number of C3-residues located on the side of the α2 helix that faces the V4 are different between the two sequence groups and those residues that differ in the V4 are all close to the α2 helix (Figure 4E and F). A third cluster of residues can be observed in the gp41-interactive domain, including residues in C1 and C2, although these residues are more scattered throughout the domain, compared to those in the first two clusters. It is also obvious that some large regions in the protein do not contain any changes, most notably the CD4BS and the V2. Only one change is present in the bridging sheet, and also only one in the V3. The residues identified by SH can be further subdivided into thirteen subclusters based on their proximity to each other (<9 Å; Additional file 5: Table S2).

### The induction of cross-reactive neutralizing activity does not correlate with sensitivity to bNAbs

**Figure 5:**
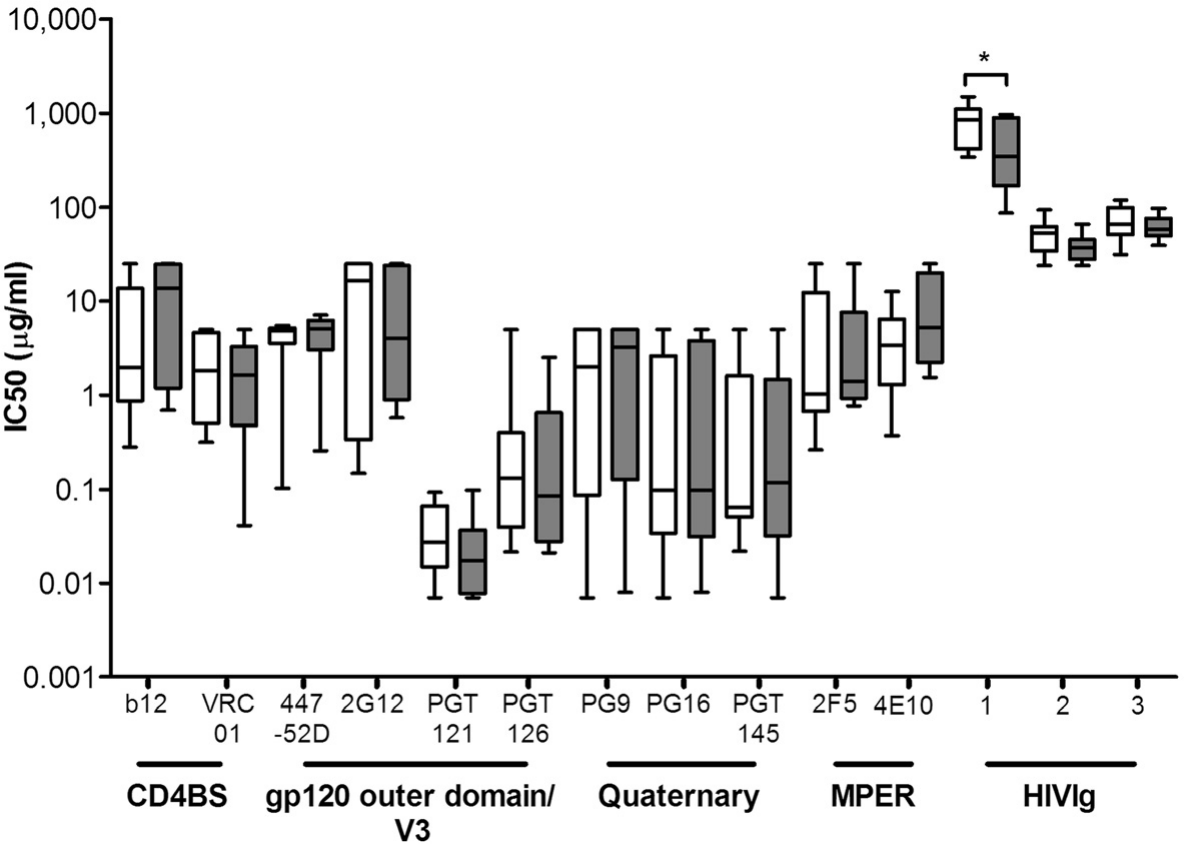
**Sensitivity to bNAbs does not correlate with the induction of cross-reactive neutralizing activity.** Sensitivity to neutralization by b12, VRC01, 447-52D, 2G12, PGT121, PGT126, PG9, PG16, PGT145, 2F5 and 4E10 and polyclonal HIVIg (three different sera pools) of viral variants obtained from 12 individuals who developed CrNA (grey bars) and 9 individuals who did not develop CrNA (white bars). Median IC_50_ values per individual per bNAb or HIVIg as determined by linear regression are used and differences were considered statistically significant when P values were ≤ 0.05, represented by an asterix. The bNAbs are ordered from left to right according to their epitope cluster.

We tested whether the early HIV-1 variants from individuals who developed CrNA were more sensitive to neutralization by bNAbs covering all known epitope clusters (gp120 outer domain: 447-52D, 2G12, PGT121 and PGT126; CD4BS: b12 and VRC01; quaternary epitopes: PG9, PG16 and PGT145; MPER: 4E10 and 2F5). We did not observe that the viral variants of individuals who developed CrNA were more sensitive to neutralization by different bNAbs compared to viruses from individuals who did not develop CrNA (Figure 5). We also analyzed the neutralization data for each bNAb per individual and we observed that the neutralization pattern was not dependent on the level of CrNA (Additional file 6: Table S3). We next tested the sensitivity of these viruses to three different polyclonal HIVIg pools in which multiple epitope specificities should be present. Interestingly, early HIV-1 variants of individuals who developed CrNA showed a trend towards being more sensitive to neutralization by polyclonal HIVIg compared to early viruses from individuals who did not develop CrNA (Figure 5), which could be consistent with them displaying a more open Env structure. However, this was only statistically significant for one of the three HIVIg pools (p=0.037).

## Discussion

In the Amsterdam Cohort Studies on HIV infection and AIDS (ACS) of men who have sex with men (MSM) infected with HIV-1 subtype B, the prevalence of CrNA at 2–4 years post-SC is around 33% [40,79]. This roughly corresponds to what has been found in other cohorts [33,37-39]. In this study we aimed to identify differences in the properties of early Env proteins in 31 HIV-1 infected individuals in regard to the development of CrNA. We observed that a short V1 region and a lower probability of *N*-linked glycosylation at PNGS correlated with the development of CrNA later in infection. More specifically, a lower probability of glycosylation at position 332 was associated with the development of CrNA. However, we note that the studies on the glycosylation efficiency of NXS/T motifs have been performed with a non-HIV protein [84] and the probability of glycosylation is likely to be context dependent. We can, therefore, not make definitive statements on the actual glycosylation of individual viruses or groups. Although some of the observed associations were marginally significant, and require confirmation in independent studies, they are in line with the hypothesis that HIV-1 Envs from individuals who develop CrNA have a more open structure, which allows for increased accessibility of bNAb epitopes, and possibly more efficient induction of bNAbs. To confirm this hypothesis we conducted a neutralization assay with early HIV-1 clonal variants from only the individuals who did or did not develop CrNA and found that early viruses of individuals who developed CrNA were indeed more sensitive to neutralization by one polyclonal HIVIg sample (but not by two other HIVIg samples), although we were not able to map this to specific bNAb epitopes. We next used SH for a more detailed analysis of amino acid differences at specific positions in Env and their role in the induction of CrNA. This resulted in the identification of 39 Env residues which differed significantly between the two groups, based on defined SH-scores and z-scores. These residues showed a remarkable clustering in V1, C3-V4, and somewhat more diffusely in C1-C2. In addition, a few individual changes were observed: one in V3, one in C4, three in the V5, and one in the gp41 ectodomain. Strikingly, we did not find any residues in the V2 domain, in the CD4BS, or in the triple layered structure that translates CD4 induced conformational changes to gp41 [88]. We note that the differences we found might be specific for individuals who are infected with HIV-1 subtype B, and should be reproduced in other non-subtype B cohorts.

Our data revealed an inverse correlation between V1-length and the development of CrNA. Two previous studies have looked for Env signatures associated with the presence of CrNA, but these studies used contemporaneous samples for Env analysis and assessment of neutralization breadth and potency, and it is not likely that the contemporaneous Envs represent the Envs that induced the CrNA [89,90]. Nevertheless, in the first study, involving clade C infected individuals, short V1V2 domains correlated with the presence of CrNA, but the V1 and V2 were not studied separately [90]. In the second study, involving individuals infected with different clades, short V2 and short V5 regions correlated with the presence of CrNA in the sera [89]. Again, the caveat in these studies is that the Env sequences and sera were derived from the same time point during chronic infection; therefore it cannot be excluded that the Env characteristics observed were a consequence rather than a cause of the CrNA in serum, due to viral escape from the CrNA. In contrast, we studied the Envs of early viruses (median 5 months post-SC), before the presence of CrNA, and related the characteristics to the development of CrNA later in infection. It is arguably more likely that the observed Env signatures we identified indeed contributed to the shaping of the CrNA. Although other choices could have been made in the timing of our sampling, Moore et al. showed that transmitted/founder viruses raise autologous neutralization, which triggers the evolution of escape variants around month 6 (roughly around the same time as our sampling of env sequences), which in turn induce CrNA [77].

The SH-analysis confirmed a role for V1, but in addition to the deletions/insertions (at positions 141, 145 and 146) identified a number of other amino acid changes in V1 that were associated with the development of CrNA (at positions 134–140, 142 and 151). It has long been known that V1 and V2 are not absolutely required for function [10,91], but that they play an important role in resistance to antibody neutralization [28,31,92-96]. On a population level the continuous neutralizing antibody-driven evolution of V1V2 during the pandemic appears to have resulted in the elongation and increased glycosylation of the V1V2 domains [97]. It may therefore not come as a surprise that short V1 domains, with a specific amino acid composition, might affect the induction of CrNA. We note, however, that we found no residues in V2 that differed between the two groups. This could suggest that V1 length and composition are more important determining factors in the induction of CrNA than V2 length and composition, at least in our study population.

Previous studies indicated that the Envs from transmitted/founder HIV-1 variants contain fewer PNGS compared to Envs from viruses in chronic infection [98]. The increase in number of PNGS coincides with a decreasing sensitivity to autologous neutralization and is proposed to be driven by the pressure of neutralizing antibodies [8,22,29,30,32,99]. Furthermore, a reduced prevalence of CrNA in recently infected individuals compared to historic serum samples was associated with an increased number of PNGS in early Env sequences [97], suggesting that an increase in Env glycosylation over the course of the epidemic results in decreased induction of CrNA. Thus, an increased number of PNGS and a higher level of *N*-linked glycosylation could interfere with the induction of bNAbs, as these glycans may interfere with the binding of the B cell receptor to protein epitopes on Env. We did not observe a correlation with the absolute number of PNGS and the later presence of CrNA. However, we did observe that a lower probability of glycosylation at the PNGS in early HIV-1 variants was associated with CrNA presence later in infection. In previous Env sequence analyses, NXS and NXT PNGS motifs have always been treated equally, but NXS motifs are two to three times less likely to be glycosylated than NXT sequons [82,83,100]. Furthermore, when an NXS motif is present, the amino acid at the second position (the X) becomes highly relevant to determining the probability of glycosylation [84].

Zooming in on the NXS motif at position 332, the glycan which is critical for the binding of many known bNAbs [52,59-62,74] and which was associated with neutralization escape from early strain-specific antibodies and induction of CrNA [77], we observed a correlation between the amino acid at the second position and the presence of CrNA in serum later in infection. Thus, individuals who developed CrNA had significantly more often an NLS motif at position 332 compared to the individuals who did not develop CrNA who usually had an NIS motif. An NLS motif is two to three times less likely to be glycosylated compared to an NIS motif [84], implying that a lower chance of glycosylation at position 332 is associated with the development of CrNA. The probability of glycosylation and how HIV-1 might use this is unchartered territory and should be considered in other studies on the role of Env glycosylation in the co-evolution of the virus and the human immune system.

The association of a lower probability of glycosylation at position 332 with the induction of CrNA is counterintuitive when taking into account that many known bNAbs target this glycan [52,59-62,74]. There are two possible explanations for this paradox. First, this glycan is not often targeted in our study population and its absence increases the accessibility of other bNAb epitopes. Second, it is not the presence of this glycan per se that facilitates the induction of bNAbs to this site, but the emergence of this site during infection, possibly by means of escape from Abs that targeted the surrounding region in the absence of the glycan. This later scenario was indeed observed in individuals infected with clade C Env [77]. The situation in our cohort was slightly different because the NXS motif at position 332 was present in all sequences, only the 2^nd^ position changed over time. Furthermore the absence of the PNGS at position 332 in transmitted subtype C was accompanied by the presence of a PNGS at position 334 [77], while we never observed a PNGS motif at position 334 in our sequences. The impact of the PNGS at one of these two positions, and its relation with subtype specific transmission and antibody escape and induction, requires further study in different cohorts.

The SH analysis identified multiple amino acid positions in the C3 and V4 region that differed significantly between individuals that did or did not develop CrNA. Inspection of a quaternary model of the Env spike showed that many of these positions form two subclusters on the α2-helix in the C3 region (HXB2 numbering 335–352) and the V4 loop (Figure 4). One cluster contains 6 residues: 333 (which may modulate glycosylation at position 332; see above), 336 and 337 from C3, and 405–407 from V4. The second subcluster involves 5 residues: 343, 346 and 347 from C3, and 396 and 399 from V4. The C3 α2-helix interacts intimately with V4 and several of the differences found between individuals that did or did not develop CrNA might impact the interaction between the two subdomains. For example, it has been reported that interactions between residues 335 and 412, and 337 and 412 play an important role in the interaction of these two regions through electrostatic interactions [101]. In our SH analyses, the Env sequences of individuals who developed CrNA, position 337 is often occupied by a negatively charged amino acid (E), whereas this position is mostly positively charged (K) in individuals that did not develop CrNA. A similar charge reversal is observed for position 343 which interacts with a number of V4 residues. Thus, changes in electrostatic interactions may influence the interaction between C3 and V4. Moreover, Kirchherr *et al*. identified three amino acid substitutions in the V4 region (393G, 397G, and 413N) that were associated with greater neutralization potency and breadth [102]. Positions 393 and 397 are not observed in our SH-analysis but the neighboring residues, 396, 399 and 400, are. Position 413 is observed in our analysis and is part of a PNGS. Envs associated with CrNA more frequently had an Asparagine (N) at position 413. Gnanakaran *et al*. noted that the C3 α2-helix in clade B viruses is more hydrophobic and shielded from solvent by the V4 loop and glycosylation, while this domain is less hydrophobic, more exposed and more immunogenic in clade C viruses [101,103]. Collectively these findings imply that the way in which the V4 folds over and covers the α2-helix of C3 might affect the induction of bNAbs [77].

The impact of other residues highlighted by the SH-analysis is somewhat unclear. Several hits were found in C1 and C2, including the β-sandwich domain which is known to interact with gp41 [104]. The two sites identified by the SH-analysis at positions 461 and 463/464 in V5 are in close proximity to the CD4BS and might impact its accessibility. Two other sites which were significantly different between the two groups were at position 322 (V3) and 440 (C4). Position 322 is important for coreceptor usage, a conversion of a negatively charged residue to a positively charged residue is sufficient to switch a CCR5-using to a CXCR4-using virus [105,106]. All the sequences we used were derived from CCR5-using viruses (data not shown), ruling out any bias caused by differences in coreceptor usage. Interestingly Rosen *et al*. showed a very strong covariation between residues 322 and 440 that is influenced by charge [107], pointing at a potential functional electrostatic interaction between these two residues. If and how these changes contribute to the development of CrNA remains to be further studied.

We had hypothesized that early HIV-1 variants from individuals who later developed CrNA might have an increased sensitivity to bNAbs. We used multiple bNAbs covering four well known target epitopes, namely CD4BS (b12 and VRC01), gp120 outer domain (447-52D, 2G12, PGT121 and PGT126), quaternary V1V2 (PG9, PG16 and PGT145) and MPER (2F5 and 4E10). However, we did not observe a significantly increased sensitivity to neutralization by these bNAbs. One could argue that this lack of correlation is due to the fact that individuals with CrNA are analyzed as a group without considering the epitope specificity of their CrNA. Each individual might have developed different specificities, blurring the results for individual bNAbs. Sera of 14 of our individuals were previously analyzed for binding to a panel of gp120 core proteins and their corresponding CD4BS knockout mutants and showed the presence of CD4BS directing Abs [92], but we could not find a correlation between the binding of CD4BS Abs and neutralization sensitivity of early clonal virus variants to VRC01. Another explanation could be that the specificities responsible for CrNA in our individuals may be different from those of the monoclonal bNAbs we tested. We did observe differences in sensitivity to the polyclonal HIVIg: clonal viral variants from individuals who developed CrNA showed a trend towards being more sensitive to HIVIg might be consistent with a more exposed Env structure. In summary, we cannot conclude which bNAb epitopes were immunogenic in our study population.

In a previous study we showed that the kinetics of CrNA development coincided with the kinetics of the induction of autologous neutralizing response, which was directed against viruses isolated early after SC [34]. This suggests that the development of CrNA is driven by epitopes that are exposed on early viruses [80]. Alternatively, CrNA may be induced by early (or slightly later) HIV-1 variants that have already escaped from the initial autologous neutralizing response [77]. The latter observation is consistent with the hypothesis that HIV-1 escape variants selected by autologous NAbs, in turn, may elicit new NAbs with altered specificities that allow for broad reactivity [52]. We studied early Env variants from a relatively wide time span (2 to 14 months after SC), therefore we cannot rule out or support one of the above hypotheses.

Currently known bNAbs take years to develop and show accumulation of many somatic mutations, suggesting that their development is driven by years of antigen exposure and possibly reflecting the continuous co-evolution of HIV-1 and the immune response directed to it [51,52,80,108,109]. This would suggest a more prominent role for viral evolution and diversity in the development of bNAbs. Consistent with this, greater Env diversity early in infection was associated with greater NAb breadth later in infection in a cohort of antiretroviral therapy naïve Kenyan women that were mostly infected with subtype A [110]. In our present study, involving therapy naïve, subtype B infected MSM from the ACS; we did not observe a correlation between early Env diversity and the development of CrNA in serum (data not shown).

## Conclusions

In summary, our current results show that sequence and structural characteristics of Env from early subtype B HIV-1 viruses may be associated with the development of CrNA in serum during infection. We observed that the presence of a short V1 and lower probability of glycosylation, specifically at position 332, were associated with the induction of CrNA. In addition, a number of amino acid changes that mostly clustered in V1 and C3-V4 correlated with the development of CrNA. Additional studies are needed to further clarify the role of these amino acids in the induction of CrNA, but the determinants for CrNA induction described here might facilitate the design of vaccines aimed at inducing bNAbs.

## Methods

### Individuals and viruses

Samples studied here were derived from participants of the ACS of MSM who were infected with HIV-1 subtype B. From all ACS participants, 292 were previously tested for the presence of CrNA in their sera at 2–4 years post-SC [79]. Sera were tested for CrNA on a panel of 6 heterologous viruses from different subtypes [39] and ranked based on their geometric mean IC_50_ titer and on the number of viruses from the panel that were neutralized. For the present study we selected cohort participants for whom the date of seroconversion was known, who had a follow-up of at least 4 years, and were therapy naïve at the time of screening for CrNA. Moreover, clonal HIV-1 variants had to be available. These criteria were fulfilled by 32 individuals who seroconverted between 1984 and 1996 [35,40,79]. One of these individuals was defined as elite neutralizer, according to the definition of Simek *et al*. [39] with a geometric mean IC_50_ titer of 782. We excluded this individual from our study as it is the subject of other follow-up studies, leaving us with 31 individuals fulfilling our criteria (Table 1).

Clonal virus variants were previously obtained from co-cultures of peripheral blood mononuclear cells (PBMCs) from HIV-1 infected individuals and 3-day phytohemagglutinin (PHA) stimulated PBMCs from healthy donors [111,112]. As virus isolation by coculturing PBMCs from infected individuals with stimulated healthy donor PBMCs can result in selection of a variants that are more fit for replication in PBMCs *in vitro,* we used a protocol in which limiting numbers of PBMCs from infected individuals and stimulated healthy donor PBMCs were mixed in multiple parallel cultures. This allows for the isolation of multiple replication competent clonal variants, avoiding the outgrowth and loss of slowly replicating variants [111,112]. Moreover, to prevent sequence changes during *in vitro* culture, the number of passages in PBMCs was kept to a minimum. This method yields in clonal sequences that are very similar to sequences derived from single genome amplification [113]. The ACS are conducted in accordance with the ethical principles set out in the declaration of Helsinki, and written consent was obtained prior to data collection. The study was approved by the Academic Medical Center Institutional Medical Ethics Committee.

### Gp160 sequence analysis

The HIV env genes from proviral-DNA isolated from PBMCs, that were infected in vitro with a single clonal HIV-1 variant, were PCR amplified and subsequently sequenced as described previously [114-116]. Complete gp160 sequences could be analyzed for 26 individuals, whereas only C1-V5 env sequences could be analyzed for individuals ACH19566, 19342, 18860, 18839 and 18829 (Table 1). Nucleotide sequences were aligned using ClustalW in the software package of BioEdit [117], and edited manually. The reference sequence HXB2 was included in the alignment to number each aligned residue according to the corresponding position in this reference. Genetic analyses were performed on gp160 sequences starting at nucleotide position 91, thereby excluding the Env signal peptide. The total length of the gp160 sequences and the separate regions were calculated by counting the number of amino acids. The number of PNGS and number of NXT or NXS motifs were identified using N-Glycosite [118] at the Los Alamos HIV database website (http://www.hiv.lanl.gov/content/sequence/GLYCOSITE/glycosite.html). Sequences with double PNGS were counted by N-Glycosite as followed: NNSS as +1 NXS motif, NNTT as +1 NXT sequon, and NN[TS][ST] as +1 NXT motif. A proline at the second position (site pattern NP[ST]) is strongly disfavored for glycosylation and therefore excluded as a PNGS. Net electrostatic charges of gp160 were calculated by counting all charged amino acid residues per sequence, where residues R and K counted as +1, H as +0.293, and D and E as −1. Intra-individual genetic diversity of the complete Env sequences generated from the earliest time point were analyzed for 23 individuals, using the Kimura-2 parameter model of evolution in the MEGA 4.1 software package (http://www.megasoftware.net).

### Phylogenetic analyses

A Maximum Likelihood (ML) tree was constructed with complete HIV-1 env gp160 sequences from 26 individuals with diverse levels of CrNA. The best-fit nucleotide substitution model (GTR+I+G), selected by hierarchical likelihood ratio test (hLTRs, Model Test 3.7 [119] was implemented in the heuristic search for the best ML tree applying TBR branch-swapping algorithm using PAUP*4.0[120], starting with a Neighbor-Joining (NJ) tree constructed under the Hasegawa-Kishino-Yano (HKY85) model of evolution [121]. The robustness of the NJ phylogeny was assessed by bootstrap analysis with 1,000 rounds of replication.

### Neutralization assay

Neutralization sensitivity to known bNAbs and polyclonal HIVIg pools was tested in a PBMC based assay, for a minimum of one and a maximum of five clonal virus variants per individual. PBMCs were isolated from buffy coats obtained from healthy seronegative blood donors and cultured as described previously [112]. The neutralization sensitivity was tested for the twelve individuals who developed CrNA and the nine individuals who did not develop CrNA. The individuals who developed CrNA were included in this group following the criteria of having a geometric mean IC_50_ titer ≥ 90 in serum and the ability to neutralize ≥ 5 viruses from the panel of six heterologous viruses in a pseudovirus assay developed by Monogram Biosciences. The panel consisted of six pseudoviruses with envelope sequences from primary isolates of HIV-1 subtypes A (94UG103), B (92BR020 and JRCSF), C (93IN905 and MGRM-C-026) and CRF01_AE (92TH021) [35]. Individuals of whom the sera had a geometric mean IC_50_ titer ≤ 45 in serum and neutralized ≤ 2 viruses from the panel of 6 heterologous viruses were included in the group that did not develop CrNA (Table 1). In total eleven broadly reactive neutralizing monoclonal antibodies (bNAbs) were tested; IgG1 b12 (kindly provided by NIBSC; EVA3065), 2G12, 2F5 and 4E10 (kindly provided by NIBSC; ARP3277, EVA3063, ARP3239), PG9 and PG16 (AIDS reagent program #12149 and 12150), VRC01 (AIDS reagent program #12033), 447-52D (kindly provided by NIBSC; ARP3219)) and PGT121, PGT126 and PGT145 (kindly provided by IAVI NAC repository); and three pools of polyclonal HIVIg sera: pool 1 (AIDS reagent program #3957 lot. nr. 12–100158), pool 2 (AIDS reagent program #3957 lot. nr. 14–120074), and pool 3 (AIDS reagent program #3957 lot. nr. 11–098130; NABI lot. nr. IHV-50-111 and lot. nr. IHV-250-114 [122]). HIVIg is a pool of concentrated antibodies from the blood from HIV-positive asymptomatic persons with high levels of HIV-1 specific antibodies.

From each virus isolate, a final inoculum of 20 50% tissue culture infective doses (TCID50) was incubated for 1 h at 37°C with each specific monoclonal antibody in threefold serial dilution. The starting dilution was 25 μg/ml for b12, 2G12, 2F5, 4E10 and 447-52D, and 5 μg/ml for PG9, PG16, VRC01, PGT121, PGT126 and PGT145. To test the neutralization sensitivity of the virus clones against HIVIg a final inoculum of 20 TCID50 was incubated for 1 h at 37°C in threefold serial dilution with a starting dilution of 1500 μg/ml. Subsequently, 10^5^ PHA-stimulated PBMCs, derived from healthy blood donors, were added to the mixture. After incubation with HIVIg an additional washing step was performed with phosphate-buffered saline after 4 h of incubation at 37°C. On days 7 and 11, virus production in culture supernatants was analyzed with a p24 antigen capture enzyme-linked immunosorbent assay [123]. The percent neutralization was calculated by determining the reduction in p24 production in the presence of the bNAbs or HIVIg compared to the cultures with virus only. When possible, IC_50_s were determined by linear regression. When the analyses required the assignment of one IC_50_ value per individual, we took the median of the IC_50_ values of the individual virus clones. For calculations, viruses with IC_50_ values below the lowest dilution or above the highest dilution were assigned the lowest or highest dilution value, respectively.

### Statistical analyses

Statistical analyses on the gp160 sequence and the neutralization data were performed using Graphpad Prism v5.01. Differences between sequences (length, number of PNGS, NXS or NXT motifs and net electrostatic charge) were compared using a Spearman correlation test, with the geometric mean IC_50_ titer across the six heterologous viral panel as the absolute x-value. Differences and correlations were considered statistically significant when P values were ≤ 0.05. In cases of normal distribution of the data we used the unpaired t-test (for example to compare the mean neutralization titers for the eleven known bNAbs and polyclonal HIVIg pools between individuals that did or did not develop CrNA). When neutralization titers were not distributed normally, a Mann–Whitney test was performed to compare the two groups. For each individual, the median IC_50_ values of each bNAb or HIVIg were used. Difference in NIS vs. NLS glycosylation at position 332 and its correlation with the presence of CrNA was compared using a Fischer’s exact test.

### Multiple sequence alignment for Sequence Harmony

A multiple sequence alignment was performed on 91 Env sequences from 21 individuals, starting at nucleotide position 91, which excludes the Env signal peptide, with a minimum of one and a maximum of eleven sequences per individual. In total we obtained 58 sequences from twelve individuals who developed CrNA and 33 sequences from nine individuals that did not develop CrNA (Table 1). In order to create a Multiple Sequence Alignment of sufficient consistency in the variable regions, three of the most widely used tools, Praline, Muscle and Clustal [124-126], were used with a range of settings (different Pam/Blosum matrices, different gap open and gap extend penalties) and evaluated. None gave completely satisfactory results, so the final alignment was based on Praline with global pre-profiling and default settings. The variable regions were extensively adjusted manually using the Jalview alignment viewer and editor [126], by optimizing the alignment of sequence patterns such as the NXS/T glycosylation sites and guided by the conservation scores reported by Jalview, which are based on the conservation of physicochemical properties, without penalizing the introduction or elongation of gaps (Additional file 7: Figure S4).

### Comparison of viral sequences with Sequence Harmony

The Sequence Harmony (SH) method ([90] and http://www.ibi.vu.nl/programs/seqharmwww) was used to analyze amino acid differences between the env sequences of the twelve individuals who developed CrNA and the nine individuals who did not develop CrNA (Table 2). The SH algorithm is an entropy-based method, which detects positions within an alignment that display compositional differences in related protein sequences divided in two groups, and might therefore be linked to functional differences. In addition, an empirical Z-score is calculated, reflecting the significance of the SH-score obtained based on 100 random shuffling events of the sequences between the two groups. For details see reference [86] and the online documentation on the web server (http://www.ibi.vu.nl/programs/seqharmwww). SH measures the overlap in distribution of amino acid types between two subgroups (*A* and *B*; in this case *env* sequences obtained from individuals who developed CrNA and individuals who did not) at a certain position (*i*) in the sequence alignment as follows:

$$SH_{i}^{A/B}=\sum_{x}p_{i,x}^{A}\log\frac{p_{i,x}^{A}}{p_{i,x}^{A}+p_{i,x}^{B}}$$

where $p_{i,x}^{A}$ indicates the observed frequency in group A for amino acid type *x* at position *i* in the sequence, and $p_{i,x}^{B}$ analogously for amino acid frequencies observed in group B sequences. The final SH score is calculated by *SH*_*i*_ = ½ ( *SH*_*i*_^*A/B*^ + *SH*_*i*_^*B/A*^ ). Therefore, an SH score of 0 indicates amino acid positions that are specific for one of the sequence groups, whereas an SH score of 1 indicates a complete overlap at this amino acid position between the two groups. In our dataset, the number of sequences included per individual varies from 1 to 11, which makes the analysis biased towards individuals with larger numbers of sequences included. To adjust for this bias we extended the SH method by including a weight for each sequence. We assigned a weight *w*=1/*N*_*p*_ to each sequence as the inverse of the number of sequences *N*_*p*_ included for individual *p*. Each individual will therefore have the same impact on the total score in each group. Cut-off scores were set as SH<0.7 for the residues in the variable regions and <0.85 for residues in the conserved regions, i.e. a less strict selection in the conserved region to allow also small(er) differences to be detected. The lower (negative) the z-score, the less likely that the results were found by chance.

### Structural analysis

SH yielded a list of amino acid differences in Env between individuals who did developed CrNA and who did not, but structural analysis informs us on the location of these positions in the protein structure. A structure of the complete Env trimer is not available. In order to assist the inspection of the positions that were different between the two groups as identified by SH, we built a model that contained all gp120 domains. We started with the gp120 structure from 3JWD [104], because it contains the N- and C-termini of gp120. To this structure we added the V1V2 loops from 3U4E[64] and using 1GCG [127] to obtain an overlap with the 3JWD structure; as well as the V3 and V4 regions from 2B4C [128]. These structures were overlaid using the ‘super’ and ‘pair_fit’ functions of PyMol (The PyMOL Molecular Graphics System, Version 1.5.0.4 Schrödinger, LLC). No additional loop modeling was performed, so the actual conformation of the V1V2, V3 and the V4 regions as presented should be considered an arbitrary visualization of the residues identified by SH. Note that the glycans are absent from this model. The sites selected by the SH analysis (i.e., the residues scoring below the cut-off, as described above) were mapped on the three-dimensional structure of the resulting model using PyMol.

## Availability of supporting data

The sequences supporting the results of this article are available in the GenBank repository [Genbank: EU743974-743976; EU743978-743979; HQ6444871-644872; JF910158-910162; JF910166; JF910176-910178; JF910181-910183; JF910188-910190, http://www.ncbi.nlm.nih.gov/genbank].

## Abbreviations

HIV-1: Human immunodeficiency virus type-1; AIDS: Acquired immunodeficiency syndrome; CrNA: Cross-reactive neutralizing activity; NAbs: Neutralizing antibodies; Env: Envelope glycoprotein complex; PNGS: Potential N-linked glycosylation sites; post-SC: Post-seroconversion; CD4BS: CD4-binding site; bNAbs: Broadly reactive neutralizing monoclonal antibodies; (SH): Sequence Harmony; ACS: Amsterdam Cohort Studies on HIV infection and AIDS; MSM: Men who have sex with men; PBMCs: Peripheral blood mononuclear cells; PHA: Phytohemagglutinin; ML: Maximum Likelihood; NJ: Neighbor-Joining; TCID50: 50% tissue culture infective doses.

## Competing interests

The authors declare that they have no competing interests.

## Authors’ contributions

TLGMK and BDB performed the experiments and together with KAF, LWER and RWS contributed to data acquisition. TLGMK, KAF, LWER and RWS analyzed the data. TLGMK, KAF, ZE, MJG, JH, RWS and HS conceived and designed the study and experiments. TLGMK and RWS wrote and TLGMK, KAF, ZE, MJG, LWER, JH, HS, RWS edited the paper. All authors have read and approved the final manuscript.

## Supplementary Material

Additional file 1: Figure S1 Title of data: Baseline characteristics for the individuals with non-CrNA, intermediate CrNA and CrNA. Description of the data: (A) time, in months, between SC and CrNA level measured by Monogram Biosciences; (B) time, in months, between SC and isolation of clonal viral variants; (C) CD4^+^ T cells/μl blood at set-point; (D) viral load, in log10, at set-point. Individuals with non-CrNA, intermediate CrNA and CrNA in their serum are represented by circles, squares and triangles, respectively. Each individual is represented by one symbol. Click here for file

Additional file 2: Figure S2 Title of data: Genetic relationships between viruses from individuals with diverse levels of CrNA in serum. Description of data: Complete gp160 sequences derived from 26 individuals with varying levels of CrNA in serum were aligned and a ML tree was constructed. Individuals with high, intermediate and low CrNA are indicated in red, blue or green, respectively. Click here for file

Additional file 3: Table S1 Title of data: Sequence Harmony results with consensus sequences. Description of data: The Sequence Harmony (SH) method ([90] and http://www.ibi.vu.nl/programs/seqharmwww) was used to analyze amino acid differences between the consensus env sequences of the twelve individuals who developed CrNA and the nine individuals who did not develop CrNA, for details see Methods. The SH algorithm is an entropy-based method, which detects positions within an alignment that display compositional differences in related protein sequences divided in two groups, and might therefore be linked to functional differences. In addition, an empirical Z-score is calculated, reflecting the significance of the SH-score obtained based on 100 random shuffling events of the sequences between the two groups. Cut-off scores were set as SH<0.7 for the residues in the variable regions and <0.85 for residues in the conserved regions, i.e. a less strict selection in the conserved region to allow also small(er) differences to be detected. The lower (negative) the z-score, the less likely that the results were found by chance. Click here for file

Additional file 4: Figure S3 Title of data: Multiple sequence alignment of gp160 sequences from CrNA and non- CrNA individuals used for SH analysis. Description of data: Multiple sequence alignment of 91 complete gp160 sequences from 21 individuals, starting at nucleotide position 91, excluding the Env signal peptide, with a minimum of one and a maximum of eleven sequences per individual. In total 58 sequences from twelve individuals who developed CrNA and 33 sequences from nine individuals that did not develop CrNA, CrNA and non-CrNA respectively, are depicted. Click here for file

Additional file 5: Table S2 Title of data: Subclustering of positions identified by SH. Description of data: Six sites are isolated in the structure (and sequence) and form a cluster of their own. Three small clusters are purely sequential. Another sequential cluster (T138, N139, T140, N141, S142) is not present in any of the crystal structures analyzed, but aligns with a similar region (T138→T135, N139→I136, N141→N137 in 3U4E) which makes it cluster with another residue (K151 at 4Å). Finally, there are four other clusters containing sequentially distant residues with on average slightly more than four residues per cluster. In one case this clustering is tight, with a distance around 3Å, the three other distances involved are around 6-7Å. One of these clusters consists of three sequential parts and involves one tight and one looser distance. Most clusters are restricted to within one of the V or C regions, except for the one that connects C1 and C2, and the two others that bridge C3 and V4. Almost all residues selected within the V4 region are in close contact with the α2-helix (336–353) of the C3 region. Click here for file

Additional file 6: Table S3 Title of data: Median neutralization IC_50_ titer per individual per bNAb. Description of data: Median neutralization IC_50_ titer of the primary HIV-1 variants from 21 selected individuals to neutralization by eleven different bNAbs covering the four known epitope clusters, namely CD4BS (b12 and VRC01), gp120 outer domain (447-52D, 2G12, PGT121 and PGT126), quaternary V1V2 (PG9, PG16 and PGT145) and MPER (2F5 and 4E10), and to the three polyclonal HIVIg pools. Interestingly, early HIV-1 variants of individuals who developed CrNA were more sensitive to neutralization by polyclonal HIVIg pool 1 compared to early viruses from individuals who did not develop CrNA (p = 0.037), although this was found for the other two pools tested. Click here for file

Additional file 7: Figure S4 Title of data: Correlation of SH-scores and Z-scores. Description of data: (A) Correlation of the SH scores as found by Sequence Harmony; (B) Correlation of the Z-scores found by Sequence Harmony. Each amino acid difference in Env between individuals who did or did not developed CrNA is represented by one dot. The value on the x-axis represents scores found when using 91 Env sequences as described in Materials, and the value on the y-axis represents the scores found when using the consensus sequences of the 12 individuals who did and the 9 individuals who did not developed CrNA. Statistical analyses on the SH-scores and the Z-scores were performed using Graphpad Prism v5.01. Correlations between the two methods were compared using a Pearson correlation test. Differences and correlations were considered statistically significant when P values were ≤ 0.05. Click here for file

## References

[B1] Kim JH Rerks-Ngarm S Excler JL Michael NL HIV vaccines: lessons learned and the way forward Curr Opin HIV AIDS 2010 5 428 434 20978385 10.1097/COH.0b013e32833d17ac PMC2990218

[B2] Belshe RB Graham BS Keefer MC Gorse GJ Wright P Dolin R Matthews T Weinhold K Bolognesi DP Sposto R Neutralizing antibodies to HIV-1 in seronegative volunteers immunized with recombinant gp120 from the MN strain of HIV-1. NIAID AIDS Vaccine Clinical Trials Network JAMA 1994 272 475 480 7913731 10.1001/jama.272.6.475

[B3] Mascola JR Snyder SW Weislow OS Belay SM Belshe RB Schwartz DH Clements ML Dolin R Graham BS Gorse GJ Immunization with envelope subunit vaccine products elicits neutralizing antibodies against laboratory-adapted but not primary isolates of human immunodeficiency virus type 1 J Infect Dis 1996 173 340 348 8568294 10.1093/infdis/173.2.340

[B4] Pitisuttithum P Berman PW Phonrat B Suntharasamai P Raktham S Srisuwanvilai LO Hirunras K Kitayaporn D Kaewkangwal J Migasena S Phase I/II study of a candidate vaccine designed against the B and E subtypes of HIV-1 J Acquir Immune Defic Syndr 2004 37 1160 1165 15319676 10.1097/01.qai.0000136091.72955.4b

[B5] Rerks-Ngarm S Pitisuttithum P Nitayaphan S Kaewkungwal J Chiu J Paris R Premsri N Namwat C de Souza M Adams E Vaccination with ALVAC and AIDSVAX to Prevent HIV-1 Infection in Thailand N Engl J Med 2009 361 2209 2220 19843557 10.1056/NEJMoa0908492

[B6] Haynes BF Gilbert PB McElrath MJ Zolla-Pazner S Tomaras GD Alam SM Evans DT Montefiori DC Karnasuta C Sutthent R Immune-correlates analysis of an HIV-1 vaccine efficacy trial N Engl J Med 2012 366 1275 1286 22475592 10.1056/NEJMoa1113425 PMC3371689

[B7] Gelderblom HR Hausmann EHS Ozel M Pauli G Koch MA Fine structure of human immunodeficiency virus (HIV) and immunolocalization of structural proteins Virology 1987 156 171 176 3643678 10.1016/0042-6822(87)90449-1

[B8] Wei X Decker JM Wang S Hui H Kappes JC Wu X Salazar-Gonzalez JF Salazar MG Kilby JM Saag MS Antibody neutralization and escape by HIV-1 Nature 2003 422 307 312 12646921 10.1038/nature01470

[B9] Willey RL Rutledge RA Dias S Folks T Theodore T Buckler CE Martin MA Identification of conserved and divergent domains within the envelope gene of the acquired immunodeficiency syndrome retrovirus Proc Natl Acad Sci U S A 1986 83 5038 5042 3014529 10.1073/pnas.83.14.5038 PMC323885

[B10] Starcich BR Hahn BH Shaw GM McNeely PD Modrow S Wolf H Parks ES Parks WP Josephs SF Gallo RC Identification and characterization of conserved and variable regions in the envelope gene of HTLV-III/LAV, the retrovirus of AIDS Cell 1986 45 637 648 2423250 10.1016/0092-8674(86)90778-6

[B11] Cordonnier A Montagnier L Emerman M Single amino-acid changes in HIV envelope affect viral tropism and receptor binding Nature 1989 340 571 574 2475780 10.1038/340571a0

[B12] Kowalski M Potz J Basiripour L Dorfman T Goh WC Terwilliger E Dayton A Rosen G Haseltine WA Sodroski J Functional regions of the envelope glycoprotein of human immunodeficiency virus type I Science 1987 237 1351 1355 3629244 10.1126/science.3629244

[B13] Olshevsky U Helseth E Furman C Li J Haseltine W Sodroski J Identification of individual human immunodeficiency virus type 1 gp120 amino acids important for CD4 receptor binding J Virol 1990 64 5701 5707 2243375 10.1128/jvi.64.12.5701-5707.1990 PMC248709

[B14] Shehu-Xhilaga M Oelrichs RB Hoy J, Lewin S, Post J, Street A Basic HIV virology HIV Management in Australasia 2009 Darlinghurst: Australasian Society for HIV Medicine 9 18

[B15] Allan JS Coligan JE Bariun F Major glycoprotein antigens that induce antibodies in AIDS patients are encoded by HTLV-III Science 1985 228 1091 1093 2986290 10.1126/science.2986290

[B16] Binley JM Ban YE Crooks ET Eggink D Osawa K Schief WR Sanders RW Role of complex carbohydrates in human immunodeficiency virus type 1 infection and resistance to antibody neutralization J Virol 2010 84 5637 5655 20335257 10.1128/JVI.00105-10 PMC2876609

[B17] Geijtenbeek TB Kwon DS Torensma R Van Vliet SJ Van Duijnhoven GC Middel J Cornelissen IL Nottet HS KewalRamani VN Littman DR DC-SIGN, a dendritic cell-specific HIV-1-binding protein that enhances trans-infection of T cells Cell 2000 100 491 494 10721995 10.1016/s0092-8674(00)80694-7

[B18] Montefiori DC Robinson WE Jr Mitchell WM Role of protein N-glycosylation in pathogenesis of human immunodeficiency virus type 1 Proc Natl Acad Sci U S A 1988 85 9248 9252 3264072 10.1073/pnas.85.23.9248 PMC282716

[B19] Reitter JN Means RE Desrosiers RC A role for carbohydrates in immune evasion in AIDS Nat Med 1998 4 679 684 9623976 10.1038/nm0698-679

[B20] Walker BD Kowalski M Goh WC Kozarsky K Krieger M Rosen C Rohrschneider L Haseltine WA Sodroski J Inhibition of human immunodeficiency virus syncytium formation and virus replication by castanospermine Proc Natl Acad Sci U S A 1987 84 8120 8124 2825177 10.1073/pnas.84.22.8120 PMC299490

[B21] Aasa-Chapman MM Cheney KM Hue S Forsman A O'Farrell S Pellegrino P Williams I McKnight A In vivo emergence of HIV-1 highly sensitive to neutralizing antibodies PLoS One 2011 6 e23961 21887353 10.1371/journal.pone.0023961 PMC3161086

[B22] Bunnik EM Pisas L van Nuenen AC Schuitemaker H Autologous neutralizing humoral immunity and evolution of the viral envelope in the course of subtype B human immunodeficiency virus type 1 infection J Virol 2008 82 7932 7941 18524815 10.1128/JVI.00757-08 PMC2519599

[B23] Montefiori DC Morris L Ferrari G Mascola JR Neutralizing and other antiviral antibodies in HIV-1 infection and vaccination Curr Opin HIV AIDS 2007 2 169 176 19372883 10.1097/COH.0b013e3280ef691e PMC3171201

[B24] Richman DD Wrin T Little SJ Petropoulos CJ Rapid evolution of the neutralizing antibody response to HIV type 1 infection Proc Natl Acad Sci U S A 2003 100 4144 4149 12644702 10.1073/pnas.0630530100 PMC153062

[B25] van Gils MJ Bunnik EM Burger JA Jacob Y Schweighardt B Wrin T Schuitemaker H Rapid escape from preserved cross-reactive neutralizing humoral immunity without loss of viral fitness in HIV-1-infected progressors and long-term nonprogressors J Virol 2010 84 3576 3585 20071586 10.1128/JVI.02622-09 PMC2838121

[B26] Tomaras GD Yates NL Liu P Qin L Fouda GG Chavez LL Decamp AC Parks RJ Ashley VC Lucas JT Initial B-cell responses to transmitted human immunodeficiency virus type 1: virion-binding immunoglobulin M (IgM) and IgG antibodies followed by plasma anti-gp41 antibodies with ineffective control of initial viremia J Virol 2008 82 12449 12463 18842730 10.1128/JVI.01708-08 PMC2593361

[B27] Davis KL Gray ES Moore PL Decker JM Salomon A Montefiori DC Graham BS Keefer MC Pinter A Morris L High titer HIV-1 V3-specific antibodies with broad reactivity but low neutralizing potency in acute infection and following vaccination Virology 2009 387 414 426 19298995 10.1016/j.virol.2009.02.022 PMC2792036

[B28] Cao J Sullivan N Desjardin E Parolin C Robinson J Wyatt R Sodroski J Replication and neutralization of human immunodeficiency virus type 1 lacking the V1 and V2 variable loops of the gp120 envelope glycoprotein J Virol 1997 71 9808 9812 9371651 10.1128/jvi.71.12.9808-9812.1997 PMC230295

[B29] Curlin ME Zioni R Hawes SE Liu Y Deng W Gottlieb GS Zhu T Mullins JI HIV-1 envelope subregion length variation during disease progression PLoS Pathog 2010 6 e1001228 21187897 10.1371/journal.ppat.1001228 PMC3002983

[B30] Sagar M Wu X Lee S Overbaugh J Human immunodeficiency virus type 1 V1-V2 envelope loop sequences expand and add glycosylation sites over the course of infection, and these modifications affect antibody neutralization sensitivity J Virol 2006 80 9586 9598 16973562 10.1128/JVI.00141-06 PMC1617272

[B31] Stamatatos L Cheng-Mayer C An envelope modification that renders a primary, neutralization-resistant clade B human immunodeficiency virus type 1 isolate highly susceptible to neutralization by sera from other clades J Virol 1998 72 7840 7845 9733820 10.1128/jvi.72.10.7840-7845.1998 PMC110102

[B32] van Gils MJ Bunnik EM Boeser-Nunnink BD Burger JA Terlouw-Klein M Verwer N Schuitemaker H Longer V1V2 region with increased number of potential N-linked glycosylation sites in the HIV-1 envelope glycoprotein protects against HIV-specific neutralizing antibodies J Virol 2011 85 6986 6995 21593147 10.1128/JVI.00268-11 PMC3126602

[B33] Doria-Rose NA Klein RM Manion MM O'Dell S Phogat A Chakrabarti B Hallahan CW Migueles SA Wrammert J Ahmed R Frequency and phenotype of human immunodeficiency virus envelope-specific B cells from patients with broadly cross-neutralizing antibodies J Virol 2009 83 188 199 18922865 10.1128/JVI.01583-08 PMC2612342

[B34] Euler Z van den Kerkhof TL van Gils MJ Burger JA Edo-Matas D Phung P Wrin T Schuitemaker H Longitudinal analysis of early HIV-1 specific neutralizing activity in an elite neutralizer and in five patients who developed cross-reactive neutralizing activity J Virol 2012 86 2045 2055 22156522 10.1128/JVI.06091-11 PMC3302374

[B35] Euler Z van Gils MJ Bunnik EM Phung P Schweighardt B Wrin T Schuitemaker H Cross-reactive neutralizing humoral immunity does not protect from HIV type 1 disease progression J Infect Dis 2010 201 1045 1053 20170371 10.1086/651144

[B36] Gray ES Madiga MC Hermanus T Moore PL Wibmer CK Tumba NL Werner L Mlisana K Sibeko S Williamson C The neutralization breadth of HIV-1 develops incrementally over four years and is associated with CD4+ T cell decline and high viral load during acute infection J Virol 2011 85 4828 4840 21389135 10.1128/JVI.00198-11 PMC3126191

[B37] Mikell I Sather DN Kalams SA Altfeld M Alter G Stamatatos L Characteristics of the Earliest Cross-Neutralizing Antibody Response to HIV-1 PLoS Pathog 2011 7 e1001251 21249232 10.1371/journal.ppat.1001251 PMC3020924

[B38] Sather DN Armann J Ching LK Mavrantoni A Sellhorn G Caldwell Z Yu X Wood B Self S Kalams S Factors associated with the development of cross-reactive neutralizing antibodies during human immunodeficiency virus type 1 infection J Virol 2009 83 757 769 18987148 10.1128/JVI.02036-08 PMC2612355

[B39] Simek MD Rida W Priddy FH Pung P Carrow E Laufer DS Lehrman JK Boaz M Tarragona-Fiol T Miiro G HIV-1 Elite Neutralizers: Individuals with Broad and Potent Neutralizing Activity Identified Using a High Throughput Neutralization Assay Together with an Analytical Selection Algorithm J Virol 2009 83 7337 7348 19439467 10.1128/JVI.00110-09 PMC2704778

[B40] van Gils MJ Euler Z Schweighardt B Wrin T Schuitemaker H Prevalence of cross-reactive HIV-1-neutralizing activity in HIV-1-infected patients with rapid or slow disease progression AIDS 2009 23 2405 2414 19770692 10.1097/QAD.0b013e32833243e7

[B41] Binley JM Lybarger EA Crooks ET Seaman MS Gray E Davis KL Decker JM Wycuff D Harris L Hawkins N Profiling the specificity of neutralizing antibodies in a large panel of plasmas from patients chronically infected with human immunodeficiency virus type 1 subtypes B and C J Virol 2008 82 11651 11668 18815292 10.1128/JVI.01762-08 PMC2583680

[B42] Li Y Migueles SA Welcher B Svehla K Phogat A Louder MK Wu X Shaw GM Connors M Wyatt RT Broad HIV-1 neutralization mediated by CD4-binding site antibodies Nat Med 2007 13 1032 1034 17721546 10.1038/nm1624 PMC2584972

[B43] Frost SD Wrin T Smith DM Kosakovsky Pond SL Liu Y Paxinos E Chappey C Galovich J Beauchaine J Petropoulos CJ Neutralizing antibody responses drive the evolution of human immunodeficiency virus type 1 envelope during recent HIV infection Proc Natl Acad Sci U S A 2005 102 18514 18519 16339909 10.1073/pnas.0504658102 PMC1310509

[B44] Euler Z Schuitemaker H Cross-reactive broadly neutralizing antibodies: timing is everything Front Immunol 2012 3 215 22833745 10.3389/fimmu.2012.00215 PMC3400945

[B45] Hessell AJ Poignard P Hunter M Hangartner L Tehrani DM Bleeker WK Parren PW Marx PA Burton DR Effective, low-titer antibody protection against low-dose repeated mucosal SHIV challenge in macaques Nat Med 2009 15 951 954 19525965 10.1038/nm.1974 PMC4334439

[B46] Hessell AJ Rakasz EG Poignard P Hangartner L Landucci G Forthal DN Koff WC Watkins DI Burton DR Broadly neutralizing human anti-HIV antibody 2G12 is effective in protection against mucosal SHIV challenge even at low serum neutralizing titers PLoS Pathog 2009 5 e1000433 19436712 10.1371/journal.ppat.1000433 PMC2674935

[B47] Hessell AJ Rakasz EG Tehrani DM Huber M Weisgrau KL Landucci G Forthal DN Koff WC Poignard P Watkins DI Broadly neutralizing monoclonal antibodies 2F5 and 4E10 directed against the human immunodeficiency virus type 1 gp41 membrane-proximal external region protect against mucosal challenge by simian-human immunodeficiency virus SHIVBa-L J Virol 2010 84 1302 1313 19906907 10.1128/JVI.01272-09 PMC2812338

[B48] Mascola JR Lewis MG Stiegler G Harris D VanCott TC Hayes D Louder MK Brown CR Sapan CV Frankel SS Protection of Macaques against pathogenic simian/human immunodeficiency virus 89.6PD by passive transfer of neutralizing antibodies J Virol 1999 73 4009 4018 10196297 10.1128/jvi.73.5.4009-4018.1999 PMC104180

[B49] Shibata R Igarashi T Haigwood N Buckler-White A Ogert R Ross W Willey R Cho MW Martin MA Neutralizing antibody directed against the HIV-1 envelope glycoprotein can completely block HIV-1/SIV chimeric virus infection of macaque monkeys Nat Med 1999 5 204 210 9930869 10.1038/5568

[B50] van Gils MJ Sanders RW Broadly neutralizing antibodies against HIV-1: templates for a vaccine Virology 2013 435 46 56 23217615 10.1016/j.virol.2012.10.004

[B51] Scheid JF Mouquet H Ueberheide B Diskin R Klein F Olivera TY Pietzsch J Fenyo D Abadir A Velinzon K Sequence and Structural Convergence of Broad and Potent HIV Antibodies That Mimic CD4 Binding Science 2011 333 1633 1637 21764753 10.1126/science.1207227 PMC3351836

[B52] Walker LM Huber M Doores KJ Falkowska E Pejchal R Julien JP Wang SK Ramos A Chan-Hui PY Moyle M Broad neutralization coverage of HIV by multiple highly potent antibodies Nature 2011 477 466 470 21849977 10.1038/nature10373 PMC3393110

[B53] Wu X Yang ZY Li Y Hogerkorp CM Schief WR Seaman MS Zhou T Schmidt SD Wu L Xu L Rational design of envelope identifies broadly neutralizing human monoclonal antibodies to HIV-1 Science 2010 329 856 861 20616233 10.1126/science.1187659 PMC2965066

[B54] Burton DR Pyati J Koduri R Sharp SJ Thornton GB Parren PWH Sawyer LSW Hendry RM Dunlop N Nara PL Efficient neutralization of primary isolates of HIV-1 by a recombinant human monoclonal antibody Science 1994 266 1024 1027 7973652 10.1126/science.7973652

[B55] Corti D Langedijk JP Hinz A Seaman MS Vanzetta F Fernandez-Rodriguez BM Silacci C Pinna D Jarrossay D Balla-Jhagjhoorsingh S Analysis of memory B cell responses and isolation of novel monoclonal antibodies with neutralizing breadth from HIV-1-infected individuals PLoS One 2010 5 e8805 20098712 10.1371/journal.pone.0008805 PMC2808385

[B56] Zhou T Georgiev I Wu X Yang ZY Dai K Finzi A Do KY Scheid J Shi W Xu L Structural Basis for Broad and Potent Neutralization of HIV-1 by Antibody VRC01 Science 2010 329 811 817 20616231 10.1126/science.1192819 PMC2981354

[B57] Zhou T Xu L Dey B Hessell AJ Van RD Xiang SH Yang X Zhang MY Zwick MB Arthos J Structural definition of a conserved neutralization epitope on HIV-1 gp120 Nature 2007 445 732 737 17301785 10.1038/nature05580 PMC2584968

[B58] Diskin R Scheid JF Marcovecchio PM West AP Jr Klein F Gao H Gnanapragasam PN Abadir A Seaman MS Nussenzweig MC Increasing the potency and breadth of an HIV antibody by using structure-based rational design Science 2011 334 1289 1293 22033520 10.1126/science.1213782 PMC3232316

[B59] Julien JP Sok D Khayat R Lee JH Doores KJ Walker LM Ramos A Diwanji DC Pejchal R Cupo A Broadly Neutralizing Antibody PGT121 Allosterically Modulates CD4 Binding via Recognition of the HIV-1 gp120 V3 Base and Multiple Surrounding Glycans PLoS Pathog 2013 9 e1003342 23658524 10.1371/journal.ppat.1003342 PMC3642082

[B60] Pejchal R Doores KJ Walker LM Khayat R Huang PS Wang SK Stanfield RL Julien JP Ramos A Crispin M A potent and broad neutralizing antibody recognizes and penetrates the HIV glycan shield Science 2011 334 1097 1103 21998254 10.1126/science.1213256 PMC3280215

[B61] Sanders RW Venturi M Schiffner L Kalyanaraman R Katinger H Lloyd KO Kwong PD Moore JP The Mannose-Dependent Epitope for Neutralizing Antibody 2G12 on Human Immunodeficiency Virus Type 1 Glycoprotein gp120 J Virol 2002 76 7293 7305 12072528 10.1128/JVI.76.14.7293-7305.2002 PMC136300

[B62] Scanlan CN Pantophlet R Wormald MR Ollman Saphire E Stanfield R Wilson IA Katinger H Dwek RA Rudd PM Burton DR The Broadly Neutralizing Anti-Human Immunodeficiency Virus Type 1 Antibody 2G12 Recognizes a Cluster of alfa1->2 Mannose Residues on the Outer Face of gp120 J Virol 2002 76 7306 7321 12072529 10.1128/JVI.76.14.7306-7321.2002 PMC136327

[B63] Trkola A Purtscher M Muster T Ballaun C Buchacher A Sullivan N Srinivasan K Sodroski J Moore JP Katinger H Human monoclonal antibody 2G12 defines a distinctive neutralization epitope on the gp120 glycoprotein of human immunodeficiency virus type 1 J Virol 1996 70 1100 1108 8551569 10.1128/jvi.70.2.1100-1108.1996 PMC189917

[B64] McLellan JS Pancera M Carrico C Gorman J Julien JP Khayat R Louder R Pejchal R Sastry M Dai K Structure of HIV-1 gp120 V1/V2 domain with broadly neutralizing antibody PG9 Nature 2011 480 336 343 22113616 10.1038/nature10696 PMC3406929

[B65] Walker LM Phogat SK Chan-Hui PY Wagner D Phung P Goss JL Wrin T Simek MD Fling S Mitcham JL Broad and Potent Neutralizing Antibodies from an African Donor Reveal a New HIV-1 Vaccine Target Science 2009 326 285 289 19729618 10.1126/science.1178746 PMC3335270

[B66] Bonsignori M Hwang KK Chen X Tsao CY Morris L Gray E Marshall DJ Crump JA Kapiga SH Sam NE Analysis of a Clonal Lineage of HIV-1 Envelope V2/V3 Conformational Epitope-Specific Broadly Neutralizing Antibodies and Their Inferred Unmutated Common Ancestors J Virol 2011 85 9998 10009 21795340 10.1128/JVI.05045-11 PMC3196428

[B67] Julien JP Lee JH Cupo A Murin CD Derking R Hoffenberg S Caulfield MJ King CR Marozsan AJ Klasse PJ Asymmetric recognition of the HIV-1 trimer by broadly neutralizing antibody PG9 Proc Natl Acad Sci U S A 2013 110 4351 4356 23426631 10.1073/pnas.1217537110 PMC3600498

[B68] Doores KJ Burton DR Variable loop glycan dependency of the broad and potent HIV-1-neutralizing antibodies PG9 and PG16 J Virol 2010 84 10510 10521 20686044 10.1128/JVI.00552-10 PMC2950566

[B69] Alam SM Morelli M Dennison SM Liao HX Zhang R Xia SM Rits-Volloch S Sun L Harrison SC Haynes BF Role of HIV membrane in neutralization by two broadly neutralizing antibodies Proc Natl Acad Sci U S A 2009 106 20234 20239 19906992 10.1073/pnas.0908713106 PMC2787149

[B70] Muster T Steindl F Purtscher M Trkola A Klima A Himmler G Ruker F Katinger H A conserved neutralizing epitope on gp41 of human immunodeficiency virus type 1 J Virol 1993 67 6642 6647 7692082 10.1128/jvi.67.11.6642-6647.1993 PMC238102

[B71] Stiegler G Kunert R Purtscher M Wolbank S Voglauer R Steindl F Katinger H A potent cross-clade neutralizing human monoclonal antibody against a novel epitope on gp41 of human immunodeficiency virus type 1 AIDS Res Hum Retroviruses 2001 17 1757 1765 11788027 10.1089/08892220152741450

[B72] Huang J Ofek G Laub L Louder MK Doria-Rose NA Longo NS Imamichi H Bailer RT Chakrabarti B Sharma SK Broad and potent neutralization of HIV-1 by a gp41-specific human antibody Nature 2012 491 406 412 23151583 10.1038/nature11544 PMC4854285

[B73] Wyatt R Kwong PD Desjardins E Sweet RW Robinson J Hendrickson WA Sodroski J The antigenic structure of the HIV gp120 envelope protein Nature 1998 393 705 711 9641684 10.1038/31514

[B74] Kong L Lee JH Doores KJ Murin CD Julien JP McBride R Liu Y Marozsan A Cupo A Klasse PJ Supersite of immune vulnerability on the glycosylated face of HIV-1 envelope glycoprotein gp120 Nat Struct Mol Biol 2013 20 796 803 23708606 10.1038/nsmb.2594 PMC3823233

[B75] Walker LM Simek MD Priddy F Gach JS Wagner D A limited number of antibody specificities mediate broad and potent serum neutralization in selected HIV-1 infected individuals PLoS Pathog 2010 6 8 e1001028 20700449 10.1371/journal.ppat.1001028 PMC2916884

[B76] Walker LM Sok D Nishimura Y Donau O Sadjadpour R Gautam R Shingai M Pejchal R Ramos A Simek MD Rapid development of glycan-specific, broad, and potent anti-HIV-1 gp120 neutralizing antibodies in an R5 SIV/HIV chimeric virus infected macaque Proc Natl Acad Sci U S A 2011 108 20125 20129 22123961 10.1073/pnas.1117531108 PMC3250170

[B77] Moore PL Gray ES Wibmer CK Bhiman JN Nonyane M Sheward DJ Hermanus T Bajimaya S Tumba NL Abrahams MR Evolution of an HIV glycan-dependent broadly neutralizing antibody epitope through immune escape Nat Med 2012 8 1688 1692 23086475 10.1038/nm.2985 PMC3494733

[B78] Walker BD Burton DR Toward an AIDS vaccine Science 2008 320 760 764 18467582 10.1126/science.1152622

[B79] Euler Z van Gils MJ Boeser-Nunnink BD Schuitemaker H van Manen D Genome-wide association study on the development of cross-reactive neutralizing antibodies in HIV-1 infected individuals PLoS One 2013 8 e54684 23372753 10.1371/journal.pone.0054684 PMC3553002

[B80] Liao HX Lynch R Zhou T Gao F Alam SM Boyd SD Fire AZ Roskin KM Schramm CA Zhang Z Co-evolution of a broadly neutralizing HIV-1 antibody and founder virus Nature 2013 10 469 476 23552890 10.1038/nature12053 PMC3637846

[B81] Binley JM Wrin T Korber B Zwick MB Wang M Chappey C Stiegler G Kunert R Zolla-Pazner S Katinger H Comprehensive cross-clade neutralization analysis of a panel of anti-human immunodeficiency virus type 1 monoclonal antibodies J Virol 2004 78 13232 13252 15542675 10.1128/JVI.78.23.13232-13252.2004 PMC524984

[B82] Gavel Y von Heijne G Sequence differences between glycosylated and non-glycosylated Asn-X-Thr/Ser acceptor sites: implications for protein engineering Protein Eng 1990 3 433 442 2349213 10.1093/protein/3.5.433 PMC7529082

[B83] Kaplan HA Welply JK Lennarz WJ Oligosaccharyl transferase: the central enzyme in the pathway of glycoprotein assembly Biochim Biophys Acta 1987 906 161 173 3297152 10.1016/0304-4157(87)90010-4

[B84] Shakin-Eshleman SH Spitalnik SL Kasturi L The amino acid at the X position of an Asn-X-Ser sequon is an important determinant of N-linked core-glycosylation efficiency J Biol Chem 1996 271 6363 6366 8626433 10.1074/jbc.271.11.6363

[B85] Sanders RW Van AE Nabatov AA Liscaljet IM Bontjer I Eggink D Melchers M Busser E Dankers MM Groot F The carbohydrate at asparagine 386 on HIV-1 gp120 is not essential for protein folding and function but is involved in immune evasion Retrovirology 2008 5 10 18237398 10.1186/1742-4690-5-10 PMC2262092

[B86] Pirovano W Feenstra KA Heringa J Sequence comparison by sequence harmony identifies subtype-specific functional sites Nucleic Acids Res 2006 34 6540 6548 17130172 10.1093/nar/gkl901 PMC1702503

[B87] Liu J Bartesaghi A Borgnia MJ Sapiro G Subramaniam S Molecular architecture of native HIV-1 gp120 trimers Nature 2008 455 109 113 18668044 10.1038/nature07159 PMC2610422

[B88] Finzi A Xiang SH Pacheco B Wang L Haight J Kassa A Danek B Pancera M Kwong PD Sodroski J Topological layers in the HIV-1 gp120 inner domain regulate gp41 interaction and CD4-triggered conformational transitions Mol Cell 2010 37 656 667 20227370 10.1016/j.molcel.2010.02.012 PMC2854584

[B89] Gnanakaran S Daniels MG Bhattacharya T Lapedes AS Sethi A Li M Tang H Greene K Gao H Haynes BF Genetic signatures in the envelope glycoproteins of HIV-1 that associate with broadly neutralizing antibodies PLoS Comput Biol 2010 6 e1000955 20949103 10.1371/journal.pcbi.1000955 PMC2951345

[B90] Rademeyer C Moore PL Taylor N Martin DP Choge IA Gray ES Sheppard HW Gray C Morris L Williamson C Genetic characteristics of HIV-1 subtype C envelopes inducing cross-neutralizing antibodies Virology 2007 368 172 181 17632196 10.1016/j.virol.2007.06.013

[B91] Wyatt R Moore J Accola M Desjardin E Robinson J Sodroski J Involvement of the V1/V2 variable loop structure in the exposure of Human Immunodeficiency Virus type 1 gp120 epitopes induced by receptor binding J Virol 1995 69 5723 5733 7543586 10.1128/jvi.69.9.5723-5733.1995 PMC189432

[B92] Laakso MM Lee FH Haggarty B Agrawal C Nolan KM Biscone M Romano J Jordan AP Leslie GJ Meissner EG V3 loop truncations in HIV-1 envelope impart resistance to coreceptor inhibitors and enhanced sensitivity to neutralizing antibodies PLoS Pathog 2007 3 e117 17722977 10.1371/journal.ppat.0030117 PMC1950945

[B93] Liu L Cimbro R Lusso P Berger EA Intraprotomer masking of third variable loop (V3) epitopes by the first and second variable loops (V1V2) within the native HIV-1 envelope glycoprotein trimer Proc Natl Acad Sci U S A 2011 108 20148 20153 22128330 10.1073/pnas.1104840108 PMC3250183

[B94] Rusert P Krarup A Magnus C Brandenberg OF Weber J Ehlert AK Regoes RR Gunthard HF Trkola A Interaction of the gp120 V1V2 loop with a neighboring gp120 unit shields the HIV envelope trimer against cross-neutralizing antibodies J Exp Med 2011 208 1419 1433 21646396 10.1084/jem.20110196 PMC3135368

[B95] Sanders RW Schiffner L Master A Kajumo F Guo Y Dragic T Moore JP Binley JM Variable-loop-deleted variants of the human immunodeficiency virus type 1 envelope glycoprotein can be stabilized by an intermolecular disulfide bond between the gp120 and gp41 subunits J Virol 2000 74 5091 5100 10799583 10.1128/jvi.74.11.5091-5100.2000 PMC110861

[B96] Saunders CJ McCaffrey RA Zharkikh I Kraft Z Malenbaum SE Burke B Cheng-Mayer C Stamatatos L The V1, V2, and V3 regions of the human immunodeficiency virus type 1 envelope differentially affect the viral phenotype in an isolate-dependent manner J Virol 2005 79 9069 9080 15994801 10.1128/JVI.79.14.9069-9080.2005 PMC1168758

[B97] Bunnik EM Euler Z Welkers MR Boeser-Nunnink BD Grijsen ML Prins JM Schuitemaker H Adaptation of HIV-1 envelope gp120 to humoral immunity at a population level Nat Med 2010 16 995 997 20802498 10.1038/nm.2203

[B98] Li M Salazar-Gonzalez JF Derdeyn CA Morris L Williamson C Robinson JE Decker JM Li Y Salazar MG Polonis VR Genetic and Neutralization Properties of Acute and Early Subtype C Human Immunodeficiency Virus Type 1 Molecular env Clones from Heterosexually Acquired Infections in Southern Africa J Virol 2006 Epub ahead of print:doi:10.1128/JVI.01730-06 10.1128/JVI.01730-06 PMC1642599 16971434

[B99] Rong R Bibollet-Ruche F Mulenga J Allen S Blackwell JL Derdeyn CA Role of V1V2 and other human immunodeficiency virus type 1 envelope domains in resistance to autologous neutralization during clade C infection J Virol 2007 81 1350 1359 17079307 10.1128/JVI.01839-06 PMC1797511

[B100] Jenkins N Parekh RB James DC Getting the glycosylation right: implications for the biotechnology industry Nat Biotechnol 1996 14 975 981 9631034 10.1038/nbt0896-975

[B101] Gnanakaran S Lang D Daniels M Bhattacharya T Derdeyn CA Korber B Clade-specific differences between human immunodeficiency virus type 1 clades B and C: diversity and correlations in C3-V4 regions of gp120 J Virol 2007 81 4886 4891 17166900 10.1128/JVI.01954-06 PMC1900169

[B102] Kirchherr JL Hamilton J Lu X Gnanakaran S Muldoon M Daniels M Kasongo W Chalwe V Mulenga C Mwananyanda L Identification of amino acid substitutions associated with neutralization phenotype in the human immunodeficiency virus type-1 subtype C gp120 Virology 2011 409 163 174 21036380 10.1016/j.virol.2010.09.031 PMC3007099

[B103] Moore PL Ranchobe N Lambson BE Gray ES Cave E Abrahams MR Bandawe G Mlisana K bdool Karim SS Williamson C Limited neutralizing antibody specificities drive neutralization escape in early HIV-1 subtype C infection PLoS Pathog 2009 5 e1000598 19763271 10.1371/journal.ppat.1000598 PMC2742164

[B104] Pancera M Majeed S Ban YE Chen L Huang CC Kong L Kwon YD Stuckey J Zhou T Robinson JE Structure of HIV-1 gp120 with gp41-interactive region reveals layered envelope architecture and basis of conformational mobility Proc Natl Acad Sci U S A 2010 107 1166 1171 20080564 10.1073/pnas.0911004107 PMC2824281

[B105] De Jong JJ De Ronde A Keulen W Tersmette M Goudsmit J Minimal requirements for the human immunodeficiency virus type 1 V3 domain to support the syncytium-inducing phenotype: analysis by single amino acid substitution J Virol 1992 66 6777 6780 1404617 10.1128/jvi.66.11.6777-6780.1992 PMC240176

[B106] Fouchier RAM Groenink M Kootstra NA Tersmette M Huisman HG Miedema F Schuitemaker H Phenotype-associated sequence variation in the third variable domain of the human immunodeficiency virus type 1 gp120 molecule J Virol 1992 66 3183 3187 1560543 10.1128/jvi.66.5.3183-3187.1992 PMC241084

[B107] Rosen O Samson AO Anglister J Correlated mutations at gp120 positions 322 and 440: implications for gp120 structure Proteins 2008 71 1066 1070 18275085 10.1002/prot.21982

[B108] Moir S Malaspina A Fauci AS Prospects for an HIV vaccine: leading B cells down the right path Nat Struct Mol Biol 2011 18 1317 1321 22139037 10.1038/nsmb.2194

[B109] Wu X Zhou T Zhu J Zhang B Georgiev I Wang C Chen X Longo NS Louder M McKee K Focused Evolution of HIV-1 Neutralizing Antibodies Revealed by Structures and Deep Sequencing Science 2011 333 1593 1602 21835983 10.1126/science.1207532 PMC3516815

[B110] Piantadosi A Panteleeff D Blish CA Baeten JM Jaoko W McClelland RS Overbaugh J Breadth of neutralizing antibody response to human immunodeficiency virus type 1 is affected by factors early in infection but does not influence disease progression J Virol 2009 83 10269 10274 19640996 10.1128/JVI.01149-09 PMC2748011

[B111] Schuitemaker H Koot M Kootstra NA Dercksen MW de Goede RE Van Steenwijk RP Lange JM Schattenkerk JK Miedema F Tersmette M Biological phenotype of human immunodeficiency virus type 1 clones at different stages of infection: progression of disease is associated with a shift from monocytotropic to T-cell-tropic virus population J Virol 1992 66 1354 1360 1738194 10.1128/jvi.66.3.1354-1360.1992 PMC240857

[B112] Van 't Wout AB Schuitemaker H Kootstra NA Isolation and propagation of HIV-1 on peripheral blood mononuclear cells Nat Protoc 2008 3 363 370 18323807 10.1038/nprot.2008.3

[B113] Edo-Matas D van Gils MJ Bowles EJ Navis M Rachinger A Boeser-Nunnink B Stewart-Jones GB Kootstra NA Wout AB Schuitemaker H Genetic composition of replication competent clonal HIV-1 variants isolated from peripheral blood mononuclear cells (PBMC), HIV-1 proviral DNA from PBMC and HIV-1 RNA in serum in the course of HIV-1 infection Virology 2010 405 492 504 20638697 10.1016/j.virol.2010.06.029

[B114] Beaumont T van Nuenen A Broersen S Blattner WA Lukashov VV Schuitemaker H Reversal of HIV-1 IIIB towards a neutralization resistant phenotype in an accidentally infected laboratory worker with a progressive clinical course J Virol 2001 75 2246 2252 11160728 10.1128/JVI.75.5.2246-2252.2001 PMC114808

[B115] Boom R Sol CJA Salimans MMM Jansen CL Wertheim-van Dillen PME Van der Noordaa J A rapid and simple method for purification of nucleic acids J Clin Microbiol 1991 28 495 503 1691208 10.1128/jcm.28.3.495-503.1990 PMC269651

[B116] Quakkelaar ED van Alphen FP Boeser-Nunnink BD van Nuenen AC Pantophlet R Schuitemaker H Susceptibility of recently transmitted subtype B human immunodeficiency virus type 1 variants to broadly neutralizing antibodies J Virol 2007 81 8533 8542 17522228 10.1128/JVI.02816-06 PMC1951377

[B117] Hall TA BioEdit: a user-friendly biological sequence alignment editor and analysis program for Windows 95/98/NT Nucl Acids Symp Ser 1999 41 95 98

[B118] Zhang M Gaschen B Blay W Foley B Haigwood N Kuiken C Korber B Tracking global patterns of N-linked glycosylation site variation in highly variable viral glycoproteins: HIV, SIV, and HCV envelopes and influenza hemagglutinin Glycobiology 2004 14 1229 1246 15175256 10.1093/glycob/cwh106

[B119] Posada D Crandall KA Modeltest: testing the model of DNA substitution Bioinformatics 1998 14 817 818 9918953 10.1093/bioinformatics/14.9.817

[B120] Wilgenbusch JC Swofford D Inferring evolutionary trees with PAUP* Curr Protoc Bioinformatics 2003 6.4.1 6.4.28 10.1002/0471250953.bi0604s00 18428704

[B121] Hasegawa M Kishino H Yano T Dating of the human-ape splitting by a molecular clock of mitochondrial DNA J Mol Evol 1985 22 160 174 3934395 10.1007/BF02101694

[B122] Lambert JS Mofenson LM Fletcher CV Moye J Jr Stiehm ER Meyer WA III Nemo GJ Mathieson BJ Hirsch G Sapan CV Safety and pharmacokinetics of hyperimmune anti-human immunodeficiency virus (HIV) immunoglobulin administered to HIV-infected pregnant women and their newborns. Pediatric AIDS Clinical Trials Group Protocol 185 Pharmacokinetic Study Group J Infect Dis 1997 175 283 291 9203648 10.1093/infdis/175.2.283

[B123] Tersmette M Winkel IN Groenink M Gruters RA Spence P Saman E van der Groen G Miedema F Huisman JG Detection and subtyping of HIV-1 isolates with a panel of characterized monoclonal antibodies to HIV-p24 gag Virology 1989 171 149 155 2472701 10.1016/0042-6822(89)90521-7

[B124] Edgar RC MUSCLE: multiple sequence alignment with high accuracy and high throughput Nucleic Acids Res 2004 32 1792 1797 15034147 10.1093/nar/gkh340 PMC390337

[B125] Heringa J Two strategies for sequence comparison: profile-preprocessed and secondary structure-induced multiple alignment Comput Chem 1999 23 341 364 10404624 10.1016/s0097-8485(99)00012-1

[B126] Larkin MA Blackshields G Brown NP Chenna R McGettigan PA McWilliam H Valentin F Wallace IM Wilm A Lopez R Clustal W and Clustal X version 2.0 Bioinformatics 2007 23 2947 2948 17846036 10.1093/bioinformatics/btm404

[B127] Kwong PD Wyatt R Robinson J Sweet RW Sodroski J Hendrickson WA Structure of an HIV gp120 envelope glycoprotein in complex with the CD4 receptor and a neutralizing human antibody Nature 1998 393 648 659 9641677 10.1038/31405 PMC5629912

[B128] Huang CC Tang M Zhang MY Majeed S Montabana E Stanfield RL Dimitrov DS Korber B Sodroski J Wilson IA Structure of a V3-containing HIV-1 gp120 core Science 2005 310 1025 1028 16284180 10.1126/science.1118398 PMC2408531

