# Supplementary figures and images for: HIV-1 envelope glycoprotein signatures that correlate with the development of cross-reactive neutralizing activity
Source: Retrovirology. 2013 Sep 23;10:102. doi: 10.1186/1742-4690-10-102 (PMC3849187; doi:10.1186/1742-4690-10-102)

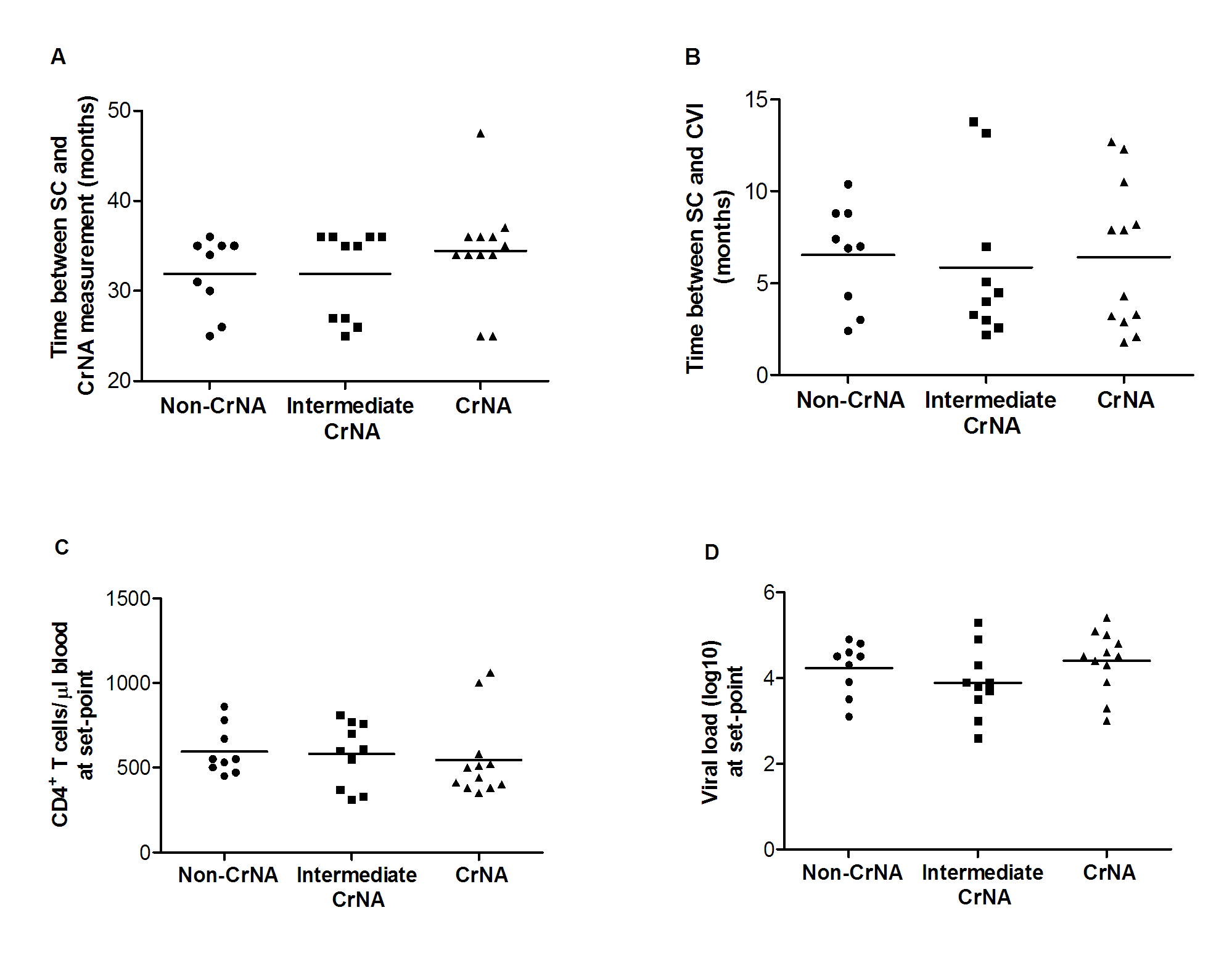

Supplement: Additional file 1: Figure S1 — Title of data: Baseline characteristics for the individuals with non-CrNA, intermediate CrNA and CrNA. Description of the data: (A) time, in months, between SC and CrNA level measured by Monogram Biosciences; (B) time, in months, between SC and isolation of clonal viral variants; (C) CD4+ T cells/μl blood at set-point; (D) viral load, in log10, at set-point. Individuals with non-CrNA, intermediate CrNA and CrNA in their serum are represented by circles, squares and triangles, respectively. Each individual is represented by one symbol. [file 1742-4690-10-102-S1.tiff]

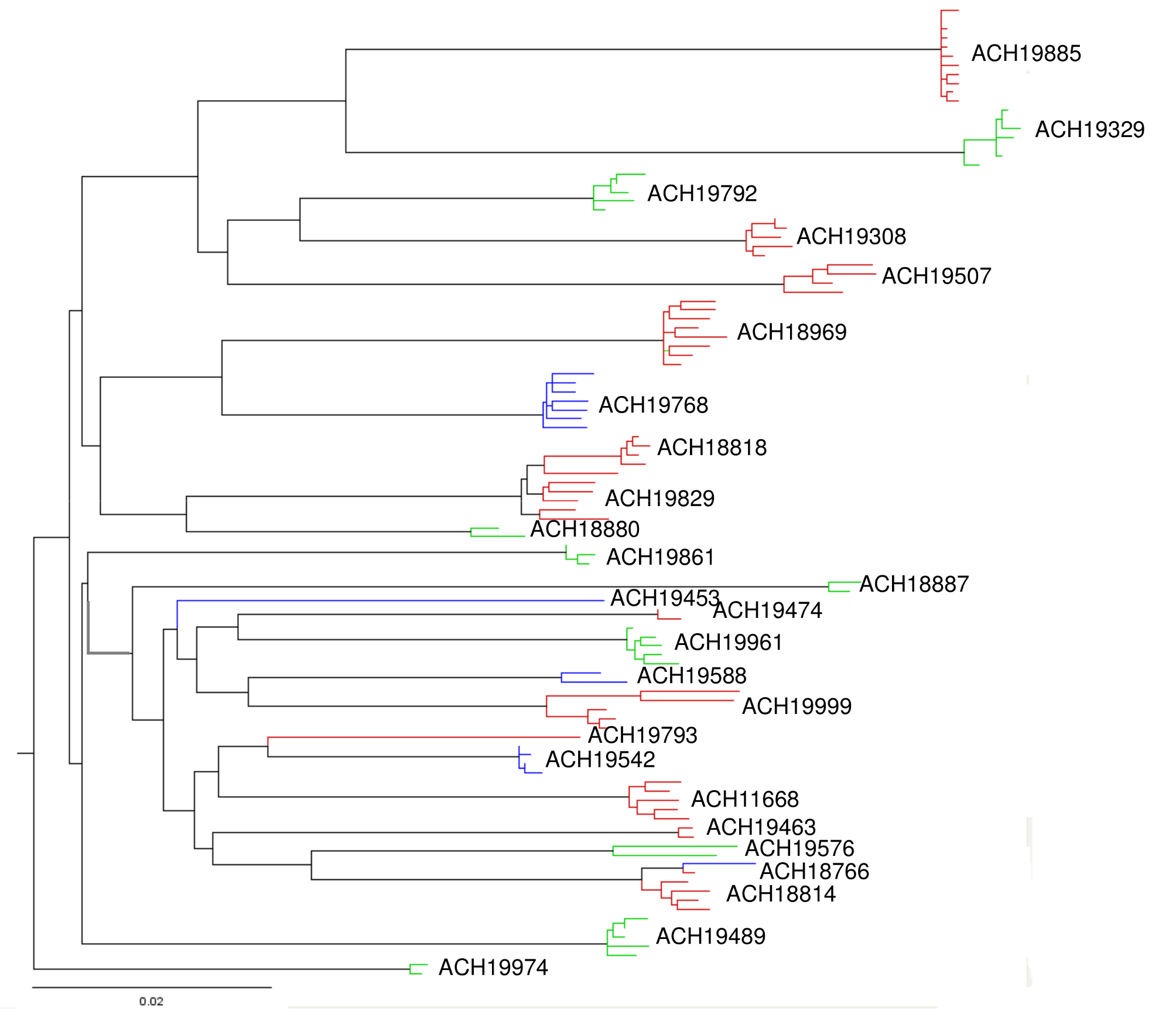

Supplement: Additional file 2: Figure S2 — Title of data: Genetic relationships between viruses from individuals with diverse levels of CrNA in serum. Description of data: Complete gp160 sequences derived from 26 individuals with varying levels of CrNA in serum were aligned and a ML tree was constructed. Individuals with high, intermediate and low CrNA are indicated in red, blue or green, respectively. [file 1742-4690-10-102-S2.tiff]

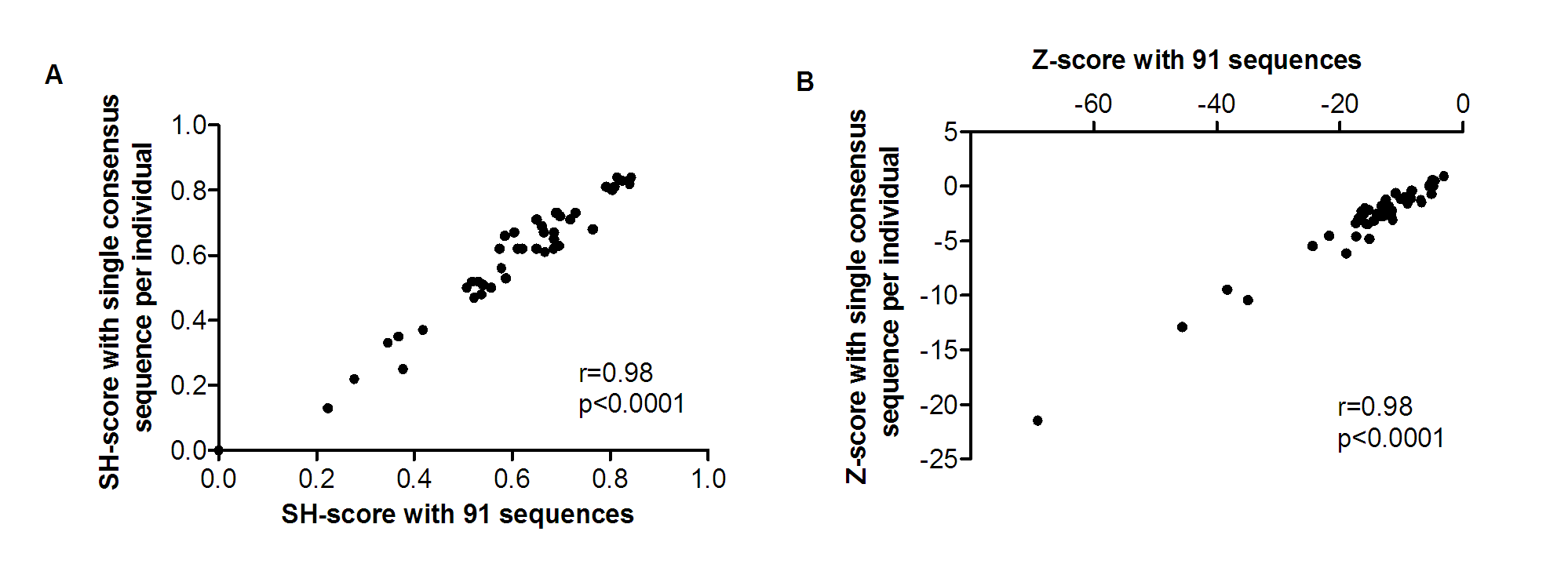

Supplement: Additional file 4: Figure S3 — Title of data: Multiple sequence alignment of gp160 sequences from CrNA and non- CrNA individuals used for SH analysis. Description of data: Multiple sequence alignment of 91 complete gp160 sequences from 21 individuals, starting at nucleotide position 91, excluding the Env signal peptide, with a minimum of one and a maximum of eleven sequences per individual. In total 58 sequences from twelve individuals who developed CrNA and 33 sequences from nine individuals that did not develop CrNA, CrNA and non-CrNA respectively, are depicted. [file 1742-4690-10-102-S4.tiff]
