# Supplementary material for: HIV-1 envelope glycoprotein signatures that correlate with the development of cross-reactive neutralizing activity
Source: Retrovirology. 2013 Sep 23;10:102. doi: 10.1186/1742-4690-10-102 (PMC3849187; doi:10.1186/1742-4690-10-102)
Supplement: Additional file 3: Table S1 — Title of data: Sequence Harmony results with consensus sequences. Description of data: The Sequence Harmony (SH) method ([90] and http://www.ibi.vu.nl/programs/seqharmwww) was used to analyze amino acid differences between the consensus env sequences of the twelve individuals who developed CrNA and the nine individuals who did not develop CrNA, for details see Methods. The SH algorithm is an entropy-based method, which detects positions within an alignment that display compositional differences in related protein sequences divided in two groups, and might therefore be linked to functional differences. In addition, an empirical Z-score is calculated, reflecting the significance of the SH-score obtained based on 100 random shuffling events of the sequences between the two groups. Cut-off scores were set as SH<0.7 for the residues in the variable regions and <0.85 for residues in the conserved regions, i.e. a less strict selection in the conserved region to allow also small(er) differences to be detected. The lower (negative) the z-score, the less likely that the results were found by chance. [file 1742-4690-10-102-S3.docx]

10 20 30 40 50 60 70 80

....|....|....|....|....|....|....|....|....|....|....|....|....|....|....|....|

**HXB2/1-856**  **MRVKEKYQHLWRWGWRWGTMLLGMLMICSATEKLWVTVYYGVPVWKEATTTLFCASDAKAYDTEVHNVWATHACVPTDPN**

**H18818.6.1D2_CrNA**  **..............................TEKLWVTVYYGVPVWKEATTTLFCASDAKAYDTEVHNVWATHACVPTDPS**

**H18818.6.1C3_CrNA**  **..............................TEKLWVTVYYGVPVWKEATTTLFCASDAKAYDTEVHNVWATHACVPTDPS**

**H18818.6.1A6_CrNA**  **..............................TEKLWVTVYYGVPVWKEATTTLFCASDAKAYDTEVHNVWATHACVPTDPS**

**H18818.6.1G12_CrNA**  **..............................TEKLWVTVYYGVPVWKEATTTLFCASDAKAYDTEVHNVWATHACVPTDPS**

**H19829.11.H5_CrNA**  **..............................TEKLWVTVYYGVPVWKEATTTLFCASDAKAYDTEVHNVWATHACVPTDPS**

**H19829.11.A2_CrNA**  **..............................TEKLWVTVYYGVPVWKEATTTLFCASDAKAYDTEVHNVWATHACVPTDPS**

**H19829.11.E8_CrNA**  **..............................TEKLWVTVYYGVPVWKEATTTLFCASDAKAYDTEVHNVWATHACVPTDPS**

**H19829.11.B4_CrNA**  **..............................TEKLWVTVYYGVPVWKEATTTLFCASDAKAYDTEVHNVWATHACVPTDPS**

**H19829.11.A4_CrNA**  **..............................TEKLWVTVYYGVPVWKEATTTLFCASDAKAYDTEVHNVWATHACVPTDPS**

**H19829.11.G8_CrNA**  **..............................TEKSWVTVYYGVPVWKEATTTLFCASDAKAYDTEVHNVWATHACVPTDPS**

**H19999.7.1G10_CrNA**  **..............................TEELWVTVYYGVPVWKEATTTLFCASDAKAYDTEVHNVWATHACVPTDPN**

**H19999.7.2G7_CrNA**  **..............................TENLWVTVYYGVPVWKEATTTLFCASDAKAYDTEVHNVWATHACVPTDPN**

**H19999.7.2D5_CrNA**  **..............................TENLWVTVYYGVPVWKEATTTLFCASDAKAYDTEVHNVWATHACVPTDPN**

**H19999.7.1B2_CrNA**  **.............................A.DKLWVTVYYGVPVWKEANTTLFCASDAKAYDTEVHNVWATHACVPTDPN**

**H19999.7.1D2_CrNA**  **..............................TDKLWVTVYYGVPVWKEANTSLFCASDAKAYDTEVHNVWATHACVPTDPN**

**H19507.18.G11_CrNA**  **..............................TDQLWVTVYYGVPVWKEATTTLFCASDAKAYDTEAHNVWATHACVPTDPN**

**H19507.18.C11_CrNA**  **..............................TDQLWVTVYYGVPVWKEATTTLFCASDAKAYDTEAHNVWATHACVPTDPN**

**H19507.18.A11_CrNA**  **..............................TDQLWVTVYYGVPVWKEATTTLFCASDAKAYDTEAHNVWAMHACVPTDPN**

**H19507.18.F4_CrNA**  **..............................TDQLWVTVYYGVPVWKEATTTLFCASDAKAYDTEAHNVWATHACVPTDPN**

**H19793.13.F8_CrNA**  **..............................TEKLWVTVYYGVPVWKEATTTLFCASDAKAYDTEVHNVWATHACVPTDPS**

**H19463.8.A11_CrNA**  **.............................V.EQLWVTVYYGVPVWKEAATTLFCASDAKAYDTEVHNVWATHACVPTDPN**

**H19463.8.E10_CrNA**  **.............................V.EQLWVTVYYGVPVWKEAATTLFCASDAKAYDTEVHNVWATHACVPTDPN**

**H19474.17.1G12_CrNA**  **..............................TEELWVTVYYGVPVWKEATTILFCASDAKAYDTEVHNVWATHACVPTDPN**

**H19474.17.2H8_CrNA**  **..............................TEELWVTVYYGVPVWKEATTILFCASDAKAYDTEVHNVWATHACVPTDPN**

**H18814.10.1E1_CrNA**  **..............................TDQKWVTVYYGVPVWKEATTTLFCASDAKAYDTEVHNVWATHACVPTDPN**

**H18814.10.1B4_CrNA**  **..............................TDQKWVTVYYGVPVWKEATTTLFCASDAKAYDTEVHNVWATHACVPTDPN**

**H18814.10.1C5_CrNA**  **..............................TDQKWVTVYYGVPVWKEATTTLFCASDAKAYDTEVHNVWATHACVPTDPN**

**H18814.10.1B1_CrNA**  **..............................TDQKWVTVYYGVPVWKEATTTLFCASDAKAYDTEVHNIWATHACVPTDPN**

**H18814.10.1G2_CrNA**  **..............................TDQKWVTVYYGVPVWKEATTTLFCASDAKAYDTEVHNVWATHACVPTDPN**

**H11668.12.F11(A)_CrNA**  **.............................V.GQLWVTVYYGVPVWKEATTTLFCASDAKAYATEVHNVWATHACVATDPN**

**H11668.12.C3_CrNA**  **.............................V.GQLWVTVYYGVPVWKEATTTLFCASDAKAYTTEVHNVWATHACVPTDPN**

**H11668.12.D11_CrNA**  **.............................V.GQLWVTVYYGVPVWKEATTTLFCASDAKAYATEVHNVWATHACVPTDPN**

**H11668.12.H9_CrNA**  **.............................V.GQLWVTVYYGVPVWKEATTTLFCASDAKAYATEVHNVWATHACVPTDPN**

**H11668.12.E10_CrNA**  **.............................V.GQLWVTVYYGVPVWKEATTTLFCASDAKAYATEVHNVWATHACVPTDPN**

**H19308.26.B1_CrNA**  **.............................A.EQLWVTVYYGVPVWKEATTTLFCASDAKAYDTEAHNVWATHACVPTDPN**

**H19308.26.F8I_CrNA**  **.............................A.EQLWVTVYYGVPVWKEATTTLFCASDAKAYDTEAHNVWATHACVPTDPN**

**H19308.26.D8_CrNA**  **.............................A.EQLWVTVYYGVPVWKEATTTLFCASDAKAYDTEAHNVWATHACVPTDPN**

**H19308.26.D1_CrNA**  **.............................A.EQLWVTVYYGVPVWKEATTTLFCASDAKAYDTEAHNVWATHACVPTDPN**

**H19308.26.E4_CrNA**  **.............................A.EQLWVTVYYGVPVWKEATTTLFCASDAKAYDTEAHNVWATHACVPTDPN**

**H19885.31.D9_CrNA**  **.............................A.E.LWVTVYYGVPVWKEATTTLFCASDAKSYDTEVHNVWATHACVPTDPN**

**H19885.31.G12_CrNA**  **.............................A.E.LWVTVYYGVPVWKEATTTLFCASDAKSYDTEVHNVWATHACVPTDPN**

**H19885.31.E2_CrNA**  **.............................A.E.LWVTVYYGVPVWKEATTTLFCASDAKSYDTEVHNVWATHACVPTDPN**

**H19885.31.G10_CrNA**  **.............................A.E.LWVTVYYGVPVWKEATTTLFCASDAKSYDTEVHNVWATHACVPTDPN**

**H19885.31.H11_CrNA**  **.............................A.E.LWVTVYYGVPVWKETTTTLFCASDAKSYDTEVHNVWATHACVPTDPN**

**H19885.31.A5_CrNA**  **.............................A.E.LWVTVYYGVPVWKEATTTLFCASDAKSYDTEVHNVWATHACVPIDPN**

**H19885.31.F8_CrNA**  **.............................A.E.LWVTVYYGVPVWKEATTTLFCASDAKSYDTEVHNVWATHACVPTDPN**

**H19885.31.H10_CrNA**  **.............................A.E.LWVTVYYGVPVWKEATTTLFCASDAKSYDTEVHNVWATHACVPTDPN**

**H19885.31.G1_CrNA**  **.............................A.E.LWVTVYYGVPVWKEATTTLFCASDAKSYDTEVHNVWATHACVPTDPN**

**H19885.31.C6_CrNA**  **.............................A.E.LWVTVYYGVPVWKEATTTLFCASDAKSYDTEVHNVWATHACVPIDPN**

**H19885.31.E11_CrNA**  **.............................A.E.LWVTVYYGVPVWKEATTTLFCASDAKSYDTEVHNVWATHACVPTDPN**

**H18969.12.9D9_CrNA**  **.............................A.DQLWVTVYYGVPVWKDTTTTLFCASDAKAYDTEVHNVWATHACVPTDPN**

**H18969.12.8E6_CrNA**  **.............................A.DQLWVTVYYGVPVWKDTTTTLFCASDAKAYDTEVHNVWATHACVPTDPN**

**H18969.12.8G8_CrNA**  **.............................A.DQLWVTVYYGVPVWKDTTTTLFCASDAKAYDTEVHNVWATHACVPTDPN**

**H18969.12.6D7_CrNA**  **.............................A.DQLWVTVYYGVPVWKDTTTTLFCASDAKAYDTEVHNVWATHACVPTDPN**

**H18969.12.8B4_CrNA**  **.............................A.DQLWVTVYYGVPVWKDTTTTLFCASDAKAYDTEVHNVWATHACVPTDPN**

**H18969.12.7D5_CrNA**  **.............................A.DQLWVTVYYGVPVWKDTTTTLFCASDAKAYDTEVHNVWATHACVPTDPN**

**H18969.12.6C4_CrNA**  **.............................A.DQLWVTVYYGVPVWKDTTTTLFCASDAKAYDTEVHNVWATHACVPTDPN**

**H18969.12.10H3_CrNA**  **.............................A.DQLWVTVYYGVPVWKDTTTTLFCASDAKAYDTEVHNVWATHACVPTDPN**

**H19329.32.C9_Non-CrNA**  **..............................TGNLWVTVYYGVPVWKEATTTLFCASDAKAYETEVHNVWATHACVPTDPN**

**H19329.32.E6_Non-CrNA**  **..............................TGNLWVTVYYGVPVWKEATTTLFCASDAKAYETEVHNVWATHACVPTDPN**

**H19329.32.H7_Non-CrNA**  **..............................TGNLWVTVYYGVPVWKEPPTTLFCASDAKAYETEVHNVWATHACVPTDPN**

**H19329.32.F1_Non-CrNA**  **..............................TGNLWVTVYYGVPVWKEATTTLFCASDAKAYETEVHNVWATHACVPTDPN**

**H19329.32.H5_Non-CrNA**  **..............................TGNLWVTVYYGVPVWKEATTTLFCASDAKAYETEVHNVWATHACVPTDPN**

**H19329.32.H9_Non-CrNA**  **..............................TGNLWVTVYYGVPVWKEATTTLFCASDAKAYETEVHNVWATHACVPTDPN**

**H19329.13.F12_Non-CrNA**  **..............................TGNLWVTVYYGVPVWKEATTTLFCASDAKAYETEVHNVWATHACVPTDPN**

**H18887.21.G2_Non-CrNA**  **.............................A.NNLWVTVYYGVPVWKEATTTLFCASDAKAYDTEVHNVWATHACVPTDPN**

**H18887.21.roD7_Non-CrNA** **.............................A.NNLWVTVYYGVPVWKEATTTLFCASDAKAYDTEVHNVWATHACVPTDPN**

**H19861.19.C10_Non-CrNA**  **..............................TDQLWVTVYYGVPVWKEATTTLFCASDAKAYDTDVHNVWATHACVPTDPN**

**H19861.19.F2_Non-CrNA**  **..............................TDQLWVTVYYGVPVWKEATTTLFCASDAKAYDTDVHNVWATHACVPTDPN**

**H19861.19.A6_Non-CrNA**  **..............................TDQLWVTVYYGVPVWKEATTTLFCASDAKAYDTEVHNVWATHACVPTDPN**

**H19489.8.G5_Non-CrNA**  **..............................TEKLWVTVYYGVPVWKEATTTLFCASDAKAYDTEVHNVWATHACVPTDPN**

**H19489.8.1E10_Non-CrNA**  **..............................TEKLWVTVYYGVPVWKEATTTLFCASDAKAYDTEVHNVWATHACVPTDPN**

**H19489.8.1A11_Non-CrNA**  **..............................TEKLWVTVYYGVPVWKEATTTLFCASDAKAYDTEVHNVWATHACVPTDPN**

**H19489.8.1H10_Non-CrNA**  **..............................TEKLWVTVYYGVPVWKEATTTLFCASDAKAYDTEVHNVWATHACVPTDPN**

**H19489.8.2A3_Non-CrNA**  **..............................TEKLWVTVYYGVPVWKEATTTLFCASDAKAYDTEVHNVWATHACVPTDPN**

**H19974.11.E12_Non-CrNA**  **.............................AEEELWVTVYYGVPVWKEATTTLFCASDAKAYDTEVHNVWATHACVPTDPS**

**H19974.11.E11_Non-CrNA**  **.............................AEEELWVTVYYGVPVWKEATTTLFCASDAKAYDTEVHNVWATHACVPTDPS**

**H19792.9.F6_Non-CrNA**  **.............................A.KNLWVTVYYGVPVWKEANTTLFCASDAKAYDTEVHNVWATHACVPTDPN**

**H19792.9.B1_Non-CrNA**  **.............................A.KNLWVTVYYGVPVWKEANTTLFCASDAKAYDTEVHNVWATHACVPTDPN**

**H19792.9.F1_Non-CrNA**  **.............................A.KNLWVTVYYGVPVWKEANTTLFCASDAKAYDTEVHNAWATHACVPTDPN**

**H19792.9.D6_Non-CrNA**  **.............................A.KDLWVTVYYGVPVWKDANTTLFCASDAKAYDTEVHNVWATHACVPTDPN**

**H19792.9.C10_Non-CrNA**  **.............................A.KNLWVTVYYGVPVWKDANTTLFCASDAKAYDTEVHNVWATHACVPTDPN**

**H18880.10.20_Non-CrNA**  **..............................TEQLWVTVYYGVPVWKEATTTLFCASDAKAYDTQVHNVWATHACVPTDPN**

**H18880.10.21_Non-CrNA**  **..............................TEQLWVTVYYGVPVWKEATTTLFCASDAKAYDTEVHNVWATHACVPTDPN**

**H19961.14.F10_Non-CrNA**  **.............................A.GNLWVTVYYGVPVWKEATTTLFCASDARAYDTEAHNVWATHACVPTDPN**

**H19961.14.E8_Non-CrNA**  **.............................A.GNLWVTVYYGVPVWKEATTTLFCASDARAYETEAHNVWATHACVPTDPN**

**H19961.14.G4_Non-CrNA**  **.............................A.GNLWVTVYYGVPVWKEATTTLFCASDARAYGTEAHNVWATHACVPTDPN**

**H19961.14.B10_Non-CrNA**  **.............................A.GNLWVTVYYGVPVWKEATTTLFCASDARAYGTEAHNVWATHACVPTDPN**

**H19961.14.F9_Non-CrNA**  **.............................A.GNLWVTVYYGVPVWKEATTTLFCASDARAYETEAHNVWATHACVPTDPN**

**H19576.9.H1_Non-CrNA**  **.............................A.EQLWVTVYYGVPVWKEATTTLFCASDAKAYDTEVHNVWATHACVPTDPN**

**H19576.9.F4_Non-CrNA**  **.............................A.EQLWVTVYYGVPVWKEATTTLFCASDAKAYDTEVHNVWATHACVPTDPN**

90 100 110 120 130 140 150 160

....|....|....|....|....|....|....|....|....|....|....|....|....|....|....|....|

**HXB2/1-856**  **PQEVVLVNVTENFNMWKNDMVEQMHEDIISLWDQSLKPCVKLTPLCVSLKCT.D.L.KND..TNT...............**

**H18818.6.1D2_CrNA**  **PQEVILENVTENFNMWTNNMVEQMHEDIISLWDQSLKPCVKLTPLCVTLNCTNE.L.KN...TTKT..............**

**H18818.6.1C3_CrNA**  **PQEVILENVTENFNMWTNNMVEQMHEDIISLWDQSLKPCVKLTPLCVTLNCTNE.L.KN...TTKT..............**

**H18818.6.1A6_CrNA**  **PQEVILENVTENFNMWTNNMVEQMHEDIISLWDQSLKPCVKLTPLCVTLNCTNE.L.KN...TTKT..............**

**H18818.6.1G12_CrNA**  **PQEVILENVTENFNMWTNNMVEQMHEDIISLWDQSLKPCVKLTPLCVTLNCTNE.L.KN...TTKT..............**

**H19829.11.H5_CrNA**  **PQEVILENVTENFNMWTNNMVEQMHEDIISLWDQSLKPCVKLTPLCVTLNCTNE.L.KN...TTTT..............**

**H19829.11.A2_CrNA**  **PQEVILENVTENFNMWTNNMVEQMHEDIISLWDQSLKPCVKLTPLCVTLNCTNE.L.KN...TTTT..............**

**H19829.11.E8_CrNA**  **PQEVILENVTENFNMWTNNMVEQMHEDIISLWDQSLKPCVKLTPLCVTLNCTNE.L.KN...TTTT..............**

**H19829.11.B4_CrNA**  **PQEVILENVTENFNMWTNNMVEQMHEDIISLWDQSLKPCVKLTPLCVTLNCTNE.L.KN...TTTT..............**

**H19829.11.A4_CrNA**  **PQEVILENVTENFNMWTNNMVEQMHEDIISLWDQSLKPCVRLTPLCVTLNCTNE.L.KN...TTNT..............**

**H19829.11.G8_CrNA**  **PQEVILENVTENFNMWTNNMVEQMHEDIISLWDQSLKPCVKLTPLCVTLNCTNE.L.KN.....TTET............**

**H19999.7.1G10_CrNA**  **PQEVELENVTENFNMWKNNMVEQMHEDIISLWDQSLKPCVKLTPLCVTLNCT.D.L.RN..AT.................**

**H19999.7.2G7_CrNA**  **PQEVELENVTENFNMWKNNMVEQMHEDIISLWDQSLKPCVKLTPLCVTLNCT.D.L.RN..AT.................**

**H19999.7.2D5_CrNA**  **PQEVELENVTENFNMWKNNMVEQMHEDIISLWDQSLKPCVKLTPLCVTLNCT.D.L.RN..AT.................**

**H19999.7.1B2_CrNA**  **PQEVKLENVTENFNMWKNNMVEQMHEDIISLWDQSLKPCVKLTPLCVTLNCT.D.L.RN..ATNNSTT............**

**H19999.7.1D2_CrNA**  **PQEVKLENVTENFNMWKNNMVEQMHEDIISLWDQSLKPCVKLTPLCVTLNCT.D.L.RND..TNNST.I...........**

**H19507.18.G11_CrNA**  **PQEVVLGNVTENFNMWKNNMVEQMHEDIISLWEESLKPCVKLTPLCVTLNCT.N.L.KN..ATNTNETTH..........**

**H19507.18.C11_CrNA**  **PQEVVLGNVTENFNMWKNNMVEQMHEDIISLWEESLKPCVKLTPLCVTLNCT.N.L.KN..ATNTNETTH..........**

**H19507.18.A11_CrNA**  **PQEVVLGNVTENFNMWKNNMVEQMHEDIISLWEESLKPCVKLTPLCVTLNCT.N.L.KNE...TTP..............**

**H19507.18.F4_CrNA**  **PQEVVLGNVTENFNMWKNNMVEQMHEDIISLWEESLKPCVKLTPLCVTLNCT.N.L.KNE...TTP..............**

**H19793.13.F8_CrNA**  **PQEVVLENVTENFNMWKNNMVEQMHEDIISLWDQSLKPCVELTPLCVTLNCT.D.L.RN..ATNT...............**

**H19463.8.A11_CrNA**  **PQEVVLENVTENFNMWNNNMVEQMHEDIISLWDQSLKPCVKLTPLCVTLECY.D.LNRT...NTNT..............**

**H19463.8.E10_CrNA**  **PQEVVLENVTENFNMWNNNMVEQMHEDIISFWDQSLKPCVKLTPLCVTLECY.D.LNRT...NTNT..............**

**H19474.17.1G12_CrNA**  **PQEVVLGNVTENFNVWKNNMVEQMQEDIISLWDQSLKPCVKLTPLCVTLNCTDD.L.KN...TNN...............**

**H19474.17.2H8_CrNA**  **PQEVVLGNVTENFNVWKNNMVKQMQEDIISLWDQSLKPCVKLTPLCVTLNCTDD.L.KN...TNN...............**

**H18814.10.1E1_CrNA**  **PQEIELKNVTENFNMWKNDMVEQMHEDIISLWDQSLKPCVKLTPLCVTLNCT.D.F.GNT..TNT...............**

**H18814.10.1B4_CrNA**  **PQEIELKNVTENFNMWKNDMVEQMHEDIVSLWDQSLKPCVKLTPLCVTLNCT.D.F.GNT..TNT...............**

**H18814.10.1C5_CrNA**  **PQEIELKNVTENFNMWKNDMVEQMHEDIISLWDQSLKPCVKLTPLCVTLNCT.D.F.GNT..TNT...............**

**H18814.10.1B1_CrNA**  **PQEIELKNVTENFNMWKNDMVEQMHEDIISLWDQSLKPCVKLTPLCVTLNCT.D.F.GNT..TNT...............**

**H18814.10.1G2_CrNA**  **PQEIELKNVTENFNMWKNDMVEQMHEDIISLWDQSLKPCVKLTPLCVTLNCT.D.F.GNT..TNT...............**

**H11668.12.F11(A)_CrNA**  **PQEVVLENVTENFNMWKNNMVEQMHEDIISLWDQSLKPCVKLTPLCVTLNCS.D.L.GNN..TNSGNNK...........**

**H11668.12.C3_CrNA**  **PQEVVLENVTENFNMWKNNMVEQMHEDIISLWDQSLKPCVKLTPLCVTLNCT.N.L.GND..TNSGNNN...........**

**H11668.12.D11_CrNA**  **PQEVVLGNVTENFNMWKNNMVEQMHEDIISLWDQSLKPCVKLTPLCVTLNCT.D.L.RND..TNSGNNN...........**

**H11668.12.H9_CrNA**  **PQEVVLENVTENFNMWKNNMVEQMHEDIISLWDQSLKPCVKLTPLCVTLNCS.D.L.RND..TNSGNNN...........**

**H11668.12.E10_CrNA**  **PQEVVLENVTENFNMWKNNMVEQMHEDIISLWDQSLKPCVKLTPLCVTLNCS.N.L.RND..TNSGNNN...........**

**H19308.26.B1_CrNA**  **PQEVVLGNVTENFNMWKNNMVEQMHEDIISLWDQSLKPCVKLTPLCVTLNCT.N.L.QN..ATNTN..............**

**H19308.26.F8I_CrNA**  **PQEVVLGNVTENFNMWKNNMVEQMHEDIISLWDQSLKPCVKLTPLCVTLNCT.N.L.QN..ATNTN..............**

**H19308.26.D8_CrNA**  **PQEVVLGNVTENFNMWKNNMVEQMHEDIISLWDQSLKPCVKLTPLCVTLNCT.N.L.QN..VTN................**

**H19308.26.D1_CrNA**  **PQEVVLGNVTENFNMWKNNMVEQMHEDIISLWDQSLKPCVKLTPLCVTLNCT.N.L.QN..ATNTN..............**

**H19308.26.E4_CrNA**  **PQEVVLGNVTENFNMWKNNMVEQMHEDIISLWDQSLKPCVKLTPLCVTLNCT.N.L.QN..VTNINTN............**

**H19885.31.D9_CrNA**  **PQEVVLENVTENFNMWKNNMVEQMHEDIISLWDQSLKPCVKLTPLCVTLNCT.D.L.KN..ATNTNSTI...........**

**H19885.31.G12_CrNA**  **PQEVVLENVTENFNMWKNNMVEQMHEDIISLWDQSLKPCVKLTPLCVTLNCT.D.L.KN..ATNTNSTI...........**

**H19885.31.E2_CrNA**  **PQEVVLENVTENFNMWKNNMVEQMHEDIISLWDQSLKPCVKLTPLCVTLNCT.D.L.KN..ATNTNSTI...........**

**H19885.31.G10_CrNA**  **PQEVVLENVTENFNMWKNNMVEQMHEDIISLWDQSLKPCVKLTPLCVTLNCT.D.L.KN..ATNTNSTI...........**

**H19885.31.H11_CrNA**  **PQEVVLENVTENFNMWKNNMVEQMHEDIISLWDQSLKPCVKLTPLCVTLNCT.D.L.KN..ATNTNSTI...........**

**H19885.31.A5_CrNA**  **PQEVVLENVTENFNMWKNNMVEQMHEDIISLWDQSLKPCVKLTPLCVTLNCT.D.L.KN..ATNTNSTI...........**

**H19885.31.F8_CrNA**  **PQEVVLENVTENFNMWINNMVEQMHEDIISLWDQSLKPCVKLTPLCVTLNCT.D.L.KN..ATNTNSTI...........**

**H19885.31.H10_CrNA**  **PQEVVLENVTENFNMWKNNMVEQMHEDIISLWDQSLKPCVKLTPLCVTLNCT.D.L.KN..ATNTNSTI...........**

**H19885.31.G1_CrNA**  **PQEVVLENVTENFNMWKNNMVEQMHEDIISLWDQSLKPCVKLTPLCVTLNCT.D.L.KN..ATNTNSTI...........**

**H19885.31.C6_CrNA**  **PQEVVLENVTENFNMWKNNMVEQMHEDIISLWDQSLKPCVKLTPLCVTLNCT.D.L.KN..ATNTNSTI...........**

**H19885.31.E11_CrNA**  **PQEVVLENVTENFNMWKNNMVEQMHEDIISLWDQSLKPCVKLTPLCVTLNCT.D.L.KN..ATNTNSTI...........**

**H18969.12.9D9_CrNA**  **PQEIALENVTEDFNMWKNNMVEQMHEDIISLWDQSLKPCVKLTPLCVTLNCT.E.L.ENT.I.....NI...........**

**H18969.12.8E6_CrNA**  **PQEIALENVIEDFNMWKNNMVEQMHEDIISLWDQSLKPCVKLTPLCVTLNCT.E.L.ENT.I.....NI...........**

**H18969.12.8G8_CrNA**  **PQEIALENVTEDFNMWKNNMVEQMHEDIISLWDQSLKPCVKLTPLCVTLNCT.E.L.ENTTI.....NI...........**

**H18969.12.6D7_CrNA**  **PQEIALENVTEDFNMWKNNMVEQMHEDIISLWDQSLKPCVKLTPLCVTLNCT.E.L.ENT.I.....NI...........**

**H18969.12.8B4_CrNA**  **PQEIALENVTEDFNMWKNNMIEQMHEDIISLWDQSLKPCVKLTPLCVTLNCT.E.L.ENT.I.....NS...........**

**H18969.12.7D5_CrNA**  **PQEIALENVTEDFNMWKNNMVEQMHEDIISLWDQSLKPCVKLTPLCVTLNCT.E.L.ENT.I.....NI...........**

**H18969.12.6C4_CrNA**  **PQEIALENVTEDFNMWKNNMVEQMHEDIISLWDQSLKPCVKLTPLCVTLNCT.E.L.ENT.I.....NI...........**

**H18969.12.10H3_CrNA**  **PQEIALENVTEDFNMWKNNMVEQMHEDIISLWDQSLKPCVKLTPLCVTLNCT.E.L.ENT.I.....NI...........**

**H19329.32.C9_Non-CrNA**  **PQELVLENVTENFNMWKNNMVEQMHEDIISLWDESLKPCVKLTPLCVTLNCT.N.V..N..I.TSSNNITS.........**

**H19329.32.E6_Non-CrNA**  **PQEPVLENVTENFNMWKNNMVEQMHEDIISLWDESLKPCVKLTPLCVTLNCT.N.A..N..I.TSSNNITS.........**

**H19329.32.H7_Non-CrNA**  **PQELVLENVTENFNMWKNNMVEQMHEDIISLWDESLKPCVKLTPLCVTLNCT.N.V..N..I.TSSNNITS.........**

**H19329.32.F1_Non-CrNA**  **PQELVLENVTENFNMWKNNMVEQMHEDIISLWDESLKPCVKLTPLCVTLNCT.N.V..N..I.TSSNNITS.........**

**H19329.32.H5_Non-CrNA**  **PQELVLENVTENFNMWKNNMVEQMHEDIISLWDESLKPCVKLTPLCVTLNCT.N.V..N..I.TSSNNITS.........**

**H19329.32.H9_Non-CrNA**  **PQELVLENVTENFNMWKNNMVEQMHEDIISLWDESLKPCVKLTPLCVTLNCT.N.V..N..I.TSSNNITS.........**

**H19329.13.F12_Non-CrNA**  **PQELVLENVTENFNMWKNNMVEQMHEDIISLWDESLKPCVKLTPLCVTLNCT.N.V..N..I.TSSNNITS.........**

**H18887.21.G2_Non-CrNA**  **PQEVRLDNVTENFNMWKNNMVEQMHEDIISLWDQSLKPCVKLTPLCVTLDCT.D.L.KNTTNTTSSQNATN.........**

**H18887.21.roD7_Non-CrNA** **PQEVRLDNVTENFNMWKNNMVEQMHEDIISLWDQSLKPCVKLTPLCVTLDCT.D.L.KNTTNTTSSQNATN.........**

**H19861.19.C10_Non-CrNA**  **PQEVVLGNVTENFNMWKNNMVEQMHEDIISLWDQSLKPCVKLTPLCVTLNCT.N.L...........NATN.........**

**H19861.19.F2_Non-CrNA**  **PQEVVLGNVTENFNMWKNNMVEQMHEDIISLWDQSLKPCVKLTPLCVTLNCT.N.L...........NATN.........**

**H19861.19.A6_Non-CrNA**  **PQEVVLGNVTENFNMWKNNMVEQMHEDIISLWDQSLKPCVKLTPLCVTLNCT.N.L...........NATN.........**

**H19489.8.G5_Non-CrNA**  **PQEVVLENVTENFNMWKNDMVEQMHEDIISLWDQSLKPCVELTPLCVTLDCT.DYV.GN..VTNA..NATN.........**

**H19489.8.1E10_Non-CrNA**  **PQEVVLENVTENFNMWNNDMVEQMHEDIISLWDQSLKPCVELTPLCVTLDCT.DYV.GN..ATNA..NATNTTSGSSRGT**

**H19489.8.1A11_Non-CrNA**  **PQEVVLENVTENFNMWKNDMVEQMHEDIISLWDQSLKPCVELTPLCVTLDCT.D...H...VG....NATN.....AK..**

**H19489.8.1H10_Non-CrNA**  **PQEVVLENVTENFNMWKNDMVEQMHEDIISLWDQSLKPCVELTPLCVTLDCT.DYV.GN..VTNA..NATN.........**

**H19489.8.2A3_Non-CrNA**  **PQEVVLENVTENFNMWKNDMVEQMHEDIISLWDQSLKPCVELTPLCVTLDCT.D.A...........NATN.........**

**H19974.11.E12_Non-CrNA**  **PQEVVLANVTENFNMWKNNMVEQMHEDIVSLWDQSLKPCVKLTPLCVTLNCT.D.L.GNATDAINR.NVTD.........**

**H19974.11.E11_Non-CrNA**  **PQEVVLANVTENFNMWKNNMVEQMHEDIISLWDQSLKPCVKLTPLCVTLNCT.D.L.GNATDAINR.NTTD.........**

**H19792.9.F6_Non-CrNA**  **PQEVVLENVTENFNMWKNNMVEQMHEDIISLWDQSLKPCVKLTPLCVTLNCT.N.V.NTNNSSSL.NNTTN.........**

**H19792.9.B1_Non-CrNA**  **PQEVVLENVTENFNMWKNNMVEQMHEDIISLWDQSLKPCVKLTPLCVTLNCT.N.V.NTNNSSSL.NNTTN.........**

**H19792.9.F1_Non-CrNA**  **PQEVVLENVTENFNMWKNNMVEQMHEDIISLWDQSLKPCVKLTPLCVTLNCT.N.V.NTNNSSSL.NNTTN.........**

**H19792.9.D6_Non-CrNA**  **PQEVVLENVTENFNMWKNNMVEQMHEDIISLWDQSLKPCVKLTPLCVTLNCT.N.V.NTNNSSSL.NNTTN.........**

**H19792.9.C10_Non-CrNA**  **PQEVVLENVTENFNMWKNNMVEQMHEDIISLWDQSLKPCVKLTPLCVTLNCT.N.V.NTNNSSSL.NNTTN.........**

**H18880.10.20_Non-CrNA**  **PQEVVLENVTENFNMWKNNMVEQMHEDIISLWDQSLKPCVKLTPLCVTLNCT.D.L.........R.NATN.........**

**H18880.10.21_Non-CrNA**  **PQEVVLENVTENFNMWKNNMVQQMHEDIISLWDQSLKPCVKLTPLCVTLNCT.D.L.........R.NATN.........**

**H19961.14.F10_Non-CrNA**  **PQEVVLENVTENFNMWRNNMVEQMHEDIISLWDQSLKPCVKLTPLCVTLNCS.D.M.........G.NETA.........**

**H19961.14.E8_Non-CrNA**  **PQEVVLENVTENFNMWRNNMVEQMHEDIISLWDQSLKPCVKLTPLCVTLNCS.D.M.GN......E.NTTN.........**

**H19961.14.G4_Non-CrNA**  **PQEVVLENVTENFNMWRNNMVEQMHEDIISLWDQSLKPCVKLTPLCVTLNCS.D.M.........G.NETA.........**

**H19961.14.B10_Non-CrNA**  **PQEVVLENVTENFNMWRNNMVEQMHEDIISLWDQSLKPCVKLTPLCVTLNCS.D.M.........G.NETA.........**

**H19961.14.F9_Non-CrNA**  **PQEVVLENVTENFNMWRNNMVEQMHEDIISLWDQSLKPCVKLTPLCVTLNCS.D.M.........G.NETA.........**

**H19576.9.H1_Non-CrNA**  **PQEVELGNVTEKFNMWKNNMVEQMHEDIISLWDQSLKPCVKLTPLCVTLNCN.NSIDW.......K.NTTS.........**

**H19576.9.F4_Non-CrNA**  **PQEVELGNVTENFNMWKNNMVEQMHEDIISLWDQSLKPCVKLTPLCVTLNCN.NSINW.......K.NVTN.........**

170 180 190 200 210 220 230 240

....|....|....|....|....|....|....|....|....|....|....|....|....|....|....|....|

**HXB2/1-856**  **.NSSS.G..RMIM.EK...GEIKNCSFNISTSIRGKVQKEYAFFYKLDIIPIDND......TTS......YKLTSCNTSV**

**H18818.6.1D2_CrNA**  **.NNSSWG............GEMKNCSFNVTTSIRDKVQKEYALFYKLDIVPIDDD.....NNTSN.....YRLINCNTSV**

**H18818.6.1C3_CrNA**  **.NNSSWG............GEMKNCSFKVTTSIRDKVQKEYALFYKLDIVPIDDD.....NNTSN.....YRLINCNTSV**

**H18818.6.1A6_CrNA**  **.NNSSWG............GEMKNCSFNVTTSIRDKVQKEYALFYKLDIVPIDDD.....NNTSNYTSN.YRLINCNTSV**

**H18818.6.1G12_CrNA**  **.NNSSWG............GEMKNCSFNVTTSIRDKVQKEYALFYKLDIVPIDDD.....NNTSNYTSN.YRLINCNTSV**

**H19829.11.H5_CrNA**  **.NNSSWG............GEMKNCSFNITTSIRDKVQKEYALFYKLDIVPIDDD.....NNTSNYNTSNYRLINCNTSV**

**H19829.11.A2_CrNA**  **.NNSSWG............GEMKNCSFNITTSIRDKVQKEYALFYKLDIVPIDDD.....NNTSNYNTSNYRLINCNTSV**

**H19829.11.E8_CrNA**  **.NNSSWG............GEMKNCSFNITTSIRDKVQKEYALFYKLDIVPIDDD.....NNTSNYNTSNYRLINCNTSV**

**H19829.11.B4_CrNA**  **.NNSSWG............GEMKNCSFNITTSIRDKVQKEYALFYKLDIVPIGDD.....NNTSNYNTSNYRLINCNTSV**

**H19829.11.A4_CrNA**  **.NNSSWG............GEMKNCSFNITTSIRDKVQKEYALFYKLDIVPIDDD.....NNTSNYNTSNYRLINCNTSV**

**H19829.11.G8_CrNA**  **.NNSSWG............GEMKNCSFNITTSIRDKVQKEYALFYKLDIVPIGDD.....NNTSNYNTSNYRLINCNTSV**

**H19999.7.1G10_CrNA**  **.NNSS..M.RM.M.ER...GEIKNCSFNITTSIRDKMQKEYALLYKLDIVPIDND......NTS......YRLISCNTSV**

**H19999.7.2G7_CrNA**  **.NNSS..M.RM.M.ER...GEIKNCSFNITTSIRDKMQKEYALLYKLDIVPIDND......NTS......YRLISCNTSV**

**H19999.7.2D5_CrNA**  **.NNSS..M.RM.M.ER...GEIKNCSFNITTSIRDKMQKEYALLYKLDIVPIDND......NTS......YRLISCNTSV**

**H19999.7.1B2_CrNA**  **.SNSS..M.E..M.EG...GEIKNCSFNITTSIRDKMQKEYALLYKLDIVPIDNNSTR............YRLISCNTSV**

**H19999.7.1D2_CrNA**  **.NNSS..M.K..M.ET...GEIKNCSFNITTSIRDKIQKEYALLYKLDIVPIDNN......STS......YRLISCNTSV**

**H19507.18.G11_CrNA**  **.TNSK.....VMI.KE...GEIKNCSFNITTSRGDKVRKDYALFSELDVVPVDND......NTS......YMLISCNTSA**

**H19507.18.C11_CrNA**  **.TNTN.....VMI.KE...GEIKNCSFNITTSRGDKVRKDYALFSELDVVPVDND......NTS......YMLISCNTSA**

**H19507.18.A11_CrNA**  **.TNSS....RVMI.KE...GEIKNCSFNITTSRGDKVRKDYALFSELDVVPVDND......NTS......YMLISCNTSA**

**H19507.18.F4_CrNA**  **.TNSS....RVMI.KE...GEIKNCSFNITTSRGDKVRKDYALFSELDVVPVDND......NTS......YMLISCNTSA**

**H19793.13.F8_CrNA**  **.TNSS...GG.TM.EG...GEIKNCSFNITTNIRDKVQKEYALFYKLDVVPIDND......NTS......YRLISCNTSV**

**H19463.8.A11_CrNA**  **.TSSS.E.G...M..R...GEIKNCSFNITTSTRDKMQKEYALFYKLDVVPIDDD......NTS......YRLISCNTSV**

**H19463.8.E10_CrNA**  **.TSSS.E.G...M..R...GEIKNCSFNITTSTRDKMQKEYALFYKLDVVPIDDD......TTS......YRLISCNTSV**

**H19474.17.1G12_CrNA**  **.TNST.E.GR.TM.EG...GEIKNCSFNITTSIRDKVQKEYALFYKLDVVPIDDD......NTS......YRLISCNTSV**

**H19474.17.2H8_CrNA**  **.TNST.E.GR.TM.EG...GEIKNCSFNITTSIRDKVQKEYALFYKLDVVPIDDD......NTS......YRLISCNTSV**

**H18814.10.1E1_CrNA**  **.TSSSGEM....M.EK...GEIKNCSFNITTGIRDKLQKEYALFYKLDVVPIDNNNTK............YRLISCNTSV**

**H18814.10.1B4_CrNA**  **.TSSSGEM....M.EK...GEIKNCSFNITTGIRDKLQKEYALFYKLDVVPIDNA......NNS......YRLISCNTSV**

**H18814.10.1C5_CrNA**  **.TSSSGEM....M.EK...GEIKNCSFNITTGIRDKLQKEYALFYKLDVVPIDNNNTK............YRLISCNTSI**

**H18814.10.1B1_CrNA**  **.TSSSGEM....M.EK...GEIKNCSFNITTGIRDKLQKEYALFYKLDVVPIDNA......NNS......YRLISCNTSV**

**H18814.10.1G2_CrNA**  **.TSSSGE....TM.EK...GEIKNCSFNITTGIRDKLQKEYALFYKLDVVPIDNA......NNS......YRLISCNTSV**

**H11668.12.F11(A)_CrNA**  **TNSSSWE..K..M.ER...GEIKNCSFNITTSIRDKMQKEYAIFYKLDIVPIDN........TS......YRLISCNTSV**

**H11668.12.C3_CrNA**  **TNSSSWE..K..M.ER...GEIKNCSFNITTSIRDKMQKEYAIFYKLDIVPIDNTSYR............YRLISCNTSV**

**H11668.12.D11_CrNA**  **TNSSSWE..K..M.ER...GEIKNCSFNITTSIRDKMQKEYAIFYKLDIVSIDN........TS......YRLISCNTSV**

**H11668.12.H9_CrNA**  **TNSSSWE..K..M.ER...GEIKNCSFNITTSIRDKMQKEYAIFYKLDIVPIDN.......TTS......YRLISCNTSV**

**H11668.12.E10_CrNA**  **TNSSSWE..K..M.ER...GEIKNCSFNITTSIRDKMQKEYAIFYKLDIVPIDN.....TSNTS......YRLISCNTSV**

**H19308.26.B1_CrNA**  **.SNSSWD..K..M.EE...GEIKNCSFNVTTSIGNKMQKEYALFYKLDVVPIDN........TS......YTLINCNTSV**

**H19308.26.F8I_CrNA**  **.SNSSWD..K..M.EE...GEIKNCSFNVTTSIGNKMQKEYALFYKLDVVPIDN........TS......YTLINCNTSV**

**H19308.26.D8_CrNA**  **.SNSSWD..K..M.EE...GEIKNCSFNVTTSIGNKMQKEYALFYKLDVVPIDN........TS......YTLINCNTSV**

**H19308.26.D1_CrNA**  **.SNSSWD..K..M.EE...GEIKNCSFNVTTSIGNKMQKEYALFYKLDVVPIDN........TS......YTLINCNTSV**

**H19308.26.E4_CrNA**  **.SNSSWD..K..M.EE...GEIKNCSFNVTTSIGNKMQKEYALFYKLDVVPIDN........TS......YTLINCNTSV**

**H19885.31.D9_CrNA**  **SNSTNWG....QM.EA...GEIKNCSFNVTRSIKNKMQKEYALFYKMDVMPIDND......NTS......YTLINCNTSV**

**H19885.31.G12_CrNA**  **SNSTNWG....QM.EA...GEIKNCSFNVTRSIKNKMQKEYALFYKMDVMPIDND......NTS......YTLINCNTSV**

**H19885.31.E2_CrNA**  **SNSTNWG....QM.EA...GEIKNCSFNVTRSIKNKMQKEYALFYKMDVMPIDND......NTS......YTLINCNTSV**

**H19885.31.G10_CrNA**  **SNSTNWG....QM.EA...GEIKNCSFNVTRSIKNKMQKEYALFYKMDVMPIDND......NTS......YTLINCNTSV**

**H19885.31.H11_CrNA**  **SNSTNWG....QM.EA...GEIKNCSFNVTRSIKNKMQKEYALFYKMDVMPIDND......NTS......YTLINCNTSV**

**H19885.31.A5_CrNA**  **SNSTNWG....QM.EA...GEIKNCSFNVTRSIKNKMQKEYALFYKMDVMPIDNDNTR............YTLINCNTSV**

**H19885.31.F8_CrNA**  **SNSTNWG....QM.EA...GEIKNCSFNVTRSIKNKMQKEYALFYKMDVMPIDND......NTS......YTLINCNTSV**

**H19885.31.H10_CrNA**  **SNSTNWG....QM.EA...GEIKNCSFNVTRSIKNKMQKEYALFYKMDVMPIDND......NTS......YTLINCNTSV**

**H19885.31.G1_CrNA**  **SNSTNWG....QM.EA...GEIKNCSFNVTRSIKNKMQKEYALFYKMDVMPIDND......NTS......YTLINCNTSV**

**H19885.31.C6_CrNA**  **SNSTNWG....QM.EA...GEIKNCSFNVTRSIKNKMQKEYALFYKMDVMPIDND......NTS......YTLINCNTSV**

**H19885.31.E11_CrNA**  **SNSTNWG....QM.EA...GEIKNCSFNVTRSIKNKMQKEYALFYKMDVMPIDND......NTS......YTLINCNTSV**

**H18969.12.9D9_CrNA**  **.TNSS..........R...GEIKNCSFKVTTSLKNK.KKEYALFYRLDIVPIDDD......NNS......YRLISCNTSV**

**H18969.12.8E6_CrNA**  **.TNSR..........R...GEIKNCSFKVTTSLRNK.KKEYALFYGLDIVPIDDD......NNS......YRLISCNTSV**

**H18969.12.8G8_CrNA**  **.TNSS..........R...GEIKNCSFKVTTSLRDK.KKEYALFYRLDIVPIDED......NNS......YRLISCNTSV**

**H18969.12.6D7_CrNA**  **.TNSS..........R...GEIKNCSFKVTTSLRDK.KKEYALFYRLDIVPIDDD......NNS......YRLISCNTSV**

**H18969.12.8B4_CrNA**  **.TNSS..........K...GEIKNCSFKVTTSLRDK.KKEYALFYRLDIVPIDDD......NNS......YRLISCNTSV**

**H18969.12.7D5_CrNA**  **.TNSR..........R...GEIKNCSFKVTTSLRDK.KKEYALFYRLDIVPIDDD......NNS......YRLISCNTSV**

**H18969.12.6C4_CrNA**  **.TNSN..........R...GEIKNCSFKVTTSLRDK.KKEYALFYRLDIVPIDDD......NNS......YRLISCNTSV**

**H18969.12.10H3_CrNA**  **.TNGN..........R...GEIKNCSFKVTTSLRDK.KKEYALFYRLDIVPIDDD......NNS......YRLISCNTSV**

**H19329.32.C9_Non-CrNA**  **SN.NSN.L......EQ.MTREIKNCSFNVTTTIRNKRQREFALLSKLDIVPIDND.........SYS...YMLINCNTSV**

**H19329.32.E6_Non-CrNA**  **SNNNSN.L......EQ.MTREIKNCSFNVTTTIRNKRQREFALLSKLDIVPIDND.........SYS...YMLINCNTSV**

**H19329.32.H7_Non-CrNA**  **SNNNSN.L......EQ.MTREIKNCSFNVTTTIRNKRQREFALLSKLDIVPIDND.........SYS...YMLINCNTSV**

**H19329.32.F1_Non-CrNA**  **SNNNSN.L......EQ.MTREIKNCSFNVTTTIRNKRQREFALLSKLDIVPIDND.........SYS...YMLINCNTSV**

**H19329.32.H5_Non-CrNA**  **SNNNSN.L......EQ.MTREIKNCSFNVTTTIRNKRQREFALLSKLDIVPIDND.........SYS...YMLINCNTSV**

**H19329.32.H9_Non-CrNA**  **SNNNSN.L......EQ.MTREIKNCSFNVTTTIRNKRQREFALLSKLDIVPIDND.........SYS...YMLINCNTSV**

**H19329.13.F12_Non-CrNA**  **SNNNSN.L......EQ.MTREIKNCSFNVTTTIRNKRQREFALLSKLDIVPIDND.........SYS...YMLINCNTSV**

**H18887.21.G2_Non-CrNA**  **T.NSSSE.G..IM......GEMKNCSFNITTNIRDKVQKEYALFYKLDLVSIDNQ.......TS......YSLISCNTSV**

**H18887.21.roD7_Non-CrNA** **T.NSSSE.G..IM......GEMKNCSFNITTNIRDKVQKEYALFYKLDLVSIDNQ.......TS......YSLISCNTSV**

**H19861.19.C10_Non-CrNA**  **T.NSS..IG.....ETMGGGEIKNCSFNITTSIRDKVQKEYALFYKLDVVPIDND..R..TNTS......YRLLSCNTSV**

**H19861.19.F2_Non-CrNA**  **T.NSS..IG.....ETMGGGEIKNCSFNITTSIRDKVQKKYALFYKLDVVPIDND..R..TNTS......YRLLSCNTSV**

**H19861.19.A6_Non-CrNA**  **T.NSS..IG.....ETMGGGEIKNCSFNITTSIRDKVQKEYALFYKLDVVPIDND..R..TNTS......YRLLSCNTSV**

**H19489.8.G5_Non-CrNA**  **T...TSGIGG.TV.EG...GEIKNCSFNITTSIRDKVQKEYALFYKLDIVPIDND....NTNNS......YRLINCNTSV**

**H19489.8.1E10_Non-CrNA**  **T.NATSGIGG.TV.EG...REIKNCSFNITTSIRDKVQKEYALFYKLDIVPIDND....NTNNN......YRLINCNTSV**

**H19489.8.1A11_Non-CrNA**  **TTNATSGIGG.TV.EG...GEIKNCSFNITTSIRDKVQKEYALFYKLDIVPIDND....NTNNS......YRLINCNTSV**

**H19489.8.1H10_Non-CrNA**  **T...TSGIGG.TV.EG...GEIKNCSFNITTSIRDKVQKEYALFYKLDIVPIDND....NTNNS......YRLINCNTSV**

**H19489.8.2A3_Non-CrNA**  **T...TSGIGG.TV.EG...GEIKNCSFNITTSIRDKVQKEYALFYKLDIVPIDND....NTNNS......YRLINCNTSV**

**H19974.11.E12_Non-CrNA**  **APNST..L.G.TMEEK...GEIKNCSFNITTSVRDKMQKEYATFYKLDIVPIDND....N..NS......YRLINCNTSV**

**H19974.11.E11_Non-CrNA**  **APNST..L.R.TMEEK...GEIKNCSFNITTSVRDKMQKEYATFYKLDIVPIDND....N..NS......YRLINCNTSV**

**H19792.9.F6_Non-CrNA**  **TNNSSW..GK..M.EE...GEIKNCSFNITTSI.GKLQKEYALFYKLDVVPIDND........S......YTLINCNTSV**

**H19792.9.B1_Non-CrNA**  **TNNSSW..GK..M.EE...GEIKNCSFNITTCI.GKLQKEYALFYKLDVVPIDND........S......YTLINCNTSV**

**H19792.9.F1_Non-CrNA**  **TNNSSW..GK..M.EE...GEIKNCSFNITTSI.GKLQKEYALFYKLDVVPVDND........S......YTLINCNTSV**

**H19792.9.D6_Non-CrNA**  **TNNSSW..GK..M.EE...GEIKNCSFNITTSI.GKLQKEYALFYKLDVVPIDND........S......YTLINCNTSV**

**H19792.9.C10_Non-CrNA**  **TNNSIW..GK..M.EE...GEIKNCSFNITTSI.GKLQKEYALFYKLDVVPIDND........S......YTLINCNTSV**

**H18880.10.20_Non-CrNA**  **..ITSS..G..........GEIKNCSFNITTSIRDKVKQEYALFYKLDVGPIDDG......NTT..TN..YRLINCNTSV**

**H18880.10.21_Non-CrNA**  **..ITSS..G..........GEIKNCSFNITTSIRDKVKQEYALFYKLDVVPIDDG......NTT..TN..YRLINCNTSV**

**H19961.14.F10_Non-CrNA**  **TNNTSS.GGE.TV.EK...GEIKNCSFNITTNIRDKVQKVYATFYKLDIVPIDD...KTNNNNTIYTN..YRLISCNTSV**

**H19961.14.E8_Non-CrNA**  **N..TSS.GGG.TV.EK...GEIKNCSFNITTNIRDKVQKVYATFYKLDIVPIDD...KTNNNNTNYTN..YRLISCNTSV**

**H19961.14.G4_Non-CrNA**  **TNNTSS.GGE.TV.EK...GEIKNCSFNITTNIRDKVQKVYATFYKLDIVPIDD...KTNNNNTNYTN..YRLISCNTSV**

**H19961.14.B10_Non-CrNA**  **TNNTSS.GGE.TV.EK...GEIKNCSFNITTNIRDKVQKVYATFYKLDIVPIDD...KTNNNNTNYTN..YRLISCNTSV**

**H19961.14.F9_Non-CrNA**  **TNNTSS.GGE.TV.EK...GEIKNCSFNITTNIRDKVQKVYATFYKLDIVPIDD...KTNNNNTNYTN..YRLISCNTSV**

**H19576.9.H1_Non-CrNA**  **.INNSS.VG..TLKE....GEIKNCSFNITTNIRDKMQKEYALFYKLDIVPIDNDNTR............YRLISCNTSV**

**H19576.9.F4_Non-CrNA**  **..TTSS.IGG.TWK.G...GEIKNCSFNITTNIRDKMQKEYALFYELDVVPIDND......NTS......YRLISCNTSV**

250 260 270 280 290 300 310 320

....|....|....|....|....|....|....|....|....|....|....|....|....|....|....|....|

**HXB2/1-856**  **ITQACPKVSFEPIPIHYCAPAGFAILKCNNKTFNGTGPCTNVSTVQCTHGIRPVVSTQLLLNGSLAEEEVVIRSVNFTDN**

**H18818.6.1D2_CrNA**  **ITQACPKITFEPIPIQFCTPAGFAILKCNNKKFNGKGPCTNVSTVQCTHGIRPVVSTQLLLNGSLAEEEVIIRSDNFTDN**

**H18818.6.1C3_CrNA**  **ITQACPKITFEPIPIHFCTPAGFAILKCNNKKFNGKGPCTNVSTVQCTHGIRPVVSTQLLLNGSLAEEEVIIRSNNFTDN**

**H18818.6.1A6_CrNA**  **ITQACPKITFEPIPIHFCTPAGFAILKCNNKKFNGKGPCTNVSTVQCTHGIRPVVSTQLLLNGSLAEEEVIIRSDNFTDN**

**H18818.6.1G12_CrNA**  **ITQACPKITFEPIPIHFCTPAGFAILKCNNKKFNGKGPCTNVSTVQCTHGIRPVVSTQLLLNGSLAEEEVIIRSDNFTDN**

**H19829.11.H5_CrNA**  **ITQACPKISFEPIPIHFCTPAGFAILKCNDKKFNGKGPCTNVSTVQCTHGIRPVVSTQLLLNGSLAEEEVIIRSDNFTDN**

**H19829.11.A2_CrNA**  **ITQACPKISFEPIPIHFCTPAGFAILKCNDKKFNGKGPCTNVSTVQCTHGIRPVVSTQLLLNGSLAEEEVVIRSDNFTDN**

**H19829.11.E8_CrNA**  **ITQACPKISFEPIPIHFCTPAGFAILKCNDKKFNGKGPCTNVSTVQCTHGIRPVVSTQLLLNGSLAEEEVVIRSDNFTDN**

**H19829.11.B4_CrNA**  **ITQACPKISFEPIPIHFCTPAGFAILKCNDKKFNGKGPCTNVSTVQCTHGIRPVVSTQLLLNGSLAEEEVIIRSDNFTDN**

**H19829.11.A4_CrNA**  **ITQACPKISFEPIPIHFCTPAGFAILKCNDKKFNGKGPCTNVSTVQCTHGIRPVVSTQLLLNGSLAEEEVIIRSDNFTDN**

**H19829.11.G8_CrNA**  **ITQACPKISFEPIPIHFCTPAGFAILKCNDKKFNGKGPCTNVSTVQCTHGIRPVVSTQLLLNGSLAEEEVIIRSDNFTDN**

**H19999.7.1G10_CrNA**  **ITQACPKVSFEPIPIHYCAPAGFAILKCNNKTFNGKGPCTNVSTVQCTHGIRPVVSTQLLLNGSLAEEEIVIRSDNITDN**

**H19999.7.2G7_CrNA**  **ITQACPKVSFEPIPIHYCAPAGFAILKCNNKTFNGKGPCTNVSTVQCTHGIRPVVSTQLLLNGSLAEEEIVIRSDNITDN**

**H19999.7.2D5_CrNA**  **ITQACPKVSFEPIPIHYCAPAGFAILKCNNKTFNGKGPCTNVSTVQCTHGIRPVVSTQLLLNGSLAEEEIVIRSDNITDN**

**H19999.7.1B2_CrNA**  **ITQACPKVSFEPIPIHYCAPAGFAILKCNNKTFNGKGPCTNVSTVQCTHGIRPVVSTQLLLNGSLAEEKIVIRSDNITDN**

**H19999.7.1D2_CrNA**  **ITQACPKVSFEPIPIHYCAPAGFAILKCNNKTFNGKGPCTNVSTVQCTHGIRPVVSTQLLLNGSLAEKEIVIRSDNITDN**

**H19507.18.G11_CrNA**  **ITRACPKISFEPIPIHFCTPAGFALLKCNDKKFNGSGPCTNVSTVQCTHGIRPVVSTQLLLNGSIAEEEIVIRSENFTNN**

**H19507.18.C11_CrNA**  **ITRACPKISFEPIPIHFCTPAGFALLKCNDKKFNGSGPCTNVSTVQCTHGIRPVVSTQLLLNGSIAEEEIVIRSENFTNN**

**H19507.18.A11_CrNA**  **ITRACPKISFEPIPIHFCTPAGFALLKCNDKKFNGSGPCTNVSTVQCTHGIRPVVSTQLLLNGSIAEEEIVIRSENFTNN**

**H19507.18.F4_CrNA**  **ITRACPKISFEPIPIHFCTPAGFALLKCNDKKFNGSGPCTNVSTVQCTHGIRPVVSTQLLLNGSIAEEEIVIRSENFTNN**

**H19793.13.F8_CrNA**  **ITQACPKVSFEPIPIHYCAPAGFAILKCKDKKFNGTGPCKNVSTVQCTHGIRPVVSTQLLLNGSLAEEEVVIRSANFTDN**

**H19463.8.A11_CrNA**  **ITQACPKVSFEPIPIHYCTPAGFALLKCNDKKFNGTGPCTNVSTVQCTHGIRPVVSTQLLLNGSLAEGEVVIRSVNFTNN**

**H19463.8.E10_CrNA**  **ITQACPKVSFEPIPIHYCTPAGFALLKCNDKKFNGTGPCTNVSTVQCTHGIRPVVSTQLLLNGSLAEGEVVIRSVNFTNN**

**H19474.17.1G12_CrNA**  **ITQACPKISFEPIPIHYCAPAGFAILKCNNKTFNGKGPCTNVSTVQCTHGIRPVVSTQLLLNGSLAEEEVVIRSENFTDN**

**H19474.17.2H8_CrNA**  **ITQACPKISFEPIPIHYCAPAGFAILKCNNKTFNGKGPCTNVSTVQCTHGIRPVVSTQLLLNGSLAEKEVVIRSENFTDN**

**H18814.10.1E1_CrNA**  **ITQACPKVSFEPIPIHYCAPAGFAILKCNDKEFNGTGPCANVSTVQCTHGIRPVVSTQLLLNGSLAEEEVVIRSANFTDN**

**H18814.10.1B4_CrNA**  **ITQACPKVSFEPIPIHYCAPAGFAILKCNDKEFNGTGPCANVSTVQCTHGIRPVVSTQLLLNGSLAEEEVVIRSANFTDN**

**H18814.10.1C5_CrNA**  **ITQACPKVSFEPIPIHYCAPAGFAILKCNDKEFNGTGPCANVSTVQCTHGIRPVVSTQLLLNGSLAEEEVVIRSANFTDN**

**H18814.10.1B1_CrNA**  **ITQACPKVSFEPIPIHYCAPAGFAILKCNDKTFNGTGPCANVSTVQCTHGIRPVVSTQLLLNGSLAEEEVVIRSANFTDN**

**H18814.10.1G2_CrNA**  **ITQACPKVSFEPIPIHYCAPAGFAILKCNDKEFNGTGPCANVSTVQCTHGIRPVVSTQLLLNGSLAEEEVVIRSANFTDN**

**H11668.12.F11(A)_CrNA**  **ITQACPKVSFEPIPIHYCAPAGFAILKCNDKKFNGTGPCTNVSTVQCTHGIKPVVSTQLLLNGSLAEEEVVIRSENFTDN**

**H11668.12.C3_CrNA**  **ITQACPKISFEPIPIHYCAPAGFAILKCNDKKFNGTGPCTNVSTVQCTHGIKPVVSTQLLLNGSLAEEEVVIRSENFTDN**

**H11668.12.D11_CrNA**  **ITQACPKISFEPIPIHYCAPAGFAILKCNDKKFNGTGPCTNVSTVQCTHGIKPVVSTQLLLNGSLAEEEIVIRSENFTDN**

**H11668.12.H9_CrNA**  **ITQACPKVSFEPIPIHYCAPAGFAILKCNDKKFNGTGPCTNVSTVQCTHGIKPVVSTQLLLNGSLAEEEVVIRSENFTDN**

**H11668.12.E10_CrNA**  **ITQACPKISFEPIPIHYCAPAGFAILKCNDKKFNGTGPCTNVSTVQCTHGIKPVVSTQLLLNGSLAEEEVVIRSENFTDN**

**H19308.26.B1_CrNA**  **ITQACPKISFEPIPIHYCAPAGFAILKCNDKKFNGTGPCTNVSTVQCTHGIRPVVSTQLLLNGSLSEGEIIIRSENFTDN**

**H19308.26.F8I_CrNA**  **ITQACPKISFEPIPIHYCAPAGFAILKCNDKKFNGTGPCTNVSTVQCTHGIRPVVSTQLLLNGSLSEGEIIIRSENFTDN**

**H19308.26.D8_CrNA**  **ITQACPKISFEPIPIHYCAPAGFAILKCNDKKFNGTGPCTNVSTVQCTHGIRPVVSTQLLLNGSLSEGEIIIRSKNFTDN**

**H19308.26.D1_CrNA**  **ITQACPKISFEPIPIHYCAPAGFAILKCNDKKFNGTGPCTNVSTVQCTHGIRPVVSTQLLLNGSLSEGEIIIRSENFTDN**

**H19308.26.E4_CrNA**  **ITQACPKISFEPIPIHYCAPAGFAILKCNDKKFNGTGPCTNVSTVQCTHGIRPVVSTQLLLNGSLSEGEIIIRSENFTDN**

**H19885.31.D9_CrNA**  **ITQACPKVSFEPIPIHYCAPAGFAILKCNDKKFNGTGPCKNVSTVQCTHGIKPVVSTQLLLNGSLAEQEVVIRSENFTNN**

**H19885.31.G12_CrNA**  **ITQACPKVSFEPIPIHYCAPAGFAILKCNDKKFNGTGPCKNVSTVQCTHGIKPVVSTQLLLNGSLAEQEVVIRSENFTNN**

**H19885.31.E2_CrNA**  **ITQACPKVSFEPIPIHYCAPGGFAILKCNDKKFNGTGPCKNVSTVQCTHGIKPVVSTQLLLNGSLAEQEVVIRSENFTNN**

**H19885.31.G10_CrNA**  **ITQACPKVSFEPIPIHYCAPAGFAILKCNDKKFNGTGPCKNVSTVQCTHGIKPVVSTQLLLNGSLAEQEVVIRSENFTNN**

**H19885.31.H11_CrNA**  **ITQACPKVSFEPIPIHYCAPAGFAILKCNDKKFNGTGPCKNVSTVQCTHGIKPVVSTQLLLNGSLAEQEVVIRSENFTNN**

**H19885.31.A5_CrNA**  **ITQACPKVSFEPIPIHYCAPAGFAILKCNDKKFNGTGPCKNVSTVQCTHGIKPVVSTQLLLNGSLAEQEVVIRSENFTNN**

**H19885.31.F8_CrNA**  **ITQACPKVSFEPIPIHYCAPAGFAILKCNDKKFNGTGPCKNVSTVQCTHGIKPVVSTQLLLNGSLAEQEVVIRSENFTNN**

**H19885.31.H10_CrNA**  **ITQACPKVSFEPIPIHYCAPGGFAILKCNDKKFNGTGPCKNVSTVQCTHGIKPVVSTQLLLNGSLAEQEVVIRSENFTNN**

**H19885.31.G1_CrNA**  **ITQACPKVSFEPIPIHYCAPAGFAILKCNDKKFNGTGPCKNVSTVQCTHGIKPVVSTQLLLNGSLAEQEVVIRSENFTNN**

**H19885.31.C6_CrNA**  **ITQACPKVSFEPIPIHYCAPAGFAILKCNDKKFNGTGPCKNVSTVQCTHGIKPVVSTQLLLNGSLAEQEVVIRSENFTNN**

**H19885.31.E11_CrNA**  **ITQACPKVSFEPIPIHYCAPAGFAILKCNDKKFNGTGPCKNVSTVQCTHGIKPVVSTQLLLNGSLAEQEVVIRSENFTNN**

**H18969.12.9D9_CrNA**  **ITQACPKVTFEPIPIHYCTPAGFALLKCNDKKFSRKGPCTNVSTVQCTHGIRPVVSTQLLLNGSLAEEEIVIRSENFTNN**

**H18969.12.8E6_CrNA**  **ITQACPKVTFEPIPIHYCTPAGFALLKCNDKKFSGRGPCTNVSTVQCTHGIRPVVSTQLLLNGSLAEEEIVIRSENFTNN**

**H18969.12.8G8_CrNA**  **ITQACPKVTFEPIPIHYCTPAGFALLKCNDKKFSGKGPCTNVSTVQCTHGIRPVVSTQLLLNGSLAEEEIVIRSENFTNN**

**H18969.12.6D7_CrNA**  **ITQACPKVTFEPIPIHYCTPAGFALLKCNDKKFSGKGPCTNVSTVQCTHGIRPVVSTQLLLNGSLAEEEIVIRSENFTNN**

**H18969.12.8B4_CrNA**  **ITQACPKVTFEPIPIHYCTPAGFALLKCNDKKFSGKGPCTNVSTVQCTHGIRPVVSTQLLLNGSLAEEEIVIRSENFTNN**

**H18969.12.7D5_CrNA**  **ITQACPKVTFEPIPIHYCTPAGFALLKCNDKKFSGKGPCTNVSTVQCTHGIRPVVSTQLLLNGSLAEEEIVIRSENFTNN**

**H18969.12.6C4_CrNA**  **ITQACPKVTFEPIPIHYCTPAGFALLKCNDKKFSGKGPCTNVSTVQCTHGIRPVVSTQLLLNGSLAEEEIVIRSENFTNN**

**H18969.12.10H3_CrNA**  **ITQACPKVTFEPIPIHYCTPAGFALLKCNDKKFSGKGPCTNVSTVQCTHGIRPVVSTQLLLNGSLAEEEIVIRSENFTNN**

**H19329.32.C9_Non-CrNA**  **ITQACPKVSFQPIPIHYCTPAGFAILKCNDKKFNGTGPCKNVSTVQCTHGIRPVVSTQLLLNGSLAEEEVVIRSKNFTDN**

**H19329.32.E6_Non-CrNA**  **ITQACPKVSFQPIPIHYCTPAGFAILKCNDKKFNGTGPCKNVSTVQCTHGIRPVVSTQLLLNGSLAEEEVVIRSKNFTDN**

**H19329.32.H7_Non-CrNA**  **ITQACPKVSFQPIPIHYCTPAGFAILKCNDKKFNGTGPCKNVSTVQCTHGIRPVVSTQLLLNGSLAEEEVVIRSKNFTDN**

**H19329.32.F1_Non-CrNA**  **ITQACPKVSFQPIPIHYCTPAGFAILKCNDKKFNGTGPCKNVSTVQCTHGIRPVVSTQLLLNGSLAEEEVVIRSKNFTDN**

**H19329.32.H5_Non-CrNA**  **ITQACPKVSFQPIPIHYCTPAGFAILKCNDKKFNGTGPCKNVSTVQCTHGIRPVVSTQLLLNGSLAEEEVVIRSKNFTDN**

**H19329.32.H9_Non-CrNA**  **IKQACPKVSFQPIPIHYCTPAGFAILKCNDKKFNGTGPCKNVSTVQCTHGIRPVVSTQLLLNGSLAEEEVVIRSKNFTDN**

**H19329.13.F12_Non-CrNA**  **ITQACPKVSFQPIPIHYCTPAGFAILKCNDKKFNGTGPCKNVSTVQCTHGIRPVVSTQLLLNGSLAEEEVVIRSKNFTDN**

**H18887.21.G2_Non-CrNA**  **TTQACPKVSFEPIPIHYCAPAGFAILKCNNKTFNGTGPCTNVSTVQCTHGIRPVVSTQLLLNGSLAEEEVVIRSENFTNN**

**H18887.21.roD7_Non-CrNA** **TTQACPKVSFEPIPIHYCAPAGFAILKCNNKTFNGTGPCTNVSTVQCTHGIRPVVSTQLLLNGSLAEEEVVIRSENFTNN**

**H19861.19.C10_Non-CrNA**  **ITQACPKVTFEPIPIHYCAPAGFAILKCNNKKFNGTGPCTNVSTVQCTHGIRPVVSTQLLLNGSLAEDEVAIRSSNFTDN**

**H19861.19.F2_Non-CrNA**  **ITQACPKVTFEPIPIHYCAPAGFAILKCNNKKFNGTGPCTNVSTVQCTHGIRPVVSTQLLLNGSLAEDEVAIRSSNFTDN**

**H19861.19.A6_Non-CrNA**  **ITQACPKVTFEPIPIHYCAPAGFAILKCNNKKFNGTGPCTNVSTVQCTHGIRPVVSTQLLLNGSLAEDEVAIRSSNFTDN**

**H19489.8.G5_Non-CrNA**  **IKQACPKVSFEPIPIHYCAPAGFAILKCNDKKFNGTGPCTNVSTVQCTHGIRPVVSTQLLLNGSLAEEEVAIRSQNFTNN**

**H19489.8.1E10_Non-CrNA**  **IKQACPKVSFEPIPIHYCAPAGFAILKCNDKKFNGTGPCTNVSTVQCTHGIRPVVSTQLLLNGSLAEEEVAIRSQNFTNN**

**H19489.8.1A11_Non-CrNA**  **IKQACPKVSFEPIPIHYCAPAGFAILKCNDKKFNGTGPCTNVSTVQCTHGIRPVVSTQLLLNGSLAEEEVAIRSQNFTNN**

**H19489.8.1H10_Non-CrNA**  **IKQACPKVSFEPIPIHYCAPAGFAILKCNDKKFNGTGPCTNVSTVQCTHGIRPVVSTQLLLNGSLAEEEVAIRSQNFTNN**

**H19489.8.2A3_Non-CrNA**  **IKQACPKVSFEPIPIHYCAPAGFAILKCNDKKFNGTGPCTNVSTVQCTHGIRPVVSTQLLLNGSLAEEEVAIRSQNFTNN**

**H19974.11.E12_Non-CrNA**  **ITQACPKVSFEPIPIHYCAPAGFAILKCNNKTFNGTGPCTNVSTVQCTHGIRPVVSTQLLLNGSLAEEEIVIRSENFTDN**

**H19974.11.E11_Non-CrNA**  **ITQACPKVSFEPIPIHYCAPAGFAILKCNNKTFNGTGPCTNVSTVQCTHGIRPVVSTQLLLNGSLAEEEIVIRSENFTDN**

**H19792.9.F6_Non-CrNA**  **ITQACPKVSFEPIPIHFCTPAGFAILQCNNKKFNGTGPCTNVSTVQCTHGIRPVVSTQLLLNGSLSEGEVVIRSENFTDN**

**H19792.9.B1_Non-CrNA**  **ITQACPKVSFEPIPIHFCTPAGFAILKCNNKKFNGTGPCTNVSTVQCTHGIRPVVSTQLLLNGSLSEGEVVIRSENFTDN**

**H19792.9.F1_Non-CrNA**  **ITQACPKVSFEPIPIHFCTPAGFAILKCNNKKFNGTGPCTNVSTVQCTHGIRPVVSTQLLLNGSLSEGEVVIRSENFTDN**

**H19792.9.D6_Non-CrNA**  **ITQACPKVSFEPIPIHFCTPAGFAILQCNNKKFNGTGPCTNVSTVQCTHGIRPVVSTQLLLNGSLSEGEVVIRSENFTDN**

**H19792.9.C10_Non-CrNA**  **ITQACPKVSFEPIPIHFCTPAGFAILKCNNKKFNGTGPCTNVSTVQCTHGIRPVVSTQLLLNGSLSEGEVVIRSENFTDN**

**H18880.10.20_Non-CrNA**  **ITQACPKVSFEPIPIHYCTPAGFAILKCNDKKFNGTGSCTNVSTVQCTHGIRPVVSTQLLLNGSLAEREVAIRSNNFTDN**

**H18880.10.21_Non-CrNA**  **ITQACPKVSFEPIPIHYCTPAGFAILKCNDKKFNGTGSCTNVSTVQCTHGIRPVVSTQLLLNGSLAEREVAIRSNNFTDN**

**H19961.14.F10_Non-CrNA**  **LTQACPKISFEPIPIHYCAPAGFAILKCNNKTFNGKGPCTNVSTVQCTHGIRPVVSTQLLLNGSLAEEEVVIRSENFADN**

**H19961.14.E8_Non-CrNA**  **LTQACPKISFEPIPIHYCAPAGFAILKCNNKTFNGKGPCTNVSTVQCTHGIRPVVSTQLLLNGSLAEEEVVIRSENFTDN**

**H19961.14.G4_Non-CrNA**  **LTQACPKISFEPIPIHYCAPAGFAILKCNNKTFNGKGPCTNVSTVQCTHGIRPVVSTQLLLNGSLAEEEVVIRSKNFTDN**

**H19961.14.B10_Non-CrNA**  **LTQACPKISFEPIPIHYCAPAGFAILKCNNKTFNGKGPCTNVSTVQCTHGIRPVVSTQLLLNGSLAEEEVVIRSENFTDN**

**H19961.14.F9_Non-CrNA**  **LTQACPKISFEPIPIHYCAPAGFAILKCNNKTFNGKGPCTNVSTVQCTHGIRPVVSTQLLLNGSLAEEEVVIRSENFTDN**

**H19576.9.H1_Non-CrNA**  **ITQACPKVSFEPIPIHYCAPAGFAILKCNNKTFDGKGPCTNVSTVQCTHGIRPVVSTQLLLNGSLAEEEVVIRSENITDN**

**H19576.9.F4_Non-CrNA**  **ITQACPKVSFEPIPIHYCAPAGFAILKCNNKTFDGKGPCTNVSTVQCTHGIRPVVSTQLLLNGSLAEEEVVIRSENITDN**

330 340 350 360 370 380 390 400

....|....|....|....|....|....|....|....|....|....|....|....|....|....|....|....|

**HXB2/1-856**  **AKTIIVQLNTSVEINCTRPNNNTRKRIRIQRGPGRAFVTIGK.IGNMRQAHCNISRAKWNNTLKQIASKLREQFGNNKTI**

**H18818.6.1D2_CrNA**  **AKTIIVQLNESVVINCTRPNNNTRKSINI..GPGRAFYTTGEIIGDIRQAHCNLSRTQWNNTLKQIAIKLREQF.ENKTI**

**H18818.6.1C3_CrNA**  **VKTIIVQLNESVVINCTRPNNNTRKSINI..GPGRAFYTTGEIIGDIRQAHCNLSRTQWNNTLKQIAIKLREQF.ENKTI**

**H18818.6.1A6_CrNA**  **AKTIIVQLNESVVINCTRPNNNTRKSINI..GPGRAFYTTGEIIGDIRQAHCNLSRTQWNNTLKQIAIKLREQF.ENKTI**

**H18818.6.1G12_CrNA**  **AKTIIVQLNESVVINCTRPNNNTRKSINI..GPGRAFYTTGEIIGDIRQAHCNLSRTQWNNTLKQIAIKLREQF.ENKTI**

**H19829.11.H5_CrNA**  **AKTIIVQLNESVVINCTRPNNNTRKSINI..GPGRAFYTTGEIIGEIRQAHCNLSRTQWNNTLKQIAIKLREQF.ENKTI**

**H19829.11.A2_CrNA**  **AKTIIVQLNESVVINCTRPNNNTRKSINI..GPGRAFYTTGDIIGDIRQAHCNLSRTQWNNTLKQIAIKLREQF.ENKTI**

**H19829.11.E8_CrNA**  **AKTIIVQLNESVVINCTRPNNNTRKSINI..GPGRAFYTTGDIIGDIRQAHCNLSRTQWNNTLKQIAIKLREQF.ENKTI**

**H19829.11.B4_CrNA**  **AKTIIVQLNESVVINCTRPNNNTRKSINI..GPGRAFYTTGDIIGDIRQAHCNLSRTQWNNTLKQIAIKLREQF.ENKTI**

**H19829.11.A4_CrNA**  **AKTIIVQLNESVVINCTRPNNNTRKSINI..GPGRAFYTTGDIIGDIRQAHCNLSRTQWNNTLKQIAIKLREQF.ENKTI**

**H19829.11.G8_CrNA**  **AKTIIVQLNESVVINCTRPNNNTRKSINI..GPGRAFYTTGEIIGDIRQAHCNLSRTQWNNTLKQIAIKLREQF.ENKTI**

**H19999.7.1G10_CrNA**  **AKTIIVQLKEAVQINCTRPNNNTRKSIHI..GPGKAFYATGEIIGDIRQAHCNLSRVDWENTLKQIAEKLREQF.RNKTI**

**H19999.7.2G7_CrNA**  **AKTIIVQLKEAVQINCTRPNNNTRKSIHI..GPGKAFYATGEIIGDIRQAHCNLSRVDWENTLKQIAEKLREQF.RNKTI**

**H19999.7.2D5_CrNA**  **AKTIIVQLKEAVQINCTRPNNNTRKSIHI..GPGKAFYATGEIIGDIRQAHCNLSRADWENTLKQIAEKLREQF.RNKTI**

**H19999.7.1B2_CrNA**  **AKTIIVQLKEAVQITCTRPNNNTRKSIHI..GPGRAFYATGEIIGDIRQAHCNLTRGNWEKTLGQIAEKLREQF.RNKTI**

**H19999.7.1D2_CrNA**  **AKTIIVQLTKAVQITCTRPNNNTRKSIHI..GPGKAFYATGEIIGDIRQAHCNLSRGEWEDTLKKVAEKLREQF.RNKTI**

**H19507.18.G11_CrNA**  **AKIIIVQLNKSIAINCTRPNNNTRKGIHM..GPGRTIYATGEVIGDIRQAHCNLSREEWNNTLKQVVTKLKEQFG.NKTI**

**H19507.18.C11_CrNA**  **AKIIIVQLNKSIAINCTRPNNNTRKSIHM..GPGRAFFATGEVIGDIRQAHCNLSREEWNNTLKQVVTKLKEQFG.NKTI**

**H19507.18.A11_CrNA**  **AKIIIVQLNKSIAINCTRPNNNTRKSIHM..GPGRAFFATGEVIGDIRQAHCNLSREEWNNTLKQVVTKLKEQFG.NKTI**

**H19507.18.F4_CrNA**  **AKIIIVQLNKSIAINCTRPNNNTRKSIHM..GPGRAFFATGEVIGDIRQAHCNLSREEWNNTLKQVVTKLKEQFG.NKTI**

**H19793.13.F8_CrNA**  **AKTIIVQLKESVEINCTRPNNNTRKGIHI..GPGRAFYTTGEIIGDIRQAHCNLSRAKWNDTLSQIVKKLREQFG.NKTI**

**H19463.8.A11_CrNA**  **AKTIIVQLNESVVINCTRPNNNTRKSINI..GPGRAFYTTGEIIGDIRQAHCNLSIATWNNTLKQIVTKLREQFGNNKTI**

**H19463.8.E10_CrNA**  **AKTIIVQLNESVVINCTRPNNNTRKSINI..GPGRALYTTGEIIGDIRQAHCNLSIATWNNTLKQIVTKLREQFGNNKTI**

**H19474.17.1G12_CrNA**  **AKTIIVQLKEPVEINCTRPNNNTRKSIHI..GPGRAFYTTGEIIGNIRQAHCNLSRAQWNNTLKQIVIKLREQF.RNKTI**

**H19474.17.2H8_CrNA**  **AKTIIVQLKEPVEINCTRPNNNTRKSIHI..GPGRAFYTTGEIIGNIRQAQCNLSRAQWNNTLKQIVIKLREQF.RNKTI**

**H18814.10.1E1_CrNA**  **AKTIIVQLNESVEINCTRPNNNTRKSIHM..GPGRAFYTTGDIIGDIRQAHCNISGTKWNNTLQQIVKKLREQF.NNKTI**

**H18814.10.1B4_CrNA**  **AKTIIVQLNESVEINCTRPNNNTRKSIHI..GPGRAFYTTGDIIGDIRQAHCNISGTKWNNTLQQIVKKLREQF.NNKTI**

**H18814.10.1C5_CrNA**  **AKTIIIQLNESVEINCTRPNNNTRKSIHI..GPGRAFYTTGDIIGDIRQAYCNISRTKWNNTLQQIVKKLREQF.NNKTI**

**H18814.10.1B1_CrNA**  **AKTIIVQLNESVEINCTRPNNNTRKSIHI..GPGRAFYTTGDIIGDIRQAYCNISRTKWNNTLQQIVKKLREQF.NNKTI**

**H18814.10.1G2_CrNA**  **AKTIIVQLNESVEINCTRPNNNTRKSIHI..GPGRAFYTTGDIIGDIRQAYCNISRTKWNNTLQQIVKKLREQF.NNKTI**

**H11668.12.F11(A)_CrNA**  **TKTIIVQLKESVEINCTRPNNNTRKGINI..GPGRAFYTTRDIIGDIRQAHCNISRAKWNDTLKQIVDKLREQF.RNKTI**

**H11668.12.C3_CrNA**  **TKTIIVQLKESVEINCTRPNNNTRKGINI..GPGRAFYTTRDIIGDIRQAHCNISRAKWNNTLKQIVNKLREQF.RNKTI**

**H11668.12.D11_CrNA**  **TKTIIVQLKESVEINCTRPNNNTRKGINI..GPGRAFYTTRDIIGDIRQAHCNISRAKWNNTLKQIVDKLREQF.RNKTI**

**H11668.12.H9_CrNA**  **AKTIIVQLKESVEINCTRPNNNTRKGINL..GPGRAFYTTRDIIGDIRQAHCNISRAKWNNTLKQIVDKLREQF.KNKTI**

**H11668.12.E10_CrNA**  **AKTIIVQLKESVEINCTRPNNNTRKGIHL..GPGRAFYTTRDIIGDIRQAHCNISRAKWNNTLKQIVDKLREQF.RNKTI**

**H19308.26.B1_CrNA**  **AKTIIVQLNKSIAINCTRPNNNTRKSIPI..GPGRAFYATGDIIGDIRKAHCNLSRTEWNNTLKQVATKLKEQF.KKETI**

**H19308.26.F8I_CrNA**  **AKTIIVQLNKSIAINCTRPNNNTRKSIPI..GPGRAFYATGDIIGDIRKAHCNLSRTEWNNTLKQVATKLKEQF.KKETI**

**H19308.26.D8_CrNA**  **AKTIIVQLNKSIAINCTRPNNNTRKSIPI..GPGRAFYATGDIIGDIRKAHCNLSRTEWNNTLKQVATKLKEQF.KKETI**

**H19308.26.D1_CrNA**  **AKTIIVQLNKSIAINCTRPNNNTRKSIPI..GPGRAFYATGDIIGDIRKAHCNLSRTEWNNTLKQVATKLKEQF.KKETI**

**H19308.26.E4_CrNA**  **AKTIIVQLNKSIAINCTRPNNNTRKSIPI..GPGRAFYATGDIIGDIRKAHCNLSRTEWNNTLKQVATKLKEQF.KKETI**

**H19885.31.D9_CrNA**  **AKTIIVQLNESVVINCTRPNNNTRKSINI..GPGRAFYATGEIIGDIRQAHCNLSRLDWNRTLGQIVSKLKEQFG.NKTI**

**H19885.31.G12_CrNA**  **AKTIIVQLNESVVINCTRPNNNTRKSINI..GPGRAFYATGEIIGDIRQAHCNLSRLDWNRTLGQIVSKLKEQFG.NKTI**

**H19885.31.E2_CrNA**  **AKTIIVQLNESVVINCTRPNNNTRKSIHI..GPGRAFYATGEIIGDIRQAHCNLSRLDWNRTLGQIVSKLKEQFG.NKTI**

**H19885.31.G10_CrNA**  **AKTIIVQLNESVVINCTRPNNNTRKSINI..GPGRAFYATGEIIGDIRQAHCNLSRLDWNRTLGQIVSKLKEQFG.NKTI**

**H19885.31.H11_CrNA**  **AKTIIVQLNESVVINCTRPNNNTRKSINI..GPGRAIYATGEIIGDIRQAHCNLSRLDWNRTLGQIVSKLKEQFG.NKTI**

**H19885.31.A5_CrNA**  **AKTIIVQLNESVVINCTRPNNNTRKSINI..GPGRAFYATGEIIGDIRQAHCNLSRLDWNRTLGQIVSKLKEQFG.NKTI**

**H19885.31.F8_CrNA**  **AKTIIVQLNESVVINCTRPNNNTRKSINI..GPGRAFYATGEIIGDIRQAHCNLSRLDWNRTLGQIVSKLKEQFG.NKTI**

**H19885.31.H10_CrNA**  **AKTIIVQLNESVVINCTRPNNNTRKSINI..GPGRAFYATGEIIGDIRQAHCNLSRLDWNRTLGQIVSKLKEQFG.NKTI**

**H19885.31.G1_CrNA**  **AKTIIVQLNESVVINCTRPNNNTRKSINI..GPGRAFYATGEIIGDIRQAHCNLSRLDWNRTLGQIVSKLKEQFG.NKTI**

**H19885.31.C6_CrNA**  **AKTIIVQLNESVVINCTRPNNNTRKSINI..GPGRAFYATGEIIGDIRQAHCNLSRLDWNRTLGQIVSKLKEQFG.NKTI**

**H19885.31.E11_CrNA**  **AKTIIVQLNESVVINCTRPNNNTRKSINI..GPGRAFYATGEIIGDIRQAHCNLSRLDWNRTLGQIVSKLKEQFG.NKTI**

**H18969.12.9D9_CrNA**  **AKTIIVQLNKSVEIYCTRPNNNTRKSIHV..GPGKTLYATGDIIGDIRQAHCNLSRAKWNDTLKQIVIKLRKQF.RNRTI**

**H18969.12.8E6_CrNA**  **AKTIIVQLNESVEIYCTRPNNNTRKSIHV..GPGKTLYATGDIIGNIRQAHCNLSRAKWNDTLKQIVIKLRKQF.RNRTI**

**H18969.12.8G8_CrNA**  **AKTIIVQLNESVEIYCTRPNNNTRKSIHV..GPGKTLYATGDIIGDIRQAHCNLSRAKWNDTLKQIVIKLRKQF.RNRTI**

**H18969.12.6D7_CrNA**  **AKTIIVQLNESVEIYCTRPNNNTRKSIHV..GPGKTLYATGDIIGNIRQAHCNLSRAKWNDTLKQIVIKLRKQF.RNRTI**

**H18969.12.8B4_CrNA**  **AKTIIVQLNESVEIYCTRPNNNTRKSIHV..GPGKTLYATGDIIGDIRQAHCNLSRAKWNDTLKQIVIKLRKQF.RNRTI**

**H18969.12.7D5_CrNA**  **AKTIIVQLNESVEIYCTRPNNNTRKSIHV..GPGKTLYATGDIIGNIRQAHCNLSRAKWNDTLKQIVIKLRKQF.RNRTI**

**H18969.12.6C4_CrNA**  **AKTIIVQLNESVEIYCTRPNNNTRKSIHV..GPGKTLYATGDIIGDIRQAHCNLSRAKWNDTLKQIVIKLRKQF.RNRTI**

**H18969.12.10H3_CrNA**  **AKTIIVQLNESVEIYCTRPNNNTRKSIHV..GPGKTLYATGDIIGDIGQAHCNLSRAKWNDTLKQIVIKLRKQF.RNRTI**

**H19329.32.C9_Non-CrNA**  **TKTIIVQLRESVEINCTRPNNNTRKSIHI..GPGRAFYATGEIIGDIRQAHCNLSRAKWNDTLNQIVIKLRELY.KNKTI**

**H19329.32.E6_Non-CrNA**  **TKTIIVQLRESVEINCTRPNNNTRKSIHI..GPGRAFYATGEIIGDIRQAHCNLSRAKWNDTLNQIVIKLRELY.KNKTI**

**H19329.32.H7_Non-CrNA**  **TKTIIVQLRESVEINCTRPNNNTRKSIHI..GPGRAFYATGEITGDIRQAHCNLSRAKWNDTLNQIVIKLRELY.KNKTI**

**H19329.32.F1_Non-CrNA**  **TKTIIVQLRESVEINCTRPNNNTRKSIHI..GPGRAFYATGEITGDIRQAHCNLSRAKWNDTLNQIVIKLRELY.KNKTI**

**H19329.32.H5_Non-CrNA**  **TKTIIVQLRESVEINCTRPNNNTRKSIHI..GPGRAFYATGEITGDIRQAHCNLSRAKWNDTLNQIVIKLRELY.KNKTI**

**H19329.32.H9_Non-CrNA**  **TKTIIVQLRESVEINCTRPNNNTRKSIHI..GPGRAFYATGEITGDIRQAHCNLSRAKWNDTLNQIVIKLRELY.KNKTI**

**H19329.13.F12_Non-CrNA**  **TKTIIVQLRESVEINCTRPNNNTRKSIHI..GPGRAFYATGEITGDIRQAHCNLSRAKWNDTLNQIVIKLRELY.KNKTI**

**H18887.21.G2_Non-CrNA**  **AKTIIVQLNESVAINCTRPSNNTRKSIPI..GPGRAFYITGEIIGDIRQAHCNISSKNWNKTLEQIVEKLREQFGKNKTI**

**H18887.21.roD7_Non-CrNA** **AKTIIVQLNESVAINCTRPSNNTRKSIPI..GPGRALYTTGEIIGDIRQAHCNISRKNWNKTLEQIVEKLREQFGKNKTI**

**H19861.19.C10_Non-CrNA**  **AKTIIVQLNESVEINCIRPNNNTRKSINI..GPGRAFYTTGEIIGDIRQAHCNISGAKWNSTLKMIVEKLREQYG.NKTI**

**H19861.19.F2_Non-CrNA**  **AKTIIVQLNESVEINCIRPNNNTRKSINI..GPGRAFYTTGEIIGDIRQAHCNISGAKWNSTLKMIVEKLREQFG.NKTI**

**H19861.19.A6_Non-CrNA**  **AKTIIVQLNESVEINCIRPNNNTRKSINI..GPGRAFYTTGEIIGDIRQAHCNISGAKWNSTLKMIVEKLREQFG.NKTI**

**H19489.8.G5_Non-CrNA**  **AKVIIVQLNESVVINCTRPNNNTRKSIHI..GPGRAFYTTGAIIGDIRQAHCNISRVKWENTLKQIVTKLREQF.KNKTI**

**H19489.8.1E10_Non-CrNA**  **AKVIIVQLNESVVINCTRPNNNTRKSIHI..GPGRAFYTTGAIIGDIRQAHCNISRVKWKNTLKQIVTKLREQF.KNKTI**

**H19489.8.1A11_Non-CrNA**  **AKVIIVQLNESVVINCTRPNNNTRKSIHI..GPGRAFYTTGAIIGDIRQAHCNISRVKWKNTLKQIVTKLREQF.KNKTI**

**H19489.8.1H10_Non-CrNA**  **AKVIIVQLNESVVINCTRPNNNTRKSIHI..GPGRALYTTGAIIGDIRQAHCNISRVKWKNTLKQIVTKLREQF.KNKTI**

**H19489.8.2A3_Non-CrNA**  **AKVIIVQLNESVVINCTRPNNNTRKSIHI..GPGRAFYTTGAIIGDIRQAHCNISRVKWKNTLKQIVTKLREQF.KNKTI**

**H19974.11.E12_Non-CrNA**  **AKTIIVQLNESVEINCTRPNNNTRKSIHI..GPGRAFYTTGQIIGNIRQAHCNISRAKWNNTLHKIVKKLREQF.RNKTI**

**H19974.11.E11_Non-CrNA**  **AKTIIVQLNESVEINCTRPNNNTRKSIHI..GPGRAFYTTGQIIGNIRQAHCNISRAKWNNTLHKIVKKLREQF.RNKTI**

**H19792.9.F6_Non-CrNA**  **AKTIIVQLNESIAINCTRPNNNTRKSIHI..GPGRAFYTTGQIIGDIRQAYCNLSREKWDNTLGQIVTKLKEQFG.NKTI**

**H19792.9.B1_Non-CrNA**  **AKTIIVQLNESIAINCTRPNNNTRKSIHI..GPGRAFYTTGQIIGDIRQAYCNLSREKWDNTLEQIVTKLKEQFG.NKTI**

**H19792.9.F1_Non-CrNA**  **AKTIIVQLNESIAINCTRPNNNTRKSIHI..GPGRAFYTTGQIIGDIRQAYCNLSREKWDNTLKQIVTKLKEQFG.NKTI**

**H19792.9.D6_Non-CrNA**  **AKTIIVQLNESIAINCTRPNNNTRKSIHI..GPGRAFYTTGQIIGDIRQAYCNLSREKWDNTLEQIVTKLKEQFG.NKTI**

**H19792.9.C10_Non-CrNA**  **AKTIIVQLNESIAINCTRPNNNTRKSIHI..GPGRAFYTTGQIIGDIRQAYCNLSREKWDNTLEQIVTKLKEQFG.NKTI**

**H18880.10.20_Non-CrNA**  **TKTIIVQLNESVKIDCIRPNNNTRKGIHI..GPGRAFYTTGEIIGDIRQAHCNLSKAQWNNTLKQIVIKLREQF.RNKTI**

**H18880.10.21_Non-CrNA**  **TKTIIVQLNESVKIDCIRPNNNTRKSIHI..GPGRAFYTTGEIIGDIRQAHCNLSKAQWNNTLKQIVIKLREQF.ENKTI**

**H19961.14.F10_Non-CrNA**  **AKTIIVQLNESVEINCTRPNNNTRRSISI..GPGRAFYTTGEIIGNIRQAHCNISRAKWNNTLQQIVNKLREKF.ENKTI**

**H19961.14.E8_Non-CrNA**  **AKTIIVQLNESVEINCTRPNNNTRRSISI..GPGRAFYTTGEIIGNIRQAHCNISRAKWNNTLQQIVNKLREKF.ENKTI**

**H19961.14.G4_Non-CrNA**  **AKTIIVQLNESVEINCTRPNNNTRRSISI..GPGRAFYTTGEIIGNIRQAHCNISRAKWNNTLQQIVNKLREKF.ENKTI**

**H19961.14.B10_Non-CrNA**  **AKTIIVQLNESVEINCTRPNNNTRRSISI..GPGRAFYTTGEIIGNIRQAHCNISRAKWNNTLQQIVNKLREKF.ENKTI**

**H19961.14.F9_Non-CrNA**  **AKTIIVQLNESVEINCTRPNNNTRRSISI..GPGRAFYTTGEIIGNIRQAHCNISRAKWNNTLQQIVNKLREKF.ENKTI**

**H19576.9.H1_Non-CrNA**  **AKTIIVQLKEAVEINCTRPNNNTRKGIHI..GPGRAFYTTGQITGNIRLAHCNISRAKWNNTLQQIVKKLKEQF.NNNTI**

**H19576.9.F4_Non-CrNA**  **AKTIIVQLKEAVEINCTRPNNNTRKSIHI..GPGRAFYTTGEIIGKIRQAHCNISRAKWNNTLQQIVRKLREQF.ENKTI**

410 420 430 440 450 460 470 480

....|....|....|....|....|....|....|....|....|....|....|....|....|....|....|....|

**HXB2/1-856**  **IFKQSSGGDPEIVTHSFNCGGEFFYCNSTQLFNSTW.....FNSTWST......E....GSNNT.EGS......DTITLP**

**H18818.6.1D2_CrNA**  **VFNQSSGGDPEIVMHSFNCGGEFFYCNTTKLFNSTW..NDT.............D..IRG.NNT.EGN......DTITIP**

**H18818.6.1C3_CrNA**  **VFNQSSGGDPEIVMHSFNCGGEFFYCNTTKLFNSTW..NDT.............D..IRG.NNT.EGN......DTITIP**

**H18818.6.1A6_CrNA**  **VFNQSSGGDPEIVMHSFNCGGEFFYCNTTKLFNSTW..NDT.............D..IRG.NNT.EGN......DTITIP**

**H18818.6.1G12_CrNA**  **VFNQSSGGDPEIVMHSFNCGGEFFYCNTTKLFNSTW..NDT.............D..IRG.NNT.EGN......DTITIP**

**H19829.11.H5_CrNA**  **VFNQSSGGDPEIVMHSFNCGGEFFYCNTTKLFNSTW..NDT.............D..IRG.NNN.EGN......DTITLP**

**H19829.11.A2_CrNA**  **VFNQSSGGDPEIVMHSFNCGGEFFYCNTTELFNSTW..NDT.............D..IRG.NNT.EGN......DTITLQ**

**H19829.11.E8_CrNA**  **VFNQSSGGDPEIVMHSFNCGGEFFYCNTTELFNSTW..NDT.............D..IRG.NNT.EGN......DTITLP**

**H19829.11.B4_CrNA**  **VFNQSSGGDPEIVMHSFNCGGEFFYCNTTELFNSTW..NDT.............D.......N..EGN......DTITIP**

**H19829.11.A4_CrNA**  **VFNQSSGGDPEIVMHSFNCGGEFFYCNTTELFNSTW..NDT.............D..IRG.NNT.EGN......DTITIP**

**H19829.11.G8_CrNA**  **VFNQSSGGDPEIVMHSFNCGGEFFYCNTTELFNSTW..NDT.............D..IRG.NNT.EGN......DTITLP**

**H19999.7.1G10_CrNA**  **VFNQSSGGDPEITMHSFNCGGEFFYCNTTQLFNSTW......NSTRNG....T.E..V..SNKT.EI.........ITLP**

**H19999.7.2G7_CrNA**  **VFNQSSGGDPEITMHSFNCGGEFFYCNTTQLFNSTW......NSTRNG....T.E..V..SNKT.EI.........ITLP**

**H19999.7.2D5_CrNA**  **VFNQSSGGDPEITMHSFNCGGEFFYCNTTQLFNSTW......NSTRNG....T.E..V..SNKT.EI.........ITLP**

**H19999.7.1B2_CrNA**  **AFNQSSGGDPEITMHSFNCGGEFFYCNTTQLFNSTWNSNSTWNSTWNG....T.E..A..SN...D.T........ITLP**

**H19999.7.1D2_CrNA**  **AFNQSSGGDPEITMHSFNCGGEFFYCNTTQLFNSTW.........NST....T.E..M..SNKT.E.N........ITLP**

**H19507.18.G11_CrNA**  **VFNQSSGGDPEIIMHTFNCGGEFFYCNSTPLFNSTWNWNDTTGP.WN.......D.T..GSNN.............ITLQ**

**H19507.18.C11_CrNA**  **VFNHSSGGDPEIIMHTFNCGGEFFYCNSTPLFNSTW..NDTTGP.WN.......E.TA.GSNN.............ITLQ**

**H19507.18.A11_CrNA**  **VFNHSSGGDPEIIMHTFNCGGEFFYCNSTPLFNSTW..NDTTGP.WN.......E.TA.GSNN.............ITLQ**

**H19507.18.F4_CrNA**  **VFNHSSGGDPEIIMHTFNCGGEFFYCNSTPLFNSTW..NDTTGP.WN.......E.TA.GSNN.............ITLQ**

**H19793.13.F8_CrNA**  **VFNQSSGGDPEIVTHSFNCGGEFFYCNSTQLFNSTW....LFNSTWNG....N.E....GLNNTQE.N........ITLP**

**H19463.8.A11_CrNA**  **VFNQSSGGDPEIVMHNFNCGGEFFYCNSTQLFNSTW...NANGT.WSG....T.E....GSNNT............FTLP**

**H19463.8.E10_CrNA**  **VFNQSSGGDPEIVMHNFNCGGEFFYCNSTQLFNSTW...NANGT.WSG....T.E....GSNNT............FTLP**

**H19474.17.1G12_CrNA**  **VFNQSSGGDPEIVMHSFNCGGEFFYCDTTQLFNSTW....VT.....G......D..NSNSTK...GN......DTLTLP**

**H19474.17.2H8_CrNA**  **VFNQSSGGDPEIVMHSFNCGGEFFYCDTTQLFNSTW....VT.....G......D..NSNSTK...GN......DTLTLP**

**H18814.10.1E1_CrNA**  **VFNQSSGGDPEIVMHSFNCGGEFFYCNSTQLFNSTWNGTEVT...WNV.....TE....GS.K...GN......DTITLP**

**H18814.10.1B4_CrNA**  **VFNQSSGGDPEIVMHSFNCGGEFFYCNSTQLFNSTWYGTEVT...WNG.....TE....GS.K...GN......DTITLP**

**H18814.10.1C5_CrNA**  **VFNQSSGGDPEIVMHSFNCGGEFFYCNSTQLFNSTWNGTEVT...WNV.....TE....GLNRT.EGN......DTITLP**

**H18814.10.1B1_CrNA**  **VFNQSSGGDPEIVMHSFNCGGEFFYCNSTQLFNSTWNGTEVT...WNV.....TE....GLNRT.EGN......DTITLP**

**H18814.10.1G2_CrNA**  **VFNQSSGGDPEIVMHSFNCGGEFFYCNSTQLFNSTWNGTEVT...WNV.....TE....GLNRT.EGN......DTITLP**

**H11668.12.F11(A)_CrNA**  **VFNQSSGGDPEIVMHSFNCGGEFFYCNSTKLFNSTW.........Y.GN...KTE....GLNNT.DGN......DTLTLP**

**H11668.12.C3_CrNA**  **VFKQSSGGDPEIVMHSFNCGGEFFYCNSTKLFNSTW............N...ETE....GLNNT.DGN......DTLTLP**

**H11668.12.D11_CrNA**  **VFKQSSGGDPEIVMHSFNCGGEFFYCNSTKLFNSTW.........Y.RN...ETE....GLNNT.DGN......DTLTLP**

**H11668.12.H9_CrNA**  **VFNQSSGGDPEIVMHSFNCGGEFFYCNSTKLFNSTW.........Y.GN.....E..TKGLNNT.DGN......DTLTLP**

**H11668.12.E10_CrNA**  **VFNQSSGGDPEIVMHSFNCGGEFFYCNSTKLFNSTW.........Y.GN.....E..TKGLNNT.DGN......DTLTLP**

**H19308.26.B1_CrNA**  **IFNQSSGGDPEIVMHSFICGGEFFYCNTSQLFGSIW..........N.......D.STRNDTR..ESNS...T.EPIILP**

**H19308.26.F8I_CrNA**  **IFNQSSGGDPEIVMHSFICGGEFFYCNTSQLFGSIW..........N.......D.STRNDTR..ESNS...T.EPIILP**

**H19308.26.D8_CrNA**  **IFNQSSGGDPEIVMHSFICGGEFFYCNTSQLFDSIW..NDST...WN.......D......TR..ESNS...T.EPIILP**

**H19308.26.D1_CrNA**  **IFNQSSGGDPEIVMHSFICGGEFFYCNTSQLFDSIW..NDST...WN.......D......TR..ESNS...T.EPIILP**

**H19308.26.E4_CrNA**  **IFNQSSGGDPEIVMHSFICGGEFFYCNTSQLFDSIW..NDST...WN.......D......TR..ESNS...T.EPIILP**

**H19885.31.D9_CrNA**  **VFNQSSGGDPEIEMHSFNCRGEFFYCNTTKLFNSTWNSAG.NNSVW...............NNN.DP.......DIITLP**

**H19885.31.G12_CrNA**  **VFNQSSGGDPEIEMHSFNCRGEFFYCNTTKLFNSTWNSTG.NNSVW...............NNN.DP.......DIITLP**

**H19885.31.E2_CrNA**  **IFNQSSGGDPEIEMHSFNCRGEFFYCNTTKLFNSTWNSTG.NNSVW...............NNN.DP.......DIITLP**

**H19885.31.G10_CrNA**  **VFNQSSGGDPEIEMHSFNCRGEFFYCNTTKLFNSTWNSTG.NNSVW...............NNN.EP.......DIITLP**

**H19885.31.H11_CrNA**  **VFNQSSGGDPEIEMHSFNCRGEFFYCNTTKLFNSTWNSTG.NNSVW...............NNN.DP.......DIITLP**

**H19885.31.A5_CrNA**  **VFNQSSGGDPEIEMHSFNCRGEFFYCNTTKLFNSTWNSTG.NNSVW...............NNN.DP.......DIITLP**

**H19885.31.F8_CrNA**  **VFNQSSGGDPEIEMHSFNCRGEFFYCNTTKLFNSTWNSTG.NNSVW...............NNN.DP.......DIITLP**

**H19885.31.H10_CrNA**  **VFNQSSGGDPEIEMHSFNCRGEFFYCNTTKLFNSTWNSTG.NNSVW...............NNN.DP.......DIITLP**

**H19885.31.G1_CrNA**  **VFNQSSGGDPEIEMHSFNCRGEFFYCNTTKLFNSTWNSTG.NNSVW...............NNN.DP.......DIITLP**

**H19885.31.C6_CrNA**  **VFNQSSGGDPEIEMHSFNCRGEFFYCNTTKLFNSTWNSTG.NNSVW...............NNN.DP.......DIITLP**

**H19885.31.E11_CrNA**  **VFNQSSGGDPEIEMHSFNCRGEFFYCNTTKLFNSTWNSTG.NNSVW...............NNN.DP.......DIITLP**

**H18969.12.9D9_CrNA**  **VFTKSSGGDPEIVMHSFNCGGEFFYCNSTQLFNSTW...MLNST.W........E.....SNST.E........ENITLP**

**H18969.12.8E6_CrNA**  **VFTQSSGGDPEIVMHSFNCGGEFFYCNSTQLFNSTW...MLNST.W........E.....SNST.E........ENITLP**

**H18969.12.8G8_CrNA**  **VFTQSSGGDPEIVMHSFNCGGEFFYCNSTQLFNSTW...MLNST.W........E.....SNST.E........ENITLP**

**H18969.12.6D7_CrNA**  **VFTQSSGGDPEIVMHSFNCGGEFFYCNSTQLFNSTW...MLNST.W........E.....SNST.E........ENITLP**

**H18969.12.8B4_CrNA**  **VFTQSSGGDPEIVMHSFNCGGEFFYCNSTQLFNSTW...MLNST.W........E.....SNST.E........ENITLP**

**H18969.12.7D5_CrNA**  **VFTQSSGGDPEIVMHSFNCGGEFFYCNSTQLFNSTW...MLNST.W........E.....SNST.E........ENITLP**

**H18969.12.6C4_CrNA**  **VFTQSSGGDPEIVMHSFNCGGEFFYCNSTQ.........MLNST.W........E.....SNST.E........ENITLP**

**H18969.12.10H3_CrNA**  **VFTQSSGGDPEIVMHSFNCGGEFFYCNSTQLFNSTW...MLNST.W........E.....SNST.E........ENITLP**

**H19329.32.C9_Non-CrNA**  **VFNPSSGGDPEIVMHSFNCGGEFFYCNTTQLFNSTW...DV..NA.TG..........NG..TT.E.SNS....T.ITLS**

**H19329.32.E6_Non-CrNA**  **VFNPSSGGDPEIVMHSFNCGGEFFYCNTTQLFNSTW...DV..NA.TG..........NG..TT.E.SNS....T.ITLS**

**H19329.32.H7_Non-CrNA**  **VFNPSSGGDPEIVMHSFNCGGEFFYCNTTQLFNSTW...DV..NA.TG..........NG..TT.E.SNS....T.ITLS**

**H19329.32.F1_Non-CrNA**  **VFNPSSGGDPEIVMHSFNCGGEFFYCNTTQLFNSTW...DV..NA.TG..........NG..TT.E.SNS....T.ITLS**

**H19329.32.H5_Non-CrNA**  **VFNPSSGGDPEIVMHSFNCGGEFFYCNTTQLFNSTW...DV..NA.TG..........NG..TT.E.SNS....T.ITLS**

**H19329.32.H9_Non-CrNA**  **VFNPSSGGDPEIVMHSFNCGGEFFYCNTTQLFNSTW...DV..NA.TG..........NG..TT.E.SNS....T.ITLS**

**H19329.13.F12_Non-CrNA**  **VFNPSSGGDPEIVMHSFNCGGEFFYCNTTQLFNSTW...DV..NA.TG..........NG..TT.E.SNS....T.ITLS**

**H18887.21.G2_Non-CrNA**  **AFNQASGGDPEIVMHTFNCGGEFFYCNTTRLFNSTW...YANSTR...........NATG......SNDT....DPITLP**

**H18887.21.roD7_Non-CrNA** **AFNQASGGDPEIVMHTFNCGGEFFYCNTTRLFNSTW...YANST.W..........NATG......SNDT....DPITLP**

**H19861.19.C10_Non-CrNA**  **NFNQSSGGDPEIVMHSFNCGGEFFYCNTTKLFNSTW...NMNDTR.........ESNNTG.......QN.......ITLP**

**H19861.19.F2_Non-CrNA**  **NFNQSSGGDPEIVMHSFNCGGEFFYCNTTKLFNSTW...NMNDTR.........ESNNTG.......QN.......ITLP**

**H19861.19.A6_Non-CrNA**  **NFNQSSGGDPEIVMHSFNCGGEFFYCNTTKLFNSTW...NMNDTR.........ESNNTG.......QN.......ITLP**

**H19489.8.G5_Non-CrNA**  **AFNQSSGGDPEIVMHSFNCGGEFFYCDSTQLFNSTW.....NST..EG....TND.T........EG.ENNT..D.ITLP**

**H19489.8.1E10_Non-CrNA**  **AFNQSSGGDPEIVMHSFNCGGEFFYCDSTQLFNSTW.....NST..EG....TND.T........EG.ENNT..D.ITLP**

**H19489.8.1A11_Non-CrNA**  **AFNQSSGGDPEIVMHSFNCGGEFFYCDSTQLFNSTW.....NST..EG....TND.T........EG.ENNT..D.ITLP**

**H19489.8.1H10_Non-CrNA**  **AFNQSSGGDPEIVMHSFNCGGEFFYCDSTQLFNSTW.....NST..EG....TND.T........EG.ENNT..D.ITLP**

**H19489.8.2A3_Non-CrNA**  **AFNQSSGGDPEIVMHSFNCGGEFFYCDSTQLFNSTW.....NST..EG....TND.T........EG.ENNT..D.ITLP**

**H19974.11.E12_Non-CrNA**  **VFKQSSGGDPEIVMHSFNCGGEFFYCNSTQLFNSTW...Y.G..NK......SSD..NTGE....EGN........ITLP**

**H19974.11.E11_Non-CrNA**  **VFKQSSGGDPEIVMHSFNCGGEFFYCNSTQLFNSTW...Y.G..N.E.....SSD..NPGV....EGN........ITLP**

**H19792.9.F6_Non-CrNA**  **IFNQSSGGDPEIVMHSFNCGGEFFYCNTTQLFNSTW......NSTRNG...TSND...K...H.............IILP**

**H19792.9.B1_Non-CrNA**  **IFNQSSGGDPEIVMHSFNCGGEFFYCNTTQLFNSTW......NSTRNS...TSND...K...H.............IILP**

**H19792.9.F1_Non-CrNA**  **IFNQSSGGDPEIVMHSFNCGGEFFYCNTTQLFNSTW......NSTRNG...TSND...K...H.............IILP**

**H19792.9.D6_Non-CrNA**  **IFNQSSGGDPEIVMHSFNCGGEFFYCNTTQLFNSTW......NSTRNG...TSND...E...H.............IILP**

**H19792.9.C10_Non-CrNA**  **IFNQSSGGDPEIVMHSFNCGGEFFYCNTTQLFNSTW......NSTRNG...TSND...K...H.............IILP**

**H18880.10.20_Non-CrNA**  **VFNQSSGGDPEIVMHSFNCGGEFFYCNSTALFNSTW...STGNDTWS....TVND.TNK....T.D.N........ITLP**

**H18880.10.21_Non-CrNA**  **VFNQSSGGDPEIVMHSFNCGGEFFYCNSTALFNSTW...STGNDTWS....TVND.TNK....T.D.N........ITLP**

**H19961.14.F10_Non-CrNA**  **VFNQSSGGDPEIVMHSFNCGGEFFYCNTTQLFNSTW......NNTG.............GSNNT.EGNNT......ITLP**

**H19961.14.E8_Non-CrNA**  **VFNQSSGGDPEIVMHSFNCGGEFFYCNTTQLFNSTW..NDTG.................GSNNT.EGNNT......ITLP**

**H19961.14.G4_Non-CrNA**  **VFNQPSGGDPEIVMHSFNCGGEFFYCNTTQLFNSTW..NDTG.................GSNNT.EGSNT......ITLP**

**H19961.14.B10_Non-CrNA**  **VFNQSSGGDPEIVMHSFNCGGEFFYCNTTQLFNSTW..NDTG.................GSNNT.EGNNT......ITLP**

**H19961.14.F9_Non-CrNA**  **VFNQPSGGDPEIVMHSFNCGGEFFYCNTTQLFNSTW..NDTG.................GSNNT.EGNNT......ITLP**

**H19576.9.H1_Non-CrNA**  **VFNHSSGGDPEIVTHSFNCGGEFFYCNTTQLFNSTWNNTG.G..SWNVT..........G...N.D.T........ITLP**

**H19576.9.F4_Non-CrNA**  **VFNHSSGGDPEIVTHSFNCGGEFFYCDTTQLFNSTWNSTD.G..SWNDT................EGSWNVTGNDTITLP**

490 500 510 520 530 540 550 560

....|....|....|....|....|....|....|....|....|....|....|....|....|....|....|....|

**HXB2/1-856**  **CRIKQIINMWQKVGKAMYAPPISGQIRCSSNITGLLLTRDGG....NSNN..........ESEIFRPGGGDMRDNWRSEL**

**H18818.6.1D2_CrNA**  **CRIKQIVNMWQEVGKAMYAPPIRGQIRCSSNITGLLLTRDGG....SESNT..........TEIFRPGGGDMRDNWRSEL**

**H18818.6.1C3_CrNA**  **CRIKQIVNMWQEVGKAMYAPPIRGQIRCSSNITGLLLTRDGG....SESNT..........TEIFRPGGGDMRDNWRSEL**

**H18818.6.1A6_CrNA**  **CRIKQIVNMWQEVGKAMYAPPIRGQIRCSSNITGLLLTRDGG....SESNT..........TEIFRPGGGDMRDNWRSEL**

**H18818.6.1G12_CrNA**  **CRIKQIVNMWQEVGKAMYAPPIRGQIRCSSNITGLLLTRDGG....SESNT..........TEIFRPGGGDMRDNWRSEL**

**H19829.11.H5_CrNA**  **CRIKQIVNMWQEVGKAMYAPPIRGQIRCSSNITGLLLTRDGG....SESNT..........TEIFRPGGGDMRDNWRSEL**

**H19829.11.A2_CrNA**  **CRIKQIVNMWQEVGKAMYAPPIRGQIRCSSNITGLLLTRDGG....SESNT..........TEIFRPGGGDMRDNWRSEL**

**H19829.11.E8_CrNA**  **CRIKQIVNMWQEVGKAMYAPPIRGQIRCSSNITGLLLTRDGG....SESNT..........TEIFRPGGGDMRDNWRSEL**

**H19829.11.B4_CrNA**  **CRIKQIINMWQEVGKAMYAPPIRGQIRCSSNITGLLLTRDGG....SESNT..........TEIFRPGGGDMRDNWRSEL**

**H19829.11.A4_CrNA**  **CRIKQIVNMWQEVGKAMYAPPIRGQIRCSSNITGLLLTRDGG....SESNT..........TEIFRPGGGDMRDNWRSEL**

**H19829.11.G8_CrNA**  **CRIKQIINMWQEVGKAMYAPPIRGQIRCSSNITGLLLTRDGG....SESNT..........TEIFRPGGGDMRDNWRSEL**

**H19999.7.1G10_CrNA**  **CRIKQLINMWQEVGKVMYAPPIRGKIRCSSKITGLLLTRDGG.......NNKSE.A..ENETEIFRPGGGDMRDNWRSEL**

**H19999.7.2G7_CrNA**  **CRIKQLINMWQEVGKVMYAPPIRGKIRCTSKITGLLLTRDGG.......NNKSE.A..ENETEIFRPGGGDMRDNWRSEL**

**H19999.7.2D5_CrNA**  **CRIKQLINMWQEVGKVMYAPPIRGKIRCTSKITGLLLTRDGG.......NNKSE.A..ENETEIFRPGGGDMRDNWRSEL**

**H19999.7.1B2_CrNA**  **CRIKQIINMWQEVGKVMYAPPIRGTIKCSSNITGLLLTRDGG.......NNK...NGTENETEIFRPGGGDMRDNWRSEL**

**H19999.7.1D2_CrNA**  **CRIKQLINMWQEVGKVMYAPPIRGTIRCSSNITGLLLTRDGG.......NNKSK.D..ENGTEIFRPGGGDMRDNWRSEL**

**H19507.18.G11_CrNA**  **CRIKQIINRWQEVGKAMYAPPIKGQIRCSSNITGLLLTRDGG.....NNNNK.........TEIFRPGGGDMRDNWRSEL**

**H19507.18.C11_CrNA**  **CRIKQIINRWQEVGKAMYAPPIKGQIRCSSKITGLLLTRDGG.NNTTE.............TEIFRPGGGDMRDNWRSEL**

**H19507.18.A11_CrNA**  **CRIKQIINRWQEVGKAMYAPPIKGQIRCSSKITGLLLTRDGG.NNTTE.............TEIFRPGGGDMRDNWRSEL**

**H19507.18.F4_CrNA**  **CRIKQIINRWQEVGKAMYAPPIKGQIRCSSKITGLLLTRDGG.NNTTE.............TEIFRPGGGDMRDNWRSEL**

**H19793.13.F8_CrNA**  **CRIKQIINMWQKVGKAMYAPPIRGQIRCSSNITGLLLTRDGG....ND..NE.........TETFRPGGGDMRENWRSEL**

**H19463.8.A11_CrNA**  **CRIKQIINLWQEVGKAMYAPPIRGQIRCSSNITGLLLIRDGG.....DNNSE.........TETFRPGGGNMKDNWRSEL**

**H19463.8.E10_CrNA**  **CRIKQIINLWQEVGKAMYAPPIRGQIRCSSNITGLLLIRDGG.....DNNSE.........TETFRPGGGNMKDNWRSEL**

**H19474.17.1G12_CrNA**  **CRIKQIINRWQEVGKAMYAPPIRGQIRCSSNITGLLLTRDGG.........NS...PNN..TEIFRPGGGDMRDNWRSEL**

**H19474.17.2H8_CrNA**  **CRIKQIINRWQEVGKAMYAPPIRGQIRCSSNITGLLLTRDGG.........NS...PNN..TEIFRPGGGDMRDNWRSEL**

**H18814.10.1E1_CrNA**  **CRIKQIINLWQEVGKAMYAPPIRGQIRCSSNITGLLLTRDGG......NNTN....G....TEIFRPGGGDMRDNWRSEL**

**H18814.10.1B4_CrNA**  **CKIKQIINLWQEVGKAMYAPPIRGQIRCSSNITGLLLTRDGG......NNTN....G....TEIFRPGGGDMRDNWRSEL**

**H18814.10.1C5_CrNA**  **CRIKQIINLWQEVGKAMYAPPIRGQIRCSSNITGLLLTRDGG......NNTN....G....TEIFRPGGGDMRDNWRSEL**

**H18814.10.1B1_CrNA**  **CRIKQIINLWQEVGKAMYAPPIRGQIRCSSNITGLLLTRDGG......NNTN....G....TEIFRPGGGDMRDNWRSEL**

**H18814.10.1G2_CrNA**  **CRIKQIINLWQEVGKAMYAPPIRGQIRCSSNITGLLLTRDGG......NNTN....G....TEIFRPGGGDMRDNWRSEL**

**H11668.12.F11(A)_CrNA**  **CRIKQIINLWQEVGKAMYAPPIAGQIRCSSNITGLLLTRDGGNNTSNNS..K....P.....EIFRPGGGDMRDNWRSEL**

**H11668.12.C3_CrNA**  **CRIKQIINLWQEVGKAMYAPPIAGQIRCSSNITGLLLTRDGGNNTSNES..E....P.....EIFRPGGGDMRDNWRSEL**

**H11668.12.D11_CrNA**  **CRIKQIINLWQEVGKAMYAPPIAGQIRCSSNITGLLLTRDGGNNTSNS...E.........LEIFRPGGGDMRDNWRSEL**

**H11668.12.H9_CrNA**  **CRIKQIINLWQEVGKAMYAPPIAGQIRCSSNITGLLLTRDGGNNTSNES..K....P.....EIFRPGGGDMRDNWRSEL**

**H11668.12.E10_CrNA**  **CRIKQIINLWQEVGKAMYAPPIAGQIRCSSNITGLLLTRDGGNNTSNES..K....P.....EIFRPGGGDMRDNWRSEL**

**H19308.26.B1_CrNA**  **CRIKQIINRWQEVGKAMYAPPIRGQIRCSSNITGLLLTRDGG...NSDNTTE.....NTT.TETFRPGGGNMRDNWRSEL**

**H19308.26.F8I_CrNA**  **CRIKQIINRWQEVGKAMYAPPIRGQIRCSSNITGLLLTRDGG...NSDNTTE.....NTT.TETFRPGGGNMRDNWRSEL**

**H19308.26.D8_CrNA**  **CRIKQIINRWQEVGKAMYAPPIRGQIRCSSNITGLLLTRDGG...NNDNTTKNT.......TETFRPGGGDMRDNWRSEL**

**H19308.26.D1_CrNA**  **CRIKQIINRWQEVGKAMYAPPIRGQIRCSSNITGLLLTRDGG...NSDNTTKNT.......TETFRPGGGDMRDNWRSEL**

**H19308.26.E4_CrNA**  **CRIKQIINRWQEVGKAMYAPPIRGQIRCSSNITGLLLTRDGG...NSDNTTKNT.......TETFRPGGGDMRDNWRSEL**

**H19885.31.D9_CrNA**  **CRIKQIINRWQEVGKAMYAPPIRGNINCSSQITGLLLTRDGG.........KEDNATNTTEIEIFRPGGGDMRDNWRSEL**

**H19885.31.G12_CrNA**  **CRIKQIINRWQEVGKAMYAPPIRGNINCSSQITGLILTRDGG.........KEDNDTNTTEIEIFRPGGGDMRDNWRSEL**

**H19885.31.E2_CrNA**  **CRIKQIINRWQEVGKAMYAPPIRGNINCSSQITGLLLTRDGG.........KEDNATNTTEIEIFRPGGGDMRDNWRSEL**

**H19885.31.G10_CrNA**  **CRIKQIINRWQEVGKAMYAPPIRGNINCSSQITGLLLTRDGG.........KEDNATNTTEIEIFRPGGGDMRDNWRSEL**

**H19885.31.H11_CrNA**  **CRIKQIINRWQEVGKAMYAPPIRGNINCSSQITGLLLTRDGG.........KEDNATNTTEIEIFRPGGGDMRDNWRSEL**

**H19885.31.A5_CrNA**  **CRIKQIINRWQEVGKAMYAPPIRGNINCSSQITGLLLTRDGG.........KEDNATNTTEIEIFRPGGGDMRDNWRSEL**

**H19885.31.F8_CrNA**  **CRIKQIINRWQEVGKAMYAPPIRGNINCSSQITGLLLTRDGG.........KEDNATNTIEIEIFRPGGGDMRDNWRSEL**

**H19885.31.H10_CrNA**  **CRIKQIINRWQEVGKAMYAPPIRGNINCSSQITGLLLTRDGG.........KEDNATNTTEIEIFRPGGGDMRDNWRSEL**

**H19885.31.G1_CrNA**  **CRIKQIINRWQEVGKAMYAPPIRGNINCSSQITGLLLTRDGG.........KEDNATNTTEIEIFRPGGGDMRDNWRSEL**

**H19885.31.C6_CrNA**  **CRIKQIINRWQEVGKAMYAPPIRGNINCSSQITGLLLTRDGG.........KEDNATNTTEIEIFRPGGGDMRDNWRSEL**

**H19885.31.E11_CrNA**  **CRIKQIINRWQEVGKAMYAPPIRGNINCSSQITGLLLTRDGG.........KEDNATNTTEIEIFRPGGGDMRDNWRSEL**

**H18969.12.9D9_CrNA**  **CRIKQIINMWQEVGKAMYAPPISGQIRCSSNITGLLLTRDGG.....NNTN.....G....TEIFRPGGGDMRDNWRSEL**

**H18969.12.8E6_CrNA**  **CRIKQIINMWQEVGKAMYAPPISGQIRCSSNITGLLLTRDGG.....NNTN.....G....TEIFRPGGGDMRDNWRSEL**

**H18969.12.8G8_CrNA**  **CRIKQIINMWQEVGKAMYAPPISGQIRCSSNITGLLLTRDGG.....NNTN.....G....TEIFRPGGGDMGDKWRSEL**

**H18969.12.6D7_CrNA**  **CRIKQIINMWQEVGKAMYAPPISGQIRCSSNITGLILTRDGG.....NNTN.....G....TEIFRPGGGDMRDNWRSEL**

**H18969.12.8B4_CrNA**  **CRIKQIINMWQEVGKAMYAPPISGQIRCSSNITGLLLTRDGG.....NNTN.....G....TEIFRPGGGDMRDNWRSEL**

**H18969.12.7D5_CrNA**  **CRIKQIINMWQEVGKAMYAPPISGQIRCSSNITGLILTRDGG.....NNTN.....G....TEIFRPGGGDMRDNWRSEL**

**H18969.12.6C4_CrNA**  **CRIKQIINMWQEVGKAMYAPPISGQIRCSSNITGLLLTRDGG.....NNTN.....G....TEIFRPGGGDMRDNWRNEL**

**H18969.12.10H3_CrNA**  **CRIKQIINMWQEVGKAMYAPPISGQIRCSSNITGLLLTRDGG.....NNTN.....G....TEIFRPGGGDMRDNWRNEL**

**H19329.32.C9_Non-CrNA**  **CRIKQIINRWQEVGKAMYAPPIKGQISCSSNITGLLLTRDGG.........KNESN.....TEIFRPGGGDMRDNWRSEL**

**H19329.32.E6_Non-CrNA**  **CRIKQIINRWQEVGKAMYAPPIEGQISCSSNITGLLLTRDGG.........KNESN.....TEIFRPGGGDMRDNWRSEL**

**H19329.32.H7_Non-CrNA**  **CRIKQIINRWQEVGKAMYAPPIKGQISCSSNITGLLLTRDGG.........KNESN.....TEIFRPGGGDMRDNWRSEL**

**H19329.32.F1_Non-CrNA**  **CRIKQIINRWQEVGKAMYAPPIKGQISCSSNITGLLLTRDGG.........KNESN.....TEIFRPGGGDMRDNWRSEL**

**H19329.32.H5_Non-CrNA**  **CRIKQIINRWQEVGKAMYAPPIKGQISCSSNITGLLLTRDGG.........KNESN.....TEIFRPGGGDMRDNWRSEL**

**H19329.32.H9_Non-CrNA**  **CRIKQIINRWQEVGKAMYAPPIEGQISCSSNITGLLLTRDGG.........KNESN.....TEIFRPGGGDMRDNWRSEL**

**H19329.13.F12_Non-CrNA**  **CRIKQIINRWQEVGKAMYAPPIEGQISCSSNITGLLLTRDGG.........KNESN.....TEIFRPGGGDMRDNWRSEL**

**H18887.21.G2_Non-CrNA**  **CRIKQIINRWQEVGKAMYAPPIRGQIRCSSNITGLLLTRDGD................NNETETFRPGGGDMRDNWRSEL**

**H18887.21.roD7_Non-CrNA** **CRIKQIINRWQEVGKAMYAPPIRGQIRCSSNITGLLLTRDGD................NNETETFRPGGGDMRDNWRSEL**

**H19861.19.C10_Non-CrNA**  **CRIKQIINMWQEVGKAMYAPPIKGQIRCSSNITGLLLIRDGG............TNG.TNETEIFRPGGGDMRDNWRSEL**

**H19861.19.F2_Non-CrNA**  **CRIKQIINMWQEVGKAMYAPPIKGQIRCSSNITGLLLIRDGG............TNG.TNETEIFRPGGGDMRDNWRSEL**

**H19861.19.A6_Non-CrNA**  **CRIKQIINMWQEVGKAMYAPPIKGQIRCSSNITGLLLIRDGG............TNG.TNETEIFRPGGGDMRDNWRSEL**

**H19489.8.G5_Non-CrNA**  **CRIKQIINMWQKVGKAMYAPPIRGQIRCSSNITGLLLTRDGG....NNNNNRDE.......TEIFRPAGGDMRDNWRSEL**

**H19489.8.1E10_Non-CrNA**  **CRIKQIINMWQKVGKAMYAPPIRGQIRCSSNITGLLLTRDGG....NNNNSRNE.......TEIFRPAGGDMRDNWRSEL**

**H19489.8.1A11_Non-CrNA**  **CRIKQIINMWQKVGKAMYAPPIRGQIRCSSNITGLLLTRDGG....NNNNNRNE.......TEIFRPAGGDMRDNWRSEL**

**H19489.8.1H10_Non-CrNA**  **CRIKQIINMWQKVGKAMYAPPIRGQIRCSSNITGLLLTRDGG....NNNNSRNE.......TEIFRPAGGDMRDNWRSEL**

**H19489.8.2A3_Non-CrNA**  **CRIKQIINMWQKVGKAMYAPPIRGQIRCSSNITGLLLTRDGG....NNNNNRNE.......TEIFRPAGGDMRDNWRSEL**

**H19974.11.E12_Non-CrNA**  **CRIKQIINLWQEVGKAMYAPPIGGQIRCSSNITGLLLTRDGG......NNN....I.TET.TEIFRPGGGDMRDNWRSEL**

**H19974.11.E11_Non-CrNA**  **CRIKQIINLWQEVGKAMYAPPIGGQIRCSSNITGLLLTRDGG......NNN....I.T...TEIFRPGGGDMRDNWRSEL**

**H19792.9.F6_Non-CrNA**  **CRIKQIINRWQEVGKAMYAPPIKGQIKCSSNITGLLLTRDGG............NNG..NESETFRPGGGDMRDNWRSEL**

**H19792.9.B1_Non-CrNA**  **CRIKQIINRWQEVGKAMYAPPIKGQIKCSSNITGLLLTRDGG............NNG..NESEIFRPGGGDMRDNWRSEL**

**H19792.9.F1_Non-CrNA**  **CRIKQIINRWQEVGKAMYAPPIKGQIKCSSNITGLLLTRDGG............NNG..NESEIFRPGGGDMRDNWRSEL**

**H19792.9.D6_Non-CrNA**  **CRIKQIINRWQEVGKAMYAPPIKGQIKCSSNITGLLLTRDGG............NNG..NESEIFRPGGGDMRDNWRSEL**

**H19792.9.C10_Non-CrNA**  **CRIKQIINRWQEVGKAMYAPPIKGQIKCSSNITGLLLTRDGG............NNG..NESEIFRPGGGDMRDNWRSEL**

**H18880.10.20_Non-CrNA**  **CRIKQIINMWQEVGKAMYAPPIRGQIRCSSNITGLLLTRDGG........NK..TTG....TEVFRPGGGDMRDNWRSEL**

**H18880.10.21_Non-CrNA**  **CRIKQIINMWQEVGKAMYAPPIRGQIRCSSNITGLLLTRDGG........N...ANG....TEVFRPGGGDMRDNWRSEL**

**H19961.14.F10_Non-CrNA**  **CRIKQIINMWQEVGKAMYAPPIKGQIRCSSNITGLLLTRDGG.........KTTNN.....TEIFRPGGGDMRENWRSEL**

**H19961.14.E8_Non-CrNA**  **CRIKQIINMWQEVGKAMYAPPIKGQIRCSSNITGLLLTRDGG.........KTTNN.....TEIFRPGGGDMRDNWRSEL**

**H19961.14.G4_Non-CrNA**  **CRIKQIINMWQEVGKAMYAPPIKGQIRCSSNITGLLLTRDGG.........KTTNN.....TEIFRPGGGDMRDNWRSEL**

**H19961.14.B10_Non-CrNA**  **CRIKQIINMWQEVGKAMYAPPIKGQIRCSSNITGLLLTRDGG.........KTTNN.....TEIFRPGGGDMRDNWRSEL**

**H19961.14.F9_Non-CrNA**  **CRIKQIINMWQEVGKAMYAPPIKGQIRCSSNITGLLLTRDGG.........KTTNN.....TEIFRPGGGDMRDNWRSEL**

**H19576.9.H1_Non-CrNA**  **CRIKQIINLWQEVGKAMYAPPIRGHIRCSSNITGLLLTRDGG...NGNNTNR.........SETFRPGGGDMRDNWRSEL**

**H19576.9.F4_Non-CrNA**  **CRIKQIINLWQEVGKAMYAPPIRGQIRCSSNITGLLLTRDGG...SGNNM..SE.......FETFRPGGGDMRDNWRSEL**

570 580 590 600 610 620 630 640

....|....|....|....|....|....|....|....|....|....|....|....|....|....|....|....|

**HXB2/1-856**  **YKYKVVKIEPLGVAPTKAKRRVVQREKRAVG.IGALFLGFLGAAGSTMGAASMTLTVQARQLLSGIVQQQNNLLRAIEAQ**

**H18818.6.1D2_CrNA**  **YKYKVVRIEPLGVAPTKAKRRVVQREKRAVGTIGAMFLGFLGAAGSTMGAASITLTVQARLLLSGIVQQQNNLLRAIEAQ**

**H18818.6.1C3_CrNA**  **YKYKVVRIEPLGVAPTKAKRRVVQREKRAVGTIGAMFLGFLGAAGSTMGAASITLTVQARLLLSGIVQQQNNLLRAIEAQ**

**H18818.6.1A6_CrNA**  **YKYKVVRIEPLGVAPTKAKRRVVQREKRAVGTIGAMFLGFLGAAGSTMGAASITLTVQARLLLSGIVQQQNNLLRAIEAQ**

**H18818.6.1G12_CrNA**  **YKYKVVRIEPLGVAPTKAKRRVVQREKRAVGTIGAMFLGFLGAAGSTMGAASITLTVQARLLLSGIVQQQNNLLRAIEAQ**

**H19829.11.H5_CrNA**  **YKYKVVRIEPLGVAPTKAKRRVVQREKRAVGTIGAMFLGFLGAAGSTMGAASITLTVQARLLLSGIVQQQNNLLRAIEAQ**

**H19829.11.A2_CrNA**  **YKYKVVRIEPLGVAPTKAKRRVVQREKRAVGTIGAMFLGFLGAAGSTMGAASITLTVQARLLLSGIVQQQNNLLRAIEAQ**

**H19829.11.E8_CrNA**  **YKYKVVKIEPLGVAPTKAKRRVVQREKRTVGTIGAMFLGFLGAAGSTMGAASITLTVQARLLLSGIVQQQNNLLRAIEAQ**

**H19829.11.B4_CrNA**  **YKYKVVKIEPLGVAPTKAKRRVVQREKRAVGTIGAMFLGFLGAAGSTMGAASITLTVQARLLLSDIVQQQNNLLRAIEAQ**

**H19829.11.A4_CrNA**  **YKYKVVKIEPLGVAPTKAKRRVVQREKRAVGTIGAMFLGFLGAAGSTMGAASITLTVQARLLLSGIVQQQNNLLRAIEAQ**

**H19829.11.G8_CrNA**  **YKYKVVRIEPLGVAPTKAKRRVVQREKRAVGTIGAMFLGFLGAAGSTMGAASITLTVQARLLLSGIVQQQNNLLRAIEAQ**

**H19999.7.1G10_CrNA**  **YKYKVVKIEPLGVAPTKAKRRVVQREKRAVG.LGAMFLGFLGAAGSTMGAASVTLTVQARLLLSGIVQQQNNLLRAIEAQ**

**H19999.7.2G7_CrNA**  **YKYKVVKIEPLGVAPTKAKRRVVQREKRAVG.LGAMFLGFLGAAGSTMGAASVTLTVQARLLLSGIVQQQNNLLRAIEAQ**

**H19999.7.2D5_CrNA**  **YKYKVVKIEPLGVAPTKAKRRVVQREKRAVG.LGAMFLGFLGAAGSTMGAASVTLTVQARLLLSGIVQQQNNLLRAIEAQ**

**H19999.7.1B2_CrNA**  **YKYKVIKIEPLGVAPTKAKRRVVQREKRAVG.IGAMFLGFLGAAGSTMGAASVTLTVQARQLLSGIVQQQNNLLRAIEAQ**

**H19999.7.1D2_CrNA**  **YKYKVVKIEPLGVAPTKAKRRVVQREKRAVG.IGAMFLGFLGAAGSTMGAASVTLTVQARQLLSGIVQQQNNLLRAIKAQ**

**H19507.18.G11_CrNA**  **YKYKVVKIEPLGVAPTKAKRRVVQREKRAVGTIGAMFLGFLGAAGSTMGAASVTLTVQARQLLSGIVQQQNNLLKAIEAQ**

**H19507.18.C11_CrNA**  **YKYKIVKIEPLGVAPTKAKRRVVQREKRAVGTIGAMFLGFLGAAGSTMGAASITLTVQARQLLSGIVQQQNNLLKAIEAQ**

**H19507.18.A11_CrNA**  **YKYKIVKIEPLGVAPTKAKRRVVQREKRAVGTIGAMFLGFLGAAGSTMGAASITLTVQARQLLSGIVQQQNNLLKAIEAQ**

**H19507.18.F4_CrNA**  **YKYKIVKIEPLGVAPTKAKRRVVQREKRAVGTIGAMFLGFLGAAGSTMGAASVTLTVQARQLLSGIVQQQNNLLKAIEAQ**

**H19793.13.F8_CrNA**  **YKYKVVKIEPLGVAPTKAKRRVVQREKRAVG.IGAVFLGFLGAAGSTMGAASMTLTVQARLLLSGIVQQQNNLLRAIEAQ**

**H19463.8.A11_CrNA**  **YKYKVVKIEPLGVAPTKAKRRVVQREKRAVG.IGAVFLGFLGAAGSTMGAAAVTLTVQARLLLSGIVQQQNNLLRAIEAQ**

**H19463.8.E10_CrNA**  **YKYKVVKIEPLGVAPTKAKRRVVQREKRAVG.IGAVFLGFLGAAGSTMGAAAVTLTVQARLLLSGIVQQQNNLLRAIEAQ**

**H19474.17.1G12_CrNA**  **YKYKVVKIEPLGVAPTKAKRRVVQREKRAVG.IGALFLGFLGAAGSTMGAASITLTVQARQLLSGIVQQQSNLLRAIEAQ**

**H19474.17.2H8_CrNA**  **YKYKVVKIEPLGVAPTKAKRRVVQREKRAVG.IGALFLGFLGAAGSTMGAASITLTVQARQLLSGIVQQQSNLLRAIEAQ**

**H18814.10.1E1_CrNA**  **YKYKVVKIEPLGVAPTKAKRRVVQREKRAAG.IGALFLGFLGAAGSTMGAASMTLTVQARQLLSGIVQQQNNLLRAIEAQ**

**H18814.10.1B4_CrNA**  **YKYKVVKIEPLGVAPTKAKRRVVQREKRAAG.IGALFLGFLGAAGSTMGAASMTLTVQARQLLSGIVQQQNNLLRAIEAQ**

**H18814.10.1C5_CrNA**  **YKYKVVKIEPLGVAPTKAKRRVVQREKRAAG.IGALFLGFLGAAGSTMGAASMTLTVQARQLLSGIVQQQNNLLRAIEAQ**

**H18814.10.1B1_CrNA**  **YKYKVVKIEPLGVAPTKAKRRVVQREKRAAG.IGALFLGFLGAAGSTMGAASMTLTVQARQLLSGIVQQQNNLLRAIEAQ**

**H18814.10.1G2_CrNA**  **YKYKVVKIEPLGVAPTKAKRRVVQREKRAAG.IGALFLGFLGAAGSTMGAASMTLTVQARQLLSGIVQQQNNLLRAIEAQ**

**H11668.12.F11(A)_CrNA**  **YKYKVVKIEPLGVAPTKAKRRVVQREKRAVG.IGAVFLGFLGAAGSTMGAASLTLTVQARQLLSGIVQQQNNLLRAIEAQ**

**H11668.12.C3_CrNA**  **YKYKVVKIEPLGVAPTKAKRRVVQREKRAIG.IGAVFLGFLGAAGSTMGAASLTLTVQARQLLSGIVQQQNNLLRAIEAQ**

**H11668.12.D11_CrNA**  **YKYKVVKIEPLGVAPTKAKRRVVQREKRAIG.IGAVFLGFLGAAGSTMGAASLTLTVQARQLLSGIVQQQNNLLRAIEAQ**

**H11668.12.H9_CrNA**  **YKYKVVKIEPLGVAPTKAKRRVVQREKRAIG.IGAVFLGFLGAAGSTMGAASLTLTVQARQLLSGIVQQQNNLLRAIEAQ**

**H11668.12.E10_CrNA**  **YKYKVVKVEPLGVAPTKAKRRVVQREKRAIG.IGAVFLGFLGAAGSTMGAASLTLTVQARQLLSGIVQQQNNLLRAIEAQ**

**H19308.26.B1_CrNA**  **YKYKVVKIEPLGVAPTKAKRRVVQREKRAVGTLGAVFLGFLGAAGSTMGAASMTLTVQARLLLSGIVQQQNNLLKAIEAQ**

**H19308.26.F8I_CrNA**  **YKYKVVKIEPLGVAPTKAKRRVVQREKRAVGTLGAVFLGFLGAAGSTMGAASMTLTVQARLLLSGIVQQQNNLLRAIEAQ**

**H19308.26.D8_CrNA**  **YKYKVVKIEPLGVAPTKAKRRVVQREKRAVGTLGAMFLGFLGAAGSTMGAASMTLTVQARLLLSGIVQQQNNLLRAIEAQ**

**H19308.26.D1_CrNA**  **YKYKVVKIEPLGVAPTKAKRRVVQREKRAVGTLGAMFLGFLGAAGSTMGAASMTLTVQARLLLSGIVQQQNNLLRAIEAQ**

**H19308.26.E4_CrNA**  **YKYKVVKIEPLGVAPTRAKRRVVQREKRAVGTLGAVFLGFLGAAGSTMGAASMTLTVQARLLLSGIVQQQNNLLRAIEAQ**

**H19885.31.D9_CrNA**  **YKYKVVKIQPLGVAPTKAKRRVVQREKRAV.TLGAMFLGFLGAAGSTMGAASVTLTVQARQLLSGIVQQQNNLLKAIEAQ**

**H19885.31.G12_CrNA**  **YKYKVVKIQPLGVAPTKAKRRVVQREKRAV.TLGAMFLGFLGAAGSTMGAASVTLTVQARQLLSGIVQQQNNLLKAIEAQ**

**H19885.31.E2_CrNA**  **YKYKVVKIQPLGVAPTKAKRRVVQREKRAV.TLGAMFLGFLGAAGSTMGAASVTLTVQARQLLSGIVQQQNNLLKAIEAQ**

**H19885.31.G10_CrNA**  **YKYKVVKIQPLGVAPTKAKRRVVQREKRAV.TLGAMFLGFLGAAGSTMGAASVTLTVQARQLLSGIVQQQNNLLKAIEAQ**

**H19885.31.H11_CrNA**  **YKYKVVKIQPLGVAPTKAKRRVVQREKRAV.TLGAMFLGFLGAAGSTMGAASVTLTVQARQLLSGIVQQQNNLLKAIEAQ**

**H19885.31.A5_CrNA**  **YKYKVVKIQPLGVAPTKAKRRVVQREKRAV.TLGAMFLGFLGAAGSTMGAASVTLTVQARQLLSGIVQQQNNLLKAIEAQ**

**H19885.31.F8_CrNA**  **YKYKVVKIQPLGVAPTKAKRRVVQREKRAV.TLGAMFLGFLGAAGSTMGAASVTLTVQARQLLSGIVQQQNNLLKAIEAQ**

**H19885.31.H10_CrNA**  **YKYKVVKIQPLGVAPTKAKRRVVQREKRAV.TLGAMFLGFLGAAGSTMGAASVTLTVQARQLLSGIVQQQNNLLKAIEAQ**

**H19885.31.G1_CrNA**  **YKYKVVKIQPLGVAPTKAKRRVVQREKRAV.TLGAMFLGFLGAAGSTMGAASVTLTVQARQLLSGIVQQQNNLLKAIEAQ**

**H19885.31.C6_CrNA**  **YKYKVVKIQPLGVAPTKAKRRVVQREKRAV.TLGAMFLGFLGAAGSTMGAASVTLTVQARQLLSGIVQQQNNLLKAIEAQ**

**H19885.31.E11_CrNA**  **YKYKVVKIQPLGVAPTKAKRRVVQREKRAV.TLGAMFLGFLGAAGSTMGAASVTLTVQARQLLSGIVQQQNNLLKAIEAQ**

**H18969.12.9D9_CrNA**  **YKYKVVKIEPLGVAPTKAKRRVVQREKRAVGTIGAMFLGFLGAAGSTMGAASMTLTVQARQLLSGIVQQQNNLLRAIKAQ**

**H18969.12.8E6_CrNA**  **YKYKVVKIEPLGVAPTKAKRRVVQREKRAVGTIGAMFLGFLGAAGSTMGAASMTLTVQARQLLSGIVQQQNNLLRTIKAQ**

**H18969.12.8G8_CrNA**  **YKYKVVKIEPLGVAPTKAKRRVVQREKRAVGTIGAMFLGFLGAAGSTMGAASMMLTVQARQLLSGIVQQQNNLLRAIEAQ**

**H18969.12.6D7_CrNA**  **YKYKVVKIEPLGVAPTKAKRRVVQREKRAVGTIGAVFLGFLGAAESTMGAASMTLTVQARQLLSGIVQQQNNLLKAIEAQ**

**H18969.12.8B4_CrNA**  **YKYKVVKIEPLGVAPTKAKRRVVQREKRAVGTIGAMFLGFLGAAGSTMGAASMTLTVQARQLLSGIVQQQNNLLRAIEAQ**

**H18969.12.7D5_CrNA**  **YKYKVVKIEPLGVAPTKAKRRVVQREKRAVGTIGAMFLGFLGAAGSTMGAASMTLTVQARQLLSGIVQQQNNLLRAIEAQ**

**H18969.12.6C4_CrNA**  **YKYKVVKIEPLGVAPTKAKRRVVQREKRAVGTIGAMFLGFLGAAGSTMGAASMTLTVQARQLLSGIVQQQNNLLRAIEAQ**

**H18969.12.10H3_CrNA**  **YKYKVVKIEPLGVAPTKAKRRVVQREKRAVGTIGAMFLGFLGAAGSTMGAASMTLTVQARQLLSGIVQQQNNLLRAIEAQ**

**H19329.32.C9_Non-CrNA**  **YKYKVVKIEPLGVAPTKAKRRVVQREKRAI.TLGAMFLGFLGAAGSTMGAASMALTVQARQLLSGIVQQQNNLLRAIEAQ**

**H19329.32.E6_Non-CrNA**  **YKYKVVKIEPLGVAPTKAKRRVVQREKRAI.TLGAMFLGFLGAAGSTMGAASMALTVQARQLLSGIVQQQNNLLRAIEAQ**

**H19329.32.H7_Non-CrNA**  **YKYKVVKIEPLGVAPTKAKRRVVQREKRAI.TLGAMFLGFLGAAGSTMGAASMALTVQARQLLSGIVQQQNNLLRAIEAQ**

**H19329.32.F1_Non-CrNA**  **YKYKVVKIEPLGVAPTKAKRRVVQREKRAI.TLGAMFLGFLGAAGSTMGAASMALTVQARQLLSGIVQQQNNLLRAIEAQ**

**H19329.32.H5_Non-CrNA**  **YKYKVVKIEPLGVAPTKAKRRVVQREKRAI.TLGAMFLGFLGAAGSTMGAASMALTVQARQLLSGIVQQQNNLLRAIEAQ**

**H19329.32.H9_Non-CrNA**  **YKYKVVKIEPLGVAPTKAKRRVVQREKRAI.TLGAMFLGFLGAAGSTMGAASMALTVQARQLLSGIVQQQNNLLRAIEAQ**

**H19329.13.F12_Non-CrNA**  **YKYKVVKIEPLGVAPTKAKRRVVQREKRAI.TLGAMFLGFLGAAGSTMGAASMALTVQARQLLSGIVQQQNNLLRAIEAQ**

**H18887.21.G2_Non-CrNA**  **YKYKVVQIEPLGIAPTKAKRRVVQREKRAVG.IGAMFLGVLSAAGSTMGAASMTLTVQARQLLSGIVQQQNNLLRAIEAQ**

**H18887.21.roD7_Non-CrNA** **YKYKVVQIEPLGIAPTKAKRRVVQREKRAVG.IGAMFLGVLSAAGSTMGAASMTLTVQARQLLSGIVQQQNNLLRAIEAQ**

**H19861.19.C10_Non-CrNA**  **YKYKVVKIEPLGVAPTKAKRRVVQREKRAVGTLGAMFLGFLGAAGSTMGAASMTLTVQARQLLSGIVQQQNNLLKAIDAQ**

**H19861.19.F2_Non-CrNA**  **YKYKVVKIEPLGVAPTKAKRRVVQREKRAVGTLGAMFLGFLGAAGSTMGAASMTLTVQARQLLSGIVQQQNNLLKAIDAQ**

**H19861.19.A6_Non-CrNA**  **YKYKVVKIEPLGVAPTKAKRRVVQREKRAVGTLGAMFLGFLGAAGSTMGAASMTLTVQARQLLSGIVQQQNNLLKAIDAQ**

**H19489.8.G5_Non-CrNA**  **YKYKVVKIEPLGVAPTKAKRRVVQREKRAVGTIGAMFLGFLGAAGSTMGAASVTLTVQARLLLSGIVQQQNNLLKAIEAQ**

**H19489.8.1E10_Non-CrNA**  **YKYKVVKIEPLGVAPTKAKRRVVQREKRAVGTIGAMFLGFLGAAGSTMGAASVTLTVQARLLLSGIVQQQNNLLRAIEAQ**

**H19489.8.1A11_Non-CrNA**  **YKYKVVKIEPLGVAPTKAKRRVVQREKRAVGTIGAMFLGFLGAAGSTMGAASVTLTVQARLLLSGIVQQQNNLLRAIEAQ**

**H19489.8.1H10_Non-CrNA**  **YKYKVVKIEPLGVAPTKAKRRVVQREKRAVGTIGAMFLGFLGAAGSTMGAASVTLTVQARLLLSGIVQQQNNLLRAIEAQ**

**H19489.8.2A3_Non-CrNA**  **YKYKVVKIEPLGVAPTKAKRRVVQREKRAVGTIGAMFLGFLGAAGSTMGAASVTLTVQARLLLSGIVQQQNNLLRAIEAQ**

**H19974.11.E12_Non-CrNA**  **YKYKVVKIEPLGVAPTKAKRRVVQREKRAVGTIGAMFLGFLGAAGSTMGAASMTLTVQARLLLSGIVQQQSNLLRAIEAQ**

**H19974.11.E11_Non-CrNA**  **YKYKVVKIEPLGVAPTKAKRRVVQREKRAVGTIGAMFLGFLGAAGSTMGAASMTLTVQARLLLSGIVQQQSNLLRAIEAQ**

**H19792.9.F6_Non-CrNA**  **YKYKVVKIEPLGVAPTKAKRRVVQREKRAVGVIGAMFLGFLGAAGSTMGAASMTLTVQARQLLSGIVQQQNNLLRAIEAQ**

**H19792.9.B1_Non-CrNA**  **YKYKVVKIEPLGVAPTKAKRRVVQREKRAVGVIGAMFLGFLGAAGSTMGAASMTLTVQARQLLSGIVQQQNNLLRAIEAQ**

**H19792.9.F1_Non-CrNA**  **YKYKVVKIEPLGVAPTKAKRRVVQREKRAVGVIGAMFLGFLGAAGSTMGAASMTLTVQARQLLSGIVQQQNNLLRAIEAQ**

**H19792.9.D6_Non-CrNA**  **YKYKVVKIEPLGVAPTKAKRRVVQREKRAVGVIGAMFLGFLGAAGSTMGAASMTLTVQARQLLSGIVQQQNNLLRAIEAQ**

**H19792.9.C10_Non-CrNA**  **YKYKVVKIEPLGVAPTKAKRRVVQREKRAVGVIGAMFLGFLGAAGSTMGAASMTLTVQARQLLSGIVQQQNNLLRAIEAQ**

**H18880.10.20_Non-CrNA**  **YKYKVVKIEPLGVAPTKAKRRVVQREKRAVGTLGAMFLGFLGAAGSTMGAASTALTVQARQLLSGIVQQQNNLLRAIEAQ**

**H18880.10.21_Non-CrNA**  **YKYKVVKIEPLGVAPTKAKRRVVQREKRAVGTLGAMFLGFLGAAGSTMGAASTALTVQARQLLSGIVQQQNNLLRAIEAQ**

**H19961.14.F10_Non-CrNA**  **YKYKVVKIEPLGIAPTKAKRRVVQREKRAVG.IGAVFLGFLGAAGSTMGAASTALTVQARLLLSGIVQQQNNLLRAIKAQ**

**H19961.14.E8_Non-CrNA**  **YKYKVVKIEPLGIAPTKAKRRVVQREKRAVG.IGAVFLGFLGAAGSTMGAVSTALTVQARLLLSGIVQQQNNLLRAIEAQ**

**H19961.14.G4_Non-CrNA**  **YKYKVVKIEPLGIAPTKAKRRVVQREKRAVG.IGAVFLGFLGAAGSTMGAASTALTVQARLLLSGIVQQQNNLLRAIEAQ**

**H19961.14.B10_Non-CrNA**  **YKYKVVKIEPLGIAPTKAKRRVVQREKRAVG.IGAVFLGFLGAAGSTMGAASTALTVQARLLWSGIVQQQNNLLRAIEAQ**

**H19961.14.F9_Non-CrNA**  **YKYKVVKIEPLGIAPTKAKRRVVQRDKRAVG.IGAVFLGFLGAAGSTMGAASTALTVQARLLLSGIVQQQNNLLRAIEAQ**

**H19576.9.H1_Non-CrNA**  **YKYKVVKIEPLGVAPTKAKRRVVQREKRAVG.LGAMFLGFLGAAGSTMGAASITLTVQARLLLSGIVQQQNNLLRAIEAQ**

**H19576.9.F4_Non-CrNA**  **YKYKVIKIEPLGVAPTKAKRRVVQREKRALG.IGAMFLGFLGAAGSTMGAASITLTVQARLLLSGIVQQQNNLLRAIEAQ**

650 660 670 680 690 700 710 720

....|....|....|....|....|....|....|....|....|....|....|....|....|....|....|....|

**HXB2/1-856**  **QHLLQLTVWGIKQLQARILAVERYLKDQQLLGIWGCSGKLICTTAVPWNASWSNKSLEQIWNHTTWMEWDREINNYTSLI**

**H18818.6.1D2_CrNA**  **QHLLQLTVWGIKQLQARVLAVERYLRDQQLLGIWGCSGKLICTTSVPWNTSWSNKSLDKIWNNMTWMEWEREIDNYTSLI**

**H18818.6.1C3_CrNA**  **QHLLQLTVWGIKQLQARVLAVERYLRDQQLLGIWGCSGKLICTTSVPWNTSWSNKSLDKIWNNMTWMEWEREIDNYTSLI**

**H18818.6.1A6_CrNA**  **QHLLQLTVWGIKQLQARVLAVERYLRDQQLLGIWGCSGKLICTTSVPWNTSWSNKSLDKIWNNMTWMEWEREIDNYTSLI**

**H18818.6.1G12_CrNA**  **QHLLQLTVWGIKQLQARVLAVERYLRDQQLLGIWGCSGKLICTTSVPWNTSWSNKSLDKIWNNMTWMEWEREIDNYTSLI**

**H19829.11.H5_CrNA**  **QHLLQLTVWGIKQLQARVLAVERYLRDQQLLGIWGCSGKLICTTSVPWNTSWSNKSLDKIWDNMTWMEWEREIDNYTSLI**

**H19829.11.A2_CrNA**  **QHLLQLTVWGIKQLQARVLAVERYLRDQQLLGIWGCSGKLICTTSVPWNTSWSNKSLNKIWNNMTWMEWEREIDNYTSLI**

**H19829.11.E8_CrNA**  **QHLLQLTVWGIKQLQARVLAVERYLRDQQLLGIWGCSGKLICTTSVPWNTSWSNKSLEKIWNNMTWMEWEREIDNYTSLI**

**H19829.11.B4_CrNA**  **QHLLQLTVWGIKQLQARVLAVERYLRDQQLLGIWGCSGKLICTTSVPWNTSWSNKSLEKIWDNMTWMEWEREIDNYTSLI**

**H19829.11.A4_CrNA**  **QHLLQLTVWGIKQLQARVLAVERYLRDQQLLGIWGCSGKLICTTSVPWNTSWSNKSLDKIWDNMTWMEWEREIDNYTSLI**

**H19829.11.G8_CrNA**  **QHLLQLTVWGIKQLQARVLAVERYLRDQQLLGIWGCSGKLICTTSVPWNTSWSNKSLEKIWNNMTWMEWEREIDNYTSLI**

**H19999.7.1G10_CrNA**  **QHLLQLTVWGIKQLQARVLAVERYLKDQQLLGIWGCSGKLICTTAVPWNASWSNKSLDKIWNNMTWMEWEREINNYTSLI**

**H19999.7.2G7_CrNA**  **QHLLQLTVWGIKQLQARVLAVERYLKDQQLLGIWGCSGKLICTTAVPWNASWSNKSLDKIWNNMTWMEWEREINNYTSLI**

**H19999.7.2D5_CrNA**  **QHLLQLTVWGIKQLQARVLAVERYLKDQQLLGIWGCSGKLICTTAVPWNASWSNKSLDKIWNNMTWMEWEREINNYTSLI**

**H19999.7.1B2_CrNA**  **QHLLQLTVWGIKQLQARVLAVERYLKDQQLLGIWGCSGKLICTTAVPWNASWSNKSLDTIWNNMTWMEWEREINNYTREI**

**H19999.7.1D2_CrNA**  **QHLLQLTVWGIKQLQARVLAVERYLKDQQLLGIWGCSGKLICTTAVPWNASWSNKSLDSIWNNMTWMEWEREINNYTREI**

**H19507.18.G11_CrNA**  **QHLLQLTVWGIKQLQARVLAVERYLKDQQLLGIWGCSGKLICTTAVPWNASWSNKSLDQIWNNMTWMEWEREIDNYTNLI**

**H19507.18.C11_CrNA**  **QHLLQLTVWGIKQLQARVLAVERYLKDQQLLGIWGCSGKLICTTAVPWNASWSNKSLDQIWNNMTWMEWEREIDNYTNLI**

**H19507.18.A11_CrNA**  **QHLLQLTVWGIKQLQARVLAVERYLKDQQLLGIWGCSGKLICTTAVPWNASWSNKSLDQIWNNMTWMEWEREIDNYTNLI**

**H19507.18.F4_CrNA**  **QHLLQLTVWGIKQLQARVLAVERYLKDQQLLGIWGCSGKLICTTAVPWNASWSNKSLDQIWNNMTWMEWEREIDNYTNLI**

**H19793.13.F8_CrNA**  **QHLLQLTVWGIKQLQARVLAVEKYLKDQQLLGIWGCSGKLICTTTVPWNASWSNKSLDKIWNNMTWMEWEREIDNYTSLI**

**H19463.8.A11_CrNA**  **QHLLQLTVWGIKQLQARVLAVERYLKDQQLLGIWGCSGKLICTTAVPWNASWSNKSLDQIWNNMTWMEWEREINNYTGLI**

**H19463.8.E10_CrNA**  **QHLLQLTVWGIKQLQARVLAVERYLKDQQLLGIWGCSGKLICTTAVPWNASWSNKSLDQIWNNMTWMEWEREINNYTGLI**

**H19474.17.1G12_CrNA**  **QHMLQLTVWGIKQLQARVLAVERYLKDQQLLGIWGCSGKLICTTTVPWNASWSNKSLDKIWNNMTWMEWEREIDNYTSLI**

**H19474.17.2H8_CrNA**  **QHMLQLTVWGIKQLQARVLAVERYLKDQQLLGIWGCSGKLICTTTVPWNASWSNKSLDKIWNNMTWMEWEREIDNYTSLI**

**H18814.10.1E1_CrNA**  **QHLLQLTVWGIKQLQARVLAVERYLKDQQLLGIWGCSGKLICTTAVPWNDSWSNKSLTEIWDNMTWMQWEKEINNYTNRI**

**H18814.10.1B4_CrNA**  **QHLLQLTVWGIKQLQARVLAVERYLKDQQLLGIWGCSGKLICTTAVPWNDSWSNKSLNDIWNNMTWMQWEKEINNYTDKI**

**H18814.10.1C5_CrNA**  **QHLLQLTVWGIKQLQARVLAVERYLKDQQLLGIWGCSGKLICTTAVPWNDSWSNKSLNDIWNNMTWMQWEKEINNHTDKI**

**H18814.10.1B1_CrNA**  **QHLLQLTVWGIKQLQARVLAVERYLKDQQLLGIWGCSGKLICTTAVPWNDSWSNKSLNDIWNNMTWMQWEKEINNYTDKI**

**H18814.10.1G2_CrNA**  **QHLLQLTVWGIKQLQARVLAVERYLKDQQLLGIWGCSGKLICTTAVPWNDSWSNKSLNDIWNNMTWMQWEKEINNHTDKI**

**H11668.12.F11(A)_CrNA**  **QHLLQLTVWGIKQLQARVLAVERYLQDQQLLGIWGCSGKLICTTAVPWNASWSNKSLDDIWNNMTWMEWEREISNYTNKI**

**H11668.12.C3_CrNA**  **QHLLQLTVWGIKQLQARVLAVERYLQDQQLLGIWGCSGKLICTTAVPWNASWSNKSLDDIWNNMTWMEWEREISNYTNKI**

**H11668.12.D11_CrNA**  **QHLLQLTVWGIKQLQARVLAVERYLQDQQLLGIWGCSGKLICTTAVPWNASWSNKSLDDIWNNMTWMEWEREISNYTNKI**

**H11668.12.H9_CrNA**  **QHLLQLTVWGIKQLQARVLAVERYLQDQQLLGIWGCSGKLICTTAVPWNASWSNKSLDDIWNNMTWMEWEREISNYTHKI**

**H11668.12.E10_CrNA**  **QHLLQLTVWGIKQLQARVLAVERFLQDQQLLGIWGCSGKLICTTAVPWNASWSNKSLDDIWNNMTWMEWEREISNYTNKI**

**H19308.26.B1_CrNA**  **QHLLQLTVWGIKQLQARVLAVERYLKDQQLLGIWGCSGKLICTTAVPWNASWSNKSLNAIWDNMTWMEWEREIDNYTSLI**

**H19308.26.F8I_CrNA**  **QHLLQLTVWGIKQLQARVLAVERYLKDQQLLGIWGCSGKLICTTAVPWNASWSNKSLNAIWDNMTWMEWEREIDNYTSLI**

**H19308.26.D8_CrNA**  **QHLLQLTVWGIKQLQARVLAVERYLKDQQLLGIWGCSGKLICTTAVPWNASWSNKSLNAIWDNMTWMEWEREIDNYTSLI**

**H19308.26.D1_CrNA**  **QHLLQLTVWGIKQLQARVLAVERYLKDQQLLGIWGCSGKLICTTAVPWNASWSNKSLNAIWDNMTWMEWEREIDNYTSLI**

**H19308.26.E4_CrNA**  **QHLLQLTVWGIKQLQARVLAVERYLQDQQLLGIWGCSGKLICTTAVPWNASWSNKSLNAIWDNMTWMEWEREIDNYTSLI**

**H19885.31.D9_CrNA**  **QRILQLTVWGIKQLQARVLAVERYLQDQQLLGIWGCSGKLICTTTVPWNASWSNKSLDRIWNNMTWMEWEKEIDNYTDLI**

**H19885.31.G12_CrNA**  **QRILQLTVWGIKQLQARVLAVERYLQDQQLLGIWGCSGKLICTTTVPWNASWSNKSLDRIWNNMTWMEWEKEIDNYTDLI**

**H19885.31.E2_CrNA**  **QRILQLTVWGIKQLQARVLAVERYLQDQQLLGIWGCSGKLICTTTVPWNASWSNKSLDRIWNNMTWMEWEKEIDNYTDLI**

**H19885.31.G10_CrNA**  **QRILQLTVWGIKQLQARVLAVERYLQDQQLLGIWGCSGKLICTTTVPWNASWSNKSLDRIWNNMTWMEWEKEIDNYTDLI**

**H19885.31.H11_CrNA**  **QRILQLTVWGIKQLQARVLAVERYLQDQQLLGIWGCSGKLICTTTVPWNASWSNKSLDRIWNNMTWMEWEKEIDNYTDLI**

**H19885.31.A5_CrNA**  **QRILQLTVWGIKQLQARVLAVERYLQDQQLLGIWGCSGKLICTTTVPWNASWSNKSLDRIWNNMTWMEWEKEIDNYTDLI**

**H19885.31.F8_CrNA**  **QRILQLTVWGIKQLQARVLAVERYLQDQQLLGIWGCSGKLICTTTVPWNASWSNKSLDRIWNNMTWMEWEKEIDNYTDLI**

**H19885.31.H10_CrNA**  **QRILQLTVWGIKQLQARVLAVERYLQDQQLLGIWGCSGKLICTTTVPWNASWSNKSLDRIWNNMTWMEWEKEIDNYTDLI**

**H19885.31.G1_CrNA**  **QRILQLSVWGIKQLQARVLAVERYLQDQQLLGIWGCSGKLICTTTVPWNASWSNKSLDRIWNNMTWMEWEKEIDNYTDLI**

**H19885.31.C6_CrNA**  **QRILQLTVWGIKQLQARVLAVERYLQDQQLLGIWGCSGKLICTTTVPWNASWSNKSLDRIWNNMTWMEWEKEIDNYTDLI**

**H19885.31.E11_CrNA**  **QRILQLTVWGIKQLQARVLAVERYLQDQQLLGIWGCSGKLICTTTVPWNASWSNKSLDRIWNNMTWMEWEKEIDNYTDLI**

**H18969.12.9D9_CrNA**  **QHLLQLTVWGIKQLQARVLAVERYLKDQQLLGIWGCSGKLICTTAVPWNASWSNKSLDKIWNNMTWMDWEREINNYTGLI**

**H18969.12.8E6_CrNA**  **QHLLQLTVWGIKQLQARVLAVERYLKDQQLLGIWGCSGKLICTTAVPWNASWSNKSLDKIWNNMTWMDWEREINNYTGLI**

**H18969.12.8G8_CrNA**  **QHLLQLTVWGIKQLQARVLAVERYLKDQQLLGIWGCSGKLICTTAVPWNASWSNKSLDKIWNNMTWMDWEREINNYTGLI**

**H18969.12.6D7_CrNA**  **QHLLQLTVWGIKQLQARVLAVERYLKDQQLLGIWGCSGKLICTTAVPWNASWSNKSLDKIWNNMTWMDWEREINNYTGLI**

**H18969.12.8B4_CrNA**  **QHLLQLTVWGIKQLQARVLAVERYLKDQQLLGIWGCSGKLICTTAVPWNASWSNKSLDKIWNNMTWMDWEREINNYTGLI**

**H18969.12.7D5_CrNA**  **QHLLQLTVWGIKQLQARVLAVERYLKDQQLLGIWGCSGKLICTTAVPWNASWSNKSLDKIWNNMTWMDWEREINNYTGLI**

**H18969.12.6C4_CrNA**  **QHLLQLTVWGIKQLQARVLAVERYLMDQQLLGIWGCSGKLICTTAVPWNASWSNKSLDKIWNNMTWMDWEREINNYTGLI**

**H18969.12.10H3_CrNA**  **QHLLQLTVWGIKQLQARVLAVERYLKDQQLLGIWGCSGKLICTTAVPWNASWSNKSLDKIWNNMTWMDWEREINNYTGLI**

**H19329.32.C9_Non-CrNA**  **QHLLQLTVWGIKQLQARVLAIERYLQDQQLLGIWGCSGKLICTTTVPWNASWSNKSLNQIWNNMTWMQWEREIDNYTSLI**

**H19329.32.E6_Non-CrNA**  **QHLLQLTVWGIKQLQARVLAIERYLQDQQLLGIWGCSGKLICTTTVPWNASWSNKSLNQIWNNMTWMQWEREIDNYTSLI**

**H19329.32.H7_Non-CrNA**  **QHLLQLTVWGIKQLQARVLAIERYLQDQQLLGIWGCSGKLICTTTVPWNASWSNKSLNQIWNNMTWMQWEREIDNYTSLI**

**H19329.32.F1_Non-CrNA**  **QHLLQLTVWGIKQLQARVLAIERYLQDQQLLGIWGCSGKLICTTTVPWNASWSNKSLNQIWNNMTWMQWEREIDNYTSLI**

**H19329.32.H5_Non-CrNA**  **QHLLQLTVWGIKQLQARVLAIERYLQDQQLLGIWGCSGKLICTTTVPWNASWSNKSLNQIWNNMTWMQWEREIDNYTSLI**

**H19329.32.H9_Non-CrNA**  **QHLLQLTVWGIKQLQARVLAIERYLQDQQLLGIWGCSGKLICTTTVPWNASWSNKSLNQIWNNMTWMQWEREIDNYTSLI**

**H19329.13.F12_Non-CrNA**  **QHLLQLTVWGIKQLQARVLAIERYLQDQQLLGIWGCSGKLICTTTVPWNASWSNKSLNQIWNNMTWMQWEREIDNYTSLI**

**H18887.21.G2_Non-CrNA**  **QHLLQLTVWGIKQLQARVLAVERYLKDQQLLGIWGCSGKLICTTTVPWNISWSNKTLDYIWNNMTWMQWEKEIDNYTDLI**

**H18887.21.roD7_Non-CrNA** **QHLLQLTVWGIKQLQARVLAVERYLKDQQLLGIWGCSGKLICTTTVPWNISWSNRTLDYIWNNMTWMQWEKEIDNYTGLI**

**H19861.19.C10_Non-CrNA**  **QHLLQLTVWGIKQLQARVLAVERYLKDQQLLGIWGCSGKLICTTTVPWNVSWSNKSLNQIWENMTWMQWEKEIDNYTSLI**

**H19861.19.F2_Non-CrNA**  **QHLLQLTVWGIKQLQARVLAVERYLKDQQLLGIWGCSGKLICTTTVPWNVSWSNKSLNQIWENMTWMQWEKEIDNYTSLI**

**H19861.19.A6_Non-CrNA**  **QHLLQLTVWGIKQLQARVLAVERYLKDQQLLGIWGCSGKLICTTTVPWNVSWSNKSLNQIWENMTWMQWEKEIDNYTSLI**

**H19489.8.G5_Non-CrNA**  **QHMLQLTVWGIKQLQARVLAVERYLRDQQLLGIWGCSGKLICTTAVPWNNSWSNRSLDMIWNNMTWMEWEREIDNYTGLI**

**H19489.8.1E10_Non-CrNA**  **QHMLQLTVWGIKQLQARVLAVERYLRDQQLLGIWGCSGKLICTTAVPWNNSWSNRSLDMIWNNMTWMEWEREIDNYTGLI**

**H19489.8.1A11_Non-CrNA**  **QHMLQLTVWGIKQLQARVLAVERYLRDQQLLGIWGCSGKIICTTAVPWNNSWSNRSLDMIWNNMTWMEWEREIDNYTGLI**

**H19489.8.1H10_Non-CrNA**  **QHMLQLTVWGIKQLQARVLAVERYLRDQQLLGIWGCSGKLICTTAVPWNNSWSNRSLDMIWNNMTWMEWEREIDNYTGLI**

**H19489.8.2A3_Non-CrNA**  **QHMLQLTVWGIKQLQARVLAVERYLRDQQLLGIWGCSGKLICTTAVPWNNSWSNRSLDMIWNNMTWMEWEREIDNYTGLI**

**H19974.11.E12_Non-CrNA**  **QHLLQLTVWGIKQLQARVLAVERYLKDQQLLGIWGCSGKLICTTAVPWNASWSNKSLDNIWNNMTWMEWEREISNYTNLI**

**H19974.11.E11_Non-CrNA**  **QHLLQLTVWGIKQLQARVLAVERYLKDQQLLGIWGCSGKLICTTAVPWNASWSNKSLDNIWNNMTWMEWEKEISNYTNLI**

**H19792.9.F6_Non-CrNA**  **QHLLQLTVWGIKQLQARVLAVERYLRDQQLLGIWGCSGKIICTTTVPWNASWSNKSLDNIWQNMTWMEWEREISNYTSLI**

**H19792.9.B1_Non-CrNA**  **QHLLQLTVWGIKQLQARVLAVERYLRDQQLLGIWGCSGKLICTTTVPWNASWSNKSLDNIWQNMTWMEWEREISNYTSLI**

**H19792.9.F1_Non-CrNA**  **QHLLQLTVWGIKQLQARVLAVERYLRDQQLLGIWGCSGKLICTTTVPWNASWSNKSLDNIWQNMTWMEWEREISNYTSLI**

**H19792.9.D6_Non-CrNA**  **QHLLQLTVWGIKQLQARVLAVERYLRDQQLLGIWGCSGKLICTTTVPWNASWSNKSLDNIWQNMTWMEWEREISNYTSLI**

**H19792.9.C10_Non-CrNA**  **QHLLQLTVWGIKQLQARVLAVERYLRDQQLLGIWGCSGKLICTTTVPWNASWSNKSLDNIWQNMTWMEWEREISNYTSLI**

**H18880.10.20_Non-CrNA**  **QHLLQLTVWGIKQLQARVLAVERYLRDQQLLGIWGCSGKLICTTTVPWNASWSNKSLDKIWNNMTWMQWEREIDNYTSLI**

**H18880.10.21_Non-CrNA**  **QHLLQLTVWGIKQLQARVLAVERYLRDQQLLGIWGCSGKLICTTTVPWNASWSNKSLDKIWNNMTWMEWEREIDNYTSLI**

**H19961.14.F10_Non-CrNA**  **QHMLQLTVWGIKQLQARILAVERYLKDQQLLGIWGCSGKLICTTAVPWNASWSNKSVDEIWGNMTWMQWEREIDNYTSLI**

**H19961.14.E8_Non-CrNA**  **QHMLQLTVWGIKQLQARVLAVERYLKDQQLLGIWGCSGKLICTTAVPWNASWSNKSVDEIWGNMTWMQWEREIDNYTSLI**

**H19961.14.G4_Non-CrNA**  **QHMLQLTVWGIKQLQARVLAVERYLKDQQLLGIWGCSGKLICTTAVPWNASWSNKSVDEIWGNMTWMQWEREIDNYTSLI**

**H19961.14.B10_Non-CrNA**  **QHMLQLTVWGIKQLQARVLAVERYLKDQQLLGIWGCSGKLICTTAVPWNASWSNKSVDEIWGNMTWMQWEREIDNYTSLI**

**H19961.14.F9_Non-CrNA**  **QHMLQLTVWGIKQLQARVLAVERYLKDQQLLGIWGCSGKLICTTAVPWNASWSNKSVDEIWGNMTWMQWEREIDNYTSLI**

**H19576.9.H1_Non-CrNA**  **QHLLQLTVWGIKQLQARVLAVERYLKDQQLLGIWGCSGKLICTTTVPWNASWSNKSLDKIWNNMTWMEWEREIDNYTSLI**

**H19576.9.F4_Non-CrNA**  **QHLLQLTVWGIKQLQARVLAVERYLKDQQLLGIWGCSGKLICTTTVPWNASWSNKSLDKIWNNMTWMEWEREIDNYTSLI**

730 740 750 760 770 780 790 800

....|....|....|....|....|....|....|....|....|....|....|....|....|....|....|....|

**HXB2/1-856**  **HSLIEESQNQQEKNEQELLELDKWASLWNWFNITNWLWYIKLFIMIVGGLVGLRIVFAVLSIVNRVRQGYSPLSFQTHLP**

**H18818.6.1D2_CrNA**  **YTLLEESQNQQEKNEQELLELDKWASLWNWFDITKWLWYIKIFIMIVGGLVGLRIIFTVLSIVNRVRQGYSPLSFQTHLP**

**H18818.6.1C3_CrNA**  **YTLLEESQNQQEKNEQELLELDKWASLWNWFDITSWLWYIKIFIMIVGGLVGLRIVFTVLSIVNRVRQGYSPLSFQTHLP**

**H18818.6.1A6_CrNA**  **YTLLEESQNQQEKNEQELLELDKWASLWNWFDITNWLWYIKIFIMIVGGLVGLRIVFTVLSIVNRVRQGYSPLSFQTHLP**

**H18818.6.1G12_CrNA**  **YTLLEESQNQQEKNEQELLELDKWASLWNWFDITSWLWYIKIFIMIVGGLVGLRIVFTVLSIVNRVRQGYSPLSFQTHLP**

**H19829.11.H5_CrNA**  **YTLLEESQNQQEKNEQELLELDKWASLWNWFDITNWLWYIKMFIMIVGGLVGLRIVFTVLSIVNRVRQGYSPLSFQTHLP**

**H19829.11.A2_CrNA**  **YTLLEESQNQQEKNEQELLELDKWASLWNWFDITNWLWYIKIFIMIVGGLVGLRIVFTVLSIVNRVRQGYSPLSFQTHLP**

**H19829.11.E8_CrNA**  **YTLLEESQNQQEKNEQELLELDKWASLWNWFDITNWLWYIKIFIMIVGGLVGLRIVFTVLSIVNRVRQGYSPLSFQTHLP**

**H19829.11.B4_CrNA**  **YTLLEESQNQQEKNEQELLELDKWASLWNWFDITNWLWYIKIFIMIVGGLVGLRIVFTILSIVNRVRQGYSPLSFQTHLP**

**H19829.11.A4_CrNA**  **YTLLEESQNQQEKNEQELLELDKWASLWNWFDITNWLWYIKIFIMIVGGLVGLRIVFTVLSIVNRVRQGYSPLSFQTHLP**

**H19829.11.G8_CrNA**  **YTLLEESQNQQEKNEQELLELDKWASLWNWFDITNWLWYIKIFIMIVGGLVGLRIVFTVLSIVNRVRQGYSPLSFQTHLP**

**H19999.7.1G10_CrNA**  **YTLIEESQNQQEKNEQELLELDQWASLWNWFSITNWLWYIKIFIMIVGGLVGLRIVFAVLSIANRVRQGYSPLSFQTHLP**

**H19999.7.2G7_CrNA**  **YTLIEESQNQQEKNEQELLELDQWASLWNWFSITNWLWYIKIFIMIVGGLVGLRIVFAVLSIANRVRQGYSPLSFQTHLP**

**H19999.7.2D5_CrNA**  **YTLIEESQNQQEKNEQELLELDQWASLWNWFSITNWLWYIKIFIMIVGGLVGLRIVFAVLSIANRVRQGYSPLSFQTHLP**

**H19999.7.1B2_CrNA**  **YTLIEESQNQQEKNEQELLELDQWANLWNWFDITNWLWYIKIFIMIVGGLVGLRIVFAVLSIVNRVRQGYSPLSFQTHLP**

**H19999.7.1D2_CrNA**  **YTLIEESQNQQEKNEQELLELDQWANLWNWFDITNWLWYIKIFIMIVGGLVGLRIVFAVLSIVNRVRQGYSPLSFQTHLP**

**H19507.18.G11_CrNA**  **YTLIEESQNQQEKNELELLELDKWASLWNWFDITKWLWYIKIFIMIVGGLVGLRIVFAVLSIVNKVRQGYSPLSFQIRLP**

**H19507.18.C11_CrNA**  **YTLIEESQNQQEKNELELLELDKWASLWNWFDITKWLWYIKIFIMIVGGLVGLRIVFAVLSIVNRVRQGYSPLSFQIRLP**

**H19507.18.A11_CrNA**  **YTLIEESQNQQEKNELELLELDKWASLWNWFDITKWLWYIKIFIMIVGGLVGLRIVFAVLFIVNRVRQGYSPLSFQIRLP**

**H19507.18.F4_CrNA**  **YTLIEESQNQQEKNELELLELDKWASLWNWFDITKWLWYIKIFIMIVGGLVGLRIIFAVLSIVNRVRQGYSPLSFQIRPP**

**H19793.13.F8_CrNA**  **YTLIEESQNQQEKNELELLELDKWASLWNWFDITKWLWYIKIFIMIVGGLVGLRIVFAVLSIVNRVRQGYSPLSLQIRPP**

**H19463.8.A11_CrNA**  **YHLIEESQNQQEKNEQELLELDKWASLWNWFDITSWLWYIKIFIMIVGGLVGLRIVFTVLSIVNRVRQGYSPLSFQTRFP**

**H19463.8.E10_CrNA**  **YHLIEESQNQQEKNEQELLELDKWASLWNWFDITSWLWYIKIFIMIVGGLVGLRIVFTVLSIVNRVRQGYSPLSFQTRFP**

**H19474.17.1G12_CrNA**  **YSLIESSQTQQEKNEQELLELDKWASLWNWFDITNWLWYIKIFIMIVGGLVGLRIVFTVLSIVNRVRQGYSPLSFQTRPP**

**H19474.17.2H8_CrNA**  **YSLIESSQTQQEKNEQELLELDKWASLWNWFDITNWLWYIKIFIMIVGGLVGLRIVFTVLSIVNRVRQGYSPLSFQTRPP**

**H18814.10.1E1_CrNA**  **YTLLEKSQNQQEKNEQELLELDKWASLWNWFDITNWLWYIKIFIMIVGGLVGLRIVFAVLNIVNRVRQGYSPLSLQTRLR**

**H18814.10.1B4_CrNA**  **YTLLEKSQNQQEKNEQELLELDKWASLWNWFDITNWLWYIKIFIMIVGGLVGLRIVFAVLNIVNRVRQGYSPLSLQTRLR**

**H18814.10.1C5_CrNA**  **YTLLEKSQNQQEKNEQELLELDKWASLWNWFDITNWLWYIKIFIMIVGGLVGLRIVFAVLNIVNRVRQGYSPLSLQTRLR**

**H18814.10.1B1_CrNA**  **YTLLEKSQNQQEKNEQELLELDKWASLWNWFDITNWLWYIKIFIMIVGGLVGLRIVFAVLNIVNRVRQGYSPLSLQTRLR**

**H18814.10.1G2_CrNA**  **YTLLEKSQNQQEKNEQELLELDKWASLWNWFDITNWLWYIKIFIMIVGGLVGLRIVFAVLNIVNRVRQGYSPLSLQTRLR**

**H11668.12.F11(A)_CrNA**  **YTLIEESQNQQEKNEQDLLALDKWASLWNWFDITKWLWYIKIFIMIVGGLIGLRIVFTVLSIVNRVRQGYSPLSFQTRLP**

**H11668.12.C3_CrNA**  **YTLIEESQNQQEKNEQDLLALDKWASLWNWFDITKWLWYIKIFIMIVGGLIGLRIVFTVLSIVNRVRQGYSPLSFQTRLP**

**H11668.12.D11_CrNA**  **YTLIEESQNQQEKNEQDLLALDKWASLWNWFDITKWLWYIKIFIMIVGGLIGLRIVFTVLSIVNRVRQGYSPLSFQTRLP**

**H11668.12.H9_CrNA**  **YTLIEESQNQQEKNEQDLLALDKWASLWNWFDITKWLWYIKIFIMIVGGLIGLRIVFTVLSIVNRVRQGYSPLSFQTRLP**

**H11668.12.E10_CrNA**  **YTLIEESQNQQEKNEQDLLALDKWASLWNWFDITKWLWYIKIFIMIVGGLIGLRIVFTVLSIVNRVRQGYSPLSFQTRLP**

**H19308.26.B1_CrNA**  **YTLIEESQNQQEKNEQELLELDKWASLWSWFSITNWLWYIKIFIMIVGGLVGLRIVFAVLSIVNRVRQGYSPLSLQTRLP**

**H19308.26.F8I_CrNA**  **YTLIEESQNQQEKNEQELLELDKWASLWSWFSITNWLWYIKIFIMIVGGLVGLRIVFAVLSVVNRVRQGYSPLSLQTRLP**

**H19308.26.D8_CrNA**  **YTLIEESQNQQEKNEQELLELDKWASLWSWFSITNWLWYIKIFIMIVGGLVGLRIVFAVLSVVNRVRQGYSPLSLQTRLP**

**H19308.26.D1_CrNA**  **YTLIEESQNQQEKNEQELLELDKWASLWSWFSITNWLWYIKIFIMIVGGLVGLRIVFAVLSVVNRVRQGYSPLSLQTRLP**

**H19308.26.E4_CrNA**  **YTLIEESQNQQEKNEQELLELDKWASLWSWFSITNWLWYIKIFIMIVGGLVGLRIVFAVLSVVNRVRQGYSPLSLQTRLP**

**H19885.31.D9_CrNA**  **YTLLEESQNQQEKNEQELLELDKWASLWNWFDITKWLWYIKIFIMIVGGLIGLRIVFTVLSIVNRVRKGYSPLSFQIRLP**

**H19885.31.G12_CrNA**  **YTLLEESQNQQEKNEQELLELDKWASLWNWFDITKWLWYIKIFIMIVGGLIGLRIVFTVLSIVNRVRKGYSPLSFQTRLP**

**H19885.31.E2_CrNA**  **YTLLEESQNQQEKNEQELLELDKWASLWNWFDITKWLWYIKIFIMIVGGLIGLRIVFTVLSIVNRVRKGYSPLSFQTRLP**

**H19885.31.G10_CrNA**  **YTLLEESQNQQEKNEQELLELDKWASLWNWFDITKWLWYIKIFIMIVGGLIGLRIVFTVLSIVNRVRKGYSPLSFQTRLP**

**H19885.31.H11_CrNA**  **YTLLEESQNQQEKNEQELLELDKWASLWNWFDITKWLWYIKIFIMIVGGLIGLRIVFTVLSIVNRVRKGYSPLSFQTRLP**

**H19885.31.A5_CrNA**  **YTLLEESQNQQEKNEQELLELDKWASLWNWFDITKWLWYIKIFIMIVGGLIGLRIVFTVLSIVNRVRKGYSPLSFQTRLP**

**H19885.31.F8_CrNA**  **YTLLEESQNQQEKNEQELLELDKWASLWNWFDITKWLWYIKIFIMIVGGLIGLRIVFTVLSIVNRVRKGYSPLSFQTRLP**

**H19885.31.H10_CrNA**  **YTLLEESQNQQEKNEQELLELDKWASLWNWFDITKWLWYIKIFIMIVGGLIGLRIVFTVLSIVNRVRKGYSPLSFQTRLP**

**H19885.31.G1_CrNA**  **YTLLEESQNQQEKNEQELLELDKWASLWNWFDITKWLWYIKIFIMIVGGLIGLRIVFTVLSIVNRVRKGYSPLSFQTRLP**

**H19885.31.C6_CrNA**  **YTLLEESQNQQEKNEQELLELDKWASLWNWFDITKWLWYIKIFIMIVGGLIGLRIVFTVLSIVNRVRKGYSPLSFQTRLP**

**H19885.31.E11_CrNA**  **YTLLEESQNQQEKNEQELLELDKWASLWNWFDITKWLWYIKIFIMIVGGLIGLRIVFTVLSIVNRVRKGYSPLSFQTRLP**

**H18969.12.9D9_CrNA**  **YTLIEESQNQQEKNEQELLELDKWANLWNWFDISNWLWYIKIFIMIVGGLVGLKIIFSVLSIVKKVRQGYSPLSFQTRLP**

**H18969.12.8E6_CrNA**  **YTLIEESQNQQEKNEQELLELDKWANLWNWFDISNWLWYIKIFIMIVGGLVGLRIIFSVLSIVKKVRQGYSPLSFQTRLP**

**H18969.12.8G8_CrNA**  **YTLIEESQNQQEKNEQELLELDKWANLWNWFDISNWLWYIKIFIMIVGGLVGLRIIFSVLSIVKKVRQGYSPLSFQTRLP**

**H18969.12.6D7_CrNA**  **YTLIEESQNQQEKNEQELLELDKWANLWNWFDISNWLWYIKIFIMIVGGLVGLRIIFSVLYIVNRVRQGYSPLSFQTRLP**

**H18969.12.8B4_CrNA**  **YTLIEESQNQQEKNEQELLELDKWANLWNWFDISNWLWYIKIFIMIVGGLVGLRIIFSVLSIVKRVRQGYSPLSFQTRLP**

**H18969.12.7D5_CrNA**  **YTLIEKSQNQQEKNEQELLELDKWANLWNWFDISNWLWYIKIFIMIVGGLVGLRIIFSVLSIVKRVRQGYSPLSFQTRLP**

**H18969.12.6C4_CrNA**  **YTLIEESQNQQEKNEQELLELDKWANLWNWFDISNWLWYIKIFIMIVGGLVGLRMIFSVLSIVKRVRQGYSPLSFQTRLP**

**H18969.12.10H3_CrNA**  **YTLIEESQNQQEKNEQELLELDKWANLWNWFDISNWLWYIKIFIMIVGGLVGLRIIFSVLYIVNRVRQGYSPLSFQTRLP**

**H19329.32.C9_Non-CrNA**  **YTLIEDSQKQQEKNEQELLELDTWASLWNWFSITNWLWYIKIFIMIVGGLVGLRIVFIVLSIVNRVRQGYSPLSFQTHLP**

**H19329.32.E6_Non-CrNA**  **YTLIEDSQKQQEKNEQELLELDTWASLWNWFSITNWLWYIKIFIMIVGGLVGLRIVFIVLSIVNRVRQGYSPLSFQTHLP**

**H19329.32.H7_Non-CrNA**  **YTLIEDSQKQQEKNEQELLELDTWASLWNWFSITNWLWYIKIFIMIVGGLVGLRIVFIVLSIVNRVRQGYSPLSFQTHLP**

**H19329.32.F1_Non-CrNA**  **YTLIEDSQKQQEKNEQELLELDTWASLWNWFSITNWLWYIKIFIMIVGGLVGLRIVFIVLSIVNRVRQGYSPLSFQTHLP**

**H19329.32.H5_Non-CrNA**  **YTLIEDSQKQQEKNEQELLELDTWASLWNWFSITNWLWYIKIFIMIVGGLVGLRIVFIVLSIVNRVRQGYSPLSFQTHLP**

**H19329.32.H9_Non-CrNA**  **YTLIEDSQKQQEKNEQELLELDTWASLWNWFSITNWLWYIKIFIMIVGGLVGLRIVFIVLSIVNRVRQGYSPLSFQTHLP**

**H19329.13.F12_Non-CrNA**  **YTLIEDSQKQQEKNEQELLELDTWASLWNWFSITNWLWYIKIFIMIVGGLVGLRIVFIVLSIVNRVRQGYSPLSFQTHLP**

**H18887.21.G2_Non-CrNA**  **YSLIEESQYQQEKNEKELLELDKWASLWNWFDITNWLWYIKIFIMIVGGLIGLRIVFTILSIVNRVRQGYSPLSFQTRLP**

**H18887.21.roD7_Non-CrNA** **YSLIEESQYQQEKNEKELLELDKWASLWNWFDITNWLWYIKIFIMIVGGLIGLRIVFTILSIVNRVRQGYSPLSFQTRLP**

**H19861.19.C10_Non-CrNA**  **YTLIEESQNQQEKNEQELLELNEWASLWNWFSISKWLWYIKIFIMIVGGLVGLRIVFAVLSIVNRVRQGYSPLSFQTRFP**

**H19861.19.F2_Non-CrNA**  **YTLIEESQNQQEKNEQELLELNEWASLWNWFSISKWLWYIKIFIMIVGGLVGLRIVFAVLSIVNRVRQGYSPLSFQTRFP**

**H19861.19.A6_Non-CrNA**  **YTLIEESQNQQEKNEQELLELNEWASLWNWFSISKWLWYIKIFIMIVGGLVGLRIVFAVLSIVNRVRQGYSPLSFQTRFP**

**H19489.8.G5_Non-CrNA**  **YNLLEESQNQQEKNEQELLELDKWASLWNWFDITNWLWYIRIFIMIVGGLIGLRIVFAVLSIVNRVRQGYSPLSFQTRLP**

**H19489.8.1E10_Non-CrNA**  **YNLLEESQNQQEKNEQELLELDKWASLWNWFDITNWLWYIRIFIMIVGGLIGLRIVFAVLSIVNRVRQGYSPLSFQTRLP**

**H19489.8.1A11_Non-CrNA**  **YNLLEESQNQQEKNEQELLELDKWASLWNWFDITNWLWYIRIFIMIVGGLIGLRIVFAVLSIVNRVRQGYSPLSFQTRLP**

**H19489.8.1H10_Non-CrNA**  **YNLLEESQNQQEKNEQELLELDKWASLWNWFDITNWLWYIRIFIMIVGGLIGLRIVFAVVSIVNRVRQGYSPLSFQTRLP**

**H19489.8.2A3_Non-CrNA**  **YNLLEESQNQQEKNEQELLELDKWASLWNWFDITNWLWYIRIFIMIVGGLIGLRIVFAVLSIVNRVRQGYSPLSFQTRLP**

**H19974.11.E12_Non-CrNA**  **YNLIEESQNQQEKNEQELLELDKWASLWNWFNITNWLWYIKIFIMIVGGLVGLRIVFAVLSIVNRVRKGYSPLSFQTLLP**

**H19974.11.E11_Non-CrNA**  **YNLIEESQNQQEKNEQELLELDKWASLWNWFNITNWLWYIKIFIMIVGGLVGLRIVFAVLSIVNRVRKGYSPLSFQTLLP**

**H19792.9.F6_Non-CrNA**  **YTLIEESQNQQEKNEQELLELDKWASLWNWFDITKWLWYIKIFIMIVGGLVGLRIVFAVLSIVNRVRQGYSPLSLQTRLP**

**H19792.9.B1_Non-CrNA**  **YTLIEESQNQQEKNEQELLELDKWASLWNWFDITKWLWYIKIFIMIVGGLVGLRIVFAVLSIVNRVRQGYSPLSLQTRLP**

**H19792.9.F1_Non-CrNA**  **YTLIEESQNQQEKNEQELLELDKWASLWNWFDITKWLWYIKIFIMIVGGLVGLRIVFAVLSIVNRVRQGYSPLSLQTRLP**

**H19792.9.D6_Non-CrNA**  **YTLIEESQNQQEKNEQELLELDKWASLWNWFDITKWLWYIKIFIMIVGGLVGLRIVFAVLSIVNRVRQGYSPLSLQTRLP**

**H19792.9.C10_Non-CrNA**  **YTLIEESQNQQEKNEQELLELDKWASLWNWFDITKWLWYIKIFIMIVGGLVGLRIVFAVLSIVNRVRQGYSPLSLQTRLP**

**H18880.10.20_Non-CrNA**  **YTLIEESQNQQEKNEQELLELDKWASLWNWFDITNWLWYIKIFIMIVGGLVGLRIVFAILSLVNRVRQGYSPLSFQTLLP**

**H18880.10.21_Non-CrNA**  **YTLIEESQNQQEKNEQELLELDKWASLWNWFDITNWLWYIKIFIMIVGGLVGLRIVFAILSLVNRVRQGYSPLSFQTLLP**

**H19961.14.F10_Non-CrNA**  **YTLIEESQNQQEKNEQELLELDKWASLWNWFSITKWLWYIKIFIMIVGGLVGLRIVFAVLTIVNRVRQGYSPLSFQTHRP**

**H19961.14.E8_Non-CrNA**  **YTLIEESQNQQEKNEQELLELDKWASLWNWFSITKWLWYIKIFIMIVGGLVGLRIVFAVLSIVNRVRQGYSPLSFQTHRP**

**H19961.14.G4_Non-CrNA**  **YTLIEESQNQQEKNEQELLELDKWASLWNWFSITKWLWYIKIFIMIVGGLVGLRIVFAVLSIVNRVRQGYSPLSFQTHRP**

**H19961.14.B10_Non-CrNA**  **YTLIEESQNQQEKNEQELLELDKWASLWNWFSITKWLWYIKIFIMIVGGLVGLRIVFAVLSIVNRVRQGYSPLSFQTHRP**

**H19961.14.F9_Non-CrNA**  **YTLIEESQNQQEKNEQELLELDKWASLWNWFSITKWLWYIKIFIMIVGGLVGLRIVFAVLSIVNRVRQGYSPLSFQTHRP**

**H19576.9.H1_Non-CrNA**  **YTLIEKSQNQQEKNEQELLELDKWASLWNWFDISNWLWYIKIFIMIVGGLVGLRIIFTVLAIVNRVRQGYSPLSFQTHLP**

**H19576.9.F4_Non-CrNA**  **YTLIEKSQNQQEKNEQELLELDKWASLWNWFDISNWLWYIKIFIMIVGGLVGLRIIFTVLAIMNRVRQGYSPLSFQTHLP**

810 820 830 840 850 860 870 880

....|....|....|....|....|....|....|....|....|....|....|....|....|....|....|....|

**HXB2/1-856**  **TPRGPDRPEGIEEEGGERDRDRSIRLVNGSLALIWDDLRSLCLFSYHRLRDLLLIVTRIVELLGRRGWEALKYWWNLLQY**

**H18818.6.1D2_CrNA**  **APRGPDRPDGIEEEGGEQDRGRSSRLVDGFLALIWDDLWSLCLFSYRRLRDLLLIAARVVELLGRRGWEVLKYWWNILQY**

**H18818.6.1C3_CrNA**  **APRGPDRPDGIEEEGGEQDRGRSSRLVDGFLALIWDDLWSLCLFSYRRLRDLLLIAARVVELLGRRGWEVLKYWWNILQY**

**H18818.6.1A6_CrNA**  **APRGPDRPDGIEEEGGEQDRGRSSRLVDGFLALIWDDLWSLCLFSYRRLRDLLLIAARVVELLGRRGWEVLKYWWNILQY**

**H18818.6.1G12_CrNA**  **APRGPDRPDGIEEEGGEQDRGRSSRLVDGFLALIWDDLWSLCLFSYRRLRDLLLIAARVVELLGRRGWEVLKYWWNILQY**

**H19829.11.H5_CrNA**  **APRGPDRPDGIEEEGGERDRGRSSRLVDGFLALIWDDLWSLCLFSYRRLRDLLLIAARVVELLGRRGWEVLKYWGNILQY**

**H19829.11.A2_CrNA**  **APRGPDRPDGIEEEGGERDRGRSSRLVDGFLALIWDDLWSLCLFSYRRLRDLLLIAARVVELLRRRGWEVLKYWWNILQY**

**H19829.11.E8_CrNA**  **APRGPDRPDGIEEEGGERDRGRSSRLVDGFLALIWDDLWSLCLFSYRRLRDLLLIAARVVELLGRRGWEVLKYWGNILQY**

**H19829.11.B4_CrNA**  **TPRGPDRPDGIEEEGGERDRGRSSRLVDGFLALIWDDLWSLCLFSYRRLRDLLLIAARVVELLGRRGWEVLKYWWNILQY**

**H19829.11.A4_CrNA**  **APRGPDRPDGIEEEGGERDRGRSSRLVDGFLALIWDDLWSLCLFSYRRLRDLLLIAARVVELLGRRGWEVLKYWWNILQY**

**H19829.11.G8_CrNA**  **APRGPDRPDGIEEEGGERDRGRSSRLVDGFLALIWDDLWSLCLFSYRRLRDLLLIAARVVELLGRRGWEVLKYWWNILQY**

**H19999.7.1G10_CrNA**  **AQRGPDRPEGIEEEGGERDKDRSGRLVDGFLAIIWVDLRSLCLFSYHRLRDLLLIVTRIVELLGRRGWELLKYWWNLLQY**

**H19999.7.2G7_CrNA**  **AQRGPDRPEGIEEEGGERDKDRSGRLVDGFLAIIWVDLRSLCLFSYHRLRDLLLIVTRIVELLGRRGWELLKYWWNLLQY**

**H19999.7.2D5_CrNA**  **AQRGPDRPEGIEEEGGERDKDRSGRLVDGFLAIIWVDLRSLCLFSYHRLRDLLLIVTRIVELLGRRGWELLKYWWNLLQY**

**H19999.7.1B2_CrNA**  **AQRGPDRPGGIEEEGGERDKDRSGRLVDGFLAIIWVDLRSLCLFSYHRLRDLLLIVTRIVELLGRRGWELLKYWWNLLQY**

**H19999.7.1D2_CrNA**  **AQRGPDRPEGIEEEGGERDKDRSGRLVDGFLAIIWADLRSLCLFSYHRLRDLLLIVTRIVELLGRRGWELLKYWWNLLQY**

**H19507.18.G11_CrNA**  **TPRGPDRPEGIEEEGGERDRDRSGPLVNGFLALFWEDLRSLCLFSYHRLRDLLLIVARIVELLGRRGWEALKYWWNLLQY**

**H19507.18.C11_CrNA**  **TPRGPDRPEGIEEEGGERDRDRSGPLVNGFLPLIWEDLRSLCLFSYHRLRDLLLIVARIVELLGRRGWEALKYWWNLLQY**

**H19507.18.A11_CrNA**  **TPRGPDRPEGIEEEGGERDRDRSGPLVNGFFALIWEDLRSLCLFSYHRLRDLLLIVARIVELLGRRSWEALKYWWNLLQY**

**H19507.18.F4_CrNA**  **TPRGPDRPEGIEEEGGERDRDRSGPLVNGFLALFWEDLRSLCLFSYHRLRDLLLIVARIVELLGRRGWEALKYWWNLLQY**

**H19793.13.F8_CrNA**  **VPRGPDRPEGIEEEGGDRDRDISGGSVNGFLALFWSDLRSLCLFSYHHLRDLLLIVTRIVELLGRRGWEASKYWWNLLQY**

**H19463.8.A11_CrNA**  **APRGPDRPEETEEGGGERDRDRSGPLVNGFLALFWVDLRSLFLFSYHRLRDLLLIVTRIVELLGRRGREVLKYCWNLLQY**

**H19463.8.E10_CrNA**  **APRGPDRPEETEEGGGERDRDRSSPLVNGFLALFWVDLRSLFLFSYHRLRDLLLIVTRIVELLGRRGREVLKYCWNLLQY**

**H19474.17.1G12_CrNA**  **APRGPDRPEGIEEEGGERDRDTSGPLVDGFLAIIWVDLRSLCLFSYHHLRDLLSIVTRIVGLLGRRGWEALKYWWNLLQY**

**H19474.17.2H8_CrNA**  **APRGPDRPEGIEEEGGERDRDTSGPLVDGFLAIIWVDLRSLCLFSYHHLRDLLSIVTRIVGLLGRRGWEALKYWWNLLQY**

**H18814.10.1E1_CrNA**  **APRGPDRPEGIEEEGGEQDKDRSSRLVDGFLALLWVDLRSLCLFSYHRLRDLLLIVTRIVELLGRRGWEILKYWWNLLQY**

**H18814.10.1B4_CrNA**  **APRGPDRPEGIEEEGGEQDKDRSSRLVDGFLALLWVDLRSLCLFSYHRLRDLLLIVTRIVELLGRRGWEILKYWWNLLQY**

**H18814.10.1C5_CrNA**  **APRGPDRPEGIEEEGGEQDKDRSSRLVDGFLALLWVDLRSLCLFSYHRLRDLLLIVTRIVELLGRRGWEILKYWWNLLQY**

**H18814.10.1B1_CrNA**  **APRGPDRPEGIEEEGGEQDKDRSSRLVDGFLALLWVDLRSLCLFSYHRLRDLLLIVTRIVELLGRRGWEILKYWWNLLQY**

**H18814.10.1G2_CrNA**  **APRGPDRPEGIEEEGGEQDKDRSSRLVDGFLALLWVDLRSLCLFSYHRLRDLLLIVTRIVELLGRRGWEILKYWWNLLQY**

**H11668.12.F11(A)_CrNA**  **TPREPDRPEGIEEEGGERDRDRSRSLVDGFLTLIWVDLRSLCLFSYHRLRDLLLIVTRIVELLGHRGWEILKYWWNLLQY**

**H11668.12.C3_CrNA**  **TPREPDRPEGIEEEGGERDRDRSRSLVDGLLTLIWVDLRSLCLFSYHRLRDLLLIVTRIVELLGHRGWEILKYWWNLLQY**

**H11668.12.D11_CrNA**  **TPREPDRPEGIEEEGGERDRDRSRRLVDGFLTLIWVDLWSLCLFSYHRLRDLLLIVTRIVELLGHRGWEILKYWWNLLQY**

**H11668.12.H9_CrNA**  **TPREPDRPEGIEEEGGERDRDRSRRLVDGFLTLIWVDLRSLCLFSYHRLRDLLLIVTRIVELLGHRGWEILKYWWNLLQY**

**H11668.12.E10_CrNA**  **TPREPDRPEGIEEEGGERDRDRSRRLVDGFLTLIWVDLRSLCLFSYHRLRDLLLIVTRIVELLGHRGWEILKYWWNLLQY**

**H19308.26.B1_CrNA**  **TQRGPDRPDGIEEEGGERDRGRSIRLVDGFLALIWDDLRSLCLFSYHRLRDLLLIAARIVELLGRRGWEALKYWWNLLQY**

**H19308.26.F8I_CrNA**  **TQRGPDRPDGIEEEGGERDRGRSIRLVDGFLALIWDDLRSLCLFSYHRLRDLLLIAARIVELLGRRGWEALKYWWNLLQY**

**H19308.26.D8_CrNA**  **TQRGPDRPDGIEKEGGERDRGRSVRLVDGFLALIWDDLRSLCLFSYHRLRDLLLIAARIVELLGRRGWEALKYWWNLLQY**

**H19308.26.D1_CrNA**  **TQRGPDRPDGIEEEGGERDRGRSIRLVDGFLALIWDDLRSLCLFSYHRLRDLLLIAARIVELLGRRGWEALKYWWNLLQY**

**H19308.26.E4_CrNA**  **TQRGPDRPDGIEEEGGERDRGRSIRLVDGFLALIWDDLRNLCLFSYHRLRDLLLIAARIVELLGRRGWEALKYWWNLLQY**

**H19885.31.D9_CrNA**  **AQRGPDRHGGIEEGGGERDREGSSQLVHGFLAIIWVDLRSLCLFSYHRLRDLLLIVARTVELLGRRGWEALKYWWNLLQY**

**H19885.31.G12_CrNA**  **AQRGPDRPGGIEEGGGERDREGSSQLVHGFLAIIWVDLRSLCLFSYHRLRDLLLIVARTVELLGRRGWKALKYWWNLLQY**

**H19885.31.E2_CrNA**  **AQRGPDRPGGIEEGGGERDREGSSQLVHGFLAIIWVDLRSLCLFSYHRLRDLLLIVARTVELLGRRGWEALKYWWNLLQY**

**H19885.31.G10_CrNA**  **AQRGPDRPGGIEEGGGERDREGSSQLVHGFLAIIWVDLRSLCLFSYHRLRDLLLIVARTVELLGRRGWEALKYWWNLLQY**

**H19885.31.H11_CrNA**  **AQRGPDRPGGIEEGGGERDREGSSQLVHGFLAIIWVDLRSLCLFSYHRLRDLLLIVARTVELLGRRGWEALKYWWNLLQY**

**H19885.31.A5_CrNA**  **AQRGPDRPGGIEEGGGERDREGSSQLVHGFLAIIWVDLRSLCLFSYHRLRDLLLIVARTVELLGRRGWEALKYWWNLLQY**

**H19885.31.F8_CrNA**  **AQRGPDRPGGIEEGGGERDREGSSQLVHGFLAIIWVDLRSLCLFSYHRLRDLLLIVARTVELLGRRGWEALKYWWNLLQY**

**H19885.31.H10_CrNA**  **AQRGPDRPGGIEEGGGERDREGSSQLVHGFLAIIWVDLRSLCLFSYHRLRDLLLIVARTVELLGRRGWEALKYWWNLLQY**

**H19885.31.G1_CrNA**  **AQRGPDRPGGIEEGGGERDREGSSQLVHGFLAIIWVDLRSLCLFSYHRLRDLLLIVARTVELLGRRGWEALKYWWNLLQY**

**H19885.31.C6_CrNA**  **AQRGPDRPGGIEEGGGERDREGSSQLVHGFLAIIWVDLRSLCLFSYHRLRDLLLIVARTVELLGRRGWEALKYWWNLLQY**

**H19885.31.E11_CrNA**  **AQRGPDRPGGIEEGGGERDREGSSQLVHGFLAIIWVDLRSLCLFSYHRLRDLLLIVARTVELLGRRGWEALKYWWNLLQY**

**H18969.12.9D9_CrNA**  **VPRGPDRPEGIEEEGGERDRDRSGQLVDGFLTLIWVDLRSLCLFSYHRLRDLLLIVARIVELLGRRGWEVLKYWWNLLQY**

**H18969.12.8E6_CrNA**  **VPRGPDRPEGIEEEGGERDRDRSGQLVDGFLTLIWVDLRSLCLFSYHRLRDLLLIVARIVELLGRRGWEVLKYWWNLLQY**

**H18969.12.8G8_CrNA**  **VPRGPDRPEGIEEEGGERDRDRSGQLVDGFLTLIWVDLRSLCLFSYHRLKDLLLIVARIVELLGRRGWEVLKYWWNLLQY**

**H18969.12.6D7_CrNA**  **VPRGPDRPEGIEEEGGERDRDRSGQLVDGFLTFIWVDLRSLCLFSYHRLRDLLLIVARIVELLGRRGWEVLKYWWNLLQY**

**H18969.12.8B4_CrNA**  **VPRGPDRPEGIEEEGGERDRDRSGQLVDGFLTLIWVDLRSLCLFSYHRLRDLLLIVARIVELLGRRGWEVLKYWWNLLQY**

**H18969.12.7D5_CrNA**  **VPRGPDRPEGIEEEGGERDRDRSGQLVDGFLTLIWVDLRSLCLFSYHRLRDLLLIVARIVELLGRRGWEVLKYWWNLLQY**

**H18969.12.6C4_CrNA**  **VPRGPDRPEGIEEEGGERDRDRSGQLVDGFLTLIWVDLRSLCLFSYHRLRDLLLIVARIVELLGRRGWEVLKYWWNLLQY**

**H18969.12.10H3_CrNA**  **VPRGPDRPEGIEEEGGERDRDRSGQLVDGFLTLIWVDLRSLCLFSYHRLRDLLLIVARIVELLGRRGWEVLKYWWNLLQY**

**H19329.32.C9_Non-CrNA**  **APRGPDRPEGIEEEGGERDRDRSSPLVNGFLAIIWVDLRSLCLFSYHRLRDLLLIVVRIVELLGRRGWDALKYWWNLLQY**

**H19329.32.E6_Non-CrNA**  **APRGPDRPEGIEEEGGERDRDRSGPLVNGFLAIIWVDLRSLCLFSYHRLRDLLLIVVRIVELLGRRGWEALKYWWNLLQY**

**H19329.32.H7_Non-CrNA**  **APRGPDRPEGIEEEGGERDRDRSGPLVNGFLAIIWVDLRSLCLFSYHRLRDLLLIVVRIVELLGRRGWEALKYWWNLLQY**

**H19329.32.F1_Non-CrNA**  **APRGPDRPEGIEEEGGERDRDRSGPLVNGFLAIIWVDLRSLCLFSYHRLRDLLLIVVRIVELLGRRGWEALKYWWNLLQY**

**H19329.32.H5_Non-CrNA**  **APRGPDRPEGIEEEGGERDRDRSGPLVNGFLAIIWVDLRSLCLFSYHRLRDLLLIVVRIVELLGRRGWEALKYWWNLLQY**

**H19329.32.H9_Non-CrNA**  **APRGPDRPEGIEEEGGERDRDRSGPLVNGFLAIIWVDLRSLCLFSYHRLRDLLLIVVRIVELLGRRGWEALKYWWNLLQY**

**H19329.13.F12_Non-CrNA**  **APRGPDRPEGIEEEGGERDRDRSGPLVNGFLAIIWVDLRSLCLFSYHRLRDLLLIVVRIVELLGRRGWEALKYWWNLLQY**

**H18887.21.G2_Non-CrNA**  **AQRGPDRPEGIEEEGGERDRSRSGPLVDGFLAIIWVDLRSLCLFSYRHLRDLLLILARIVGLLGRRGWEALKYWWNLLQY**

**H18887.21.roD7_Non-CrNA** **AQRGPDRPEGIEEEGGERDRSRSGPLVDGFLAIIWVDLRSLCLFSYRHLRDLLLILARIVGLLGRRGWEALKYWWNLLQY**

**H19861.19.C10_Non-CrNA**  **APRGPDGPGGIKGEGGERGRDTSGRLVNGFLELIWDDLRSLCLFSYHHLRDLLLIATRIVELLGRRGWEVLKYWWNLLQY**

**H19861.19.F2_Non-CrNA**  **APRGPDGPGGIEGEGGERGRDTSGRLVNGFLELIWDDLRSLCLFSYHHLRDLLLIATRIVELLGRRGWEVLKYWWNLLQY**

**H19861.19.A6_Non-CrNA**  **APRGPDGPGGIEGEGGERGRDTSGRLVNGFLELIWDDLRSLCLFSYHHLRDLLLIATRIVELLGRRGWEVLKYWWNLLQY**

**H19489.8.G5_Non-CrNA**  **APRGPDRPEGIEEEGGERDRDRSGILVDGFLALFWDDLRSLCLFSYHRLRDLLLIITRIVELLGRRGWEILKYWWNLLQY**

**H19489.8.1E10_Non-CrNA**  **APRGPDRPEGIEEEGGERDRDRSGILVDGFLALFWDDLRSLCLFSYHRLRDLLLIVTRIVELLGRRGWEILKYWWNLLQY**

**H19489.8.1A11_Non-CrNA**  **TPRGPDRPEGIEEEGGERDRDRSGILVDGFLALFWDDLRSLCLFSYHRLRDLLLIVTRIVELLGRRGWEILKYWWNLLQY**

**H19489.8.1H10_Non-CrNA**  **APRGPDRPEGIEEEGGERDRDRSGILVDGFLALFWDDLRSLCLFSYHRLRDLLLIVTRIVELLGRRGWEILKYWWNLLQY**

**H19489.8.2A3_Non-CrNA**  **TPRGPDRPEGIEEEGGERDRDRSGILVDGFLALFWDDLRSLCLFSYHRLRDLLLIVTRIVELLGRRGWEILKYWWNLLQY**

**H19974.11.E12_Non-CrNA**  **VPRGPDRPGGIEEEGGERDRDRSDRSVNGFLPLIWDDLRSLCLFSYHRLRDLLLIVARIVEILGRRGWEALKYWWNLLQY**

**H19974.11.E11_Non-CrNA**  **VPRGPDRPGGIEEEGGERDRDRSDRSVNGFLPLIWDDLRSLCLFSYHRLRDLLLIVARIVEILGRRGWEALKYWWNLLQY**

**H19792.9.F6_Non-CrNA**  **APRGPDRPDGIEEEGGERDRDRSGRLVNGFLALIWDDLRSLCLFSYHRLRDLLLIAARIVELLGRRGWEALKYWWNLLQY**

**H19792.9.B1_Non-CrNA**  **APRGPDRPDGIEEEGGERDRDRSGRLVNGFLALIWDDLRSLCLFSYHRLRDLLLIAARIVELLGRRGWEALKYWWNLLQY**

**H19792.9.F1_Non-CrNA**  **APRGPDRPDGIEEEGGERDRDRSGRLVNGFLALIWDDLRSLCLFSYHRLRDLLLIAARIVELLGRRGWEALKYWWNLLQY**

**H19792.9.D6_Non-CrNA**  **APRGPDRPDGIEEEGGERDRDRSGRLVNGFLALIWDDLRSLCLFSYHRLRDLLLIAARIVELLGRRGWEALKYWWNLLQY**

**H19792.9.C10_Non-CrNA**  **APRGPDRPDGIEEEGGERDRDRSGRLVNGFLALIWDDLRSLCLFSYHRLRDLLLIAARIVELLGRRGWEALKYWWNLLQY**

**H18880.10.20_Non-CrNA**  **APRGPDRPDGIEEEGGERDRDRSGRLVDGFLALIWDDLRSLCLFSYRRLRDLLLIAARIVELLGRRGWEALKYWWNLLQY**

**H18880.10.21_Non-CrNA**  **APRGPDRPDGIEEEGGERDRDRSGRLVDGFLALIWDDLRSLCLFSYRRLRDLLLIAARIVELLGRRGWEALKYWWNLLQY**

**H19961.14.F10_Non-CrNA**  **APRGPDRPEGIEEDGGERDRDRSGQLVDGLLALIWVDLRSLCLFSYHHLRDLLLIVTRIVELLGRRGWEALKYWWNLLQY**

**H19961.14.E8_Non-CrNA**  **APRGPDRPEGIEEDGGERDRDRSGQLVDGLLALIWVDLRSLCLFSYHHLRDLLLIVTRIVELLGRRGWEALKYWWNLLQY**

**H19961.14.G4_Non-CrNA**  **APRGPDRPEGIEEDGGERDRDRSGQLVDGLLALIWVDLRSLCLFSYHHLRDLLLIVTRIVELLGRRGWEALKYWWNLLQY**

**H19961.14.B10_Non-CrNA**  **APRGPDRPEGIEEDGGERDRDRSGQLVDGLLALIWVDLRSLCLFSYHHLRDLLLIVTRIVELLGRRGWEALKYWWNLLQY**

**H19961.14.F9_Non-CrNA**  **APRGPDRPEGIEEDGGERDRDRSGQLVDGLLALIWVDLRSLCLFSYHHLRDLLLIVTRIVELLGRRGWEALKYWWNLLQY**

**H19576.9.H1_Non-CrNA**  **AQRGPDRPEGIEEEGGDKDKDKSSRLVDGFLALLWVDLRSLCLFSYHRLRDLLLIATRIVELLGRRGWELLKYWWNLLKY**

**H19576.9.F4_Non-CrNA**  **APRGPDRPEGIEEEGGDRDKDKSSRLVDGFLAILWVDLRSLCLFSYHRLRDLLLIATRIVELLGRRGWELLKYWWNLLKY**

890 900 910 920 930

....|....|....|....|....|....|....|....|....|....|....

**HXB2/1-856**  **WSQELKNSAVSLLNATAIAVAEGTDRVIEVVQGACRAIRHIPRRIRQGLERILL**

**H18818.6.1D2_CrNA**  **WSQELKNSAVSLFNAIAIAVAEGTDRAIEIVQRAYRAIFHIPRRIRQGLERALL**

**H18818.6.1C3_CrNA**  **WSQELKNSAVSLFNAIAIAVAEGTDRAIEIVQRAYRAIFHIPRRIRQGLERALL**

**H18818.6.1A6_CrNA**  **WSQELKNSAVSLFNAIAIAVAEGTDRAIEIVQRAYRAIFHIPRRIRQGLERALL**

**H18818.6.1G12_CrNA**  **WSQELKNSAVSLFNAIAIAVAEGTDRAIEIVQRAYRAIFHIPRRIRQGLERALL**

**H19829.11.H5_CrNA**  **WSQELKKSAVSLFNAIAIAVAEGTDRAIEIVQRAFRAVFHIPRRIRQGLERALL**

**H19829.11.A2_CrNA**  **WGQELKKSAVSLFNAIAIAVAEGTDRAIEIVQRAFRAVFHIPRRIRQGLERALL**

**H19829.11.E8_CrNA**  **WSQELKKSAVSLFNAIAIAVAEGTDRAIEIVQRAFRAVFHIPRRIRQGLERALL**

**H19829.11.B4_CrNA**  **WSQELKKSAVSLFNAIAIAVAEGTDRAIEIVQRAFRAILHIPRRTRQGLERALL**

**H19829.11.A4_CrNA**  **WSQELKKSAISLFNAIAVAVAEGTDRAIEIVQRAYRAIIHIPRRIRQGFERALL**

**H19829.11.G8_CrNA**  **WSQELKKSAVSLFNAIAVAVAEGTDRAIEIVQRAYRAIIHIPRRTRQGLERALL**

**H19999.7.1G10_CrNA**  **WSQELKNSAVSLLNATAIAVAEGTDRIIEVSQRAFRAILHIPTRIRQGLERALL**

**H19999.7.2G7_CrNA**  **WSQELKNSAVSLLNATAIAVAEGTDRIIEVSQRAFRAILHIPTRIRQGLERALL**

**H19999.7.2D5_CrNA**  **WSQELKNSAVSLLNATAIAVAEGTDRIIEVSQRAFRAILHIPTRIRQGLERALL**

**H19999.7.1B2_CrNA**  **WSQEIKNSAVSLLNATAIAVAEGTDRIIEVSQRAFRAILHIPTRIRQGLERALL**

**H19999.7.1D2_CrNA**  **WSQELKNSAVSLLNATAIAVAEGTDRIIEVSQRAFRAILHIPTRIRQGLERALL**

**H19507.18.G11_CrNA**  **WSQELRNSAVSLLDAIAIAVAEGTDRIIETVQRIYRAILNIPTRIRQGLELALL**

**H19507.18.C11_CrNA**  **WSQEIKNSAVSLLNATAITVAEGTDRIIEIVQRIYRAILNIPTRIRQGLELALL**

**H19507.18.A11_CrNA**  **WIQELKNSAVSLLNATAIAVAEGTDRIIDIVQRIYRAILNIPTRIRQGLELALL**

**H19507.18.F4_CrNA**  **WSQELRNSAVSLLNATAIAVAEGTDRIIEIAQRIYRAILNIPTRIRQGLELALL**

**H19793.13.F8_CrNA**  **WSQELKNSAVSLFNATAIAVAEGTDRVIEVLQRAYRAILHIPRRIRQGFERALL**

**H19463.8.A11_CrNA**  **WSQELKNSAVSLLNATAIVVAEGTDRVIEVVQRACRAILHIPTRIRQGFERALL**

**H19463.8.E10_CrNA**  **WSQELKNSAVSLLNATAIVVAEGTDRVIEVVQRACRAILHIPTRIRQGFERALL**

**H19474.17.1G12_CrNA**  **WSQELKNSAVSLFNAIAIAVAEGTDRVIEVLQRIGRAFLHIPTRIRQGLERALL**

**H19474.17.2H8_CrNA**  **WSQELKNSAVSLFNAIAIAVAEGTDRVIEVLQRIGRAFLHIPTRIRQGLERALI**

**H18814.10.1E1_CrNA**  **WSQELKNSAVSLLNATAIAVAEGTDRVIEVLQRAFRAILHIPTRIRQGFERALL**

**H18814.10.1B4_CrNA**  **WSQELKNSAVSLLNATAIAVAEGTDRVIEVLQRAFRAILHIPTRIRQGFERALL**

**H18814.10.1C5_CrNA**  **WSQELKNSAVSLLNATAIAVAEGTDRVIEVLQRAFRAILHIPTRIRQGFERALL**

**H18814.10.1B1_CrNA**  **WSQELKNSAVSLLNATAIAVAEGTDRVIEVLQRAFRAILHIPTRIRQGFERALL**

**H18814.10.1G2_CrNA**  **WSQELKNSAVSLLNATAIAVAEGTDRVIEVLQRAFRAILHIPTRIRQGFERALL**

**H11668.12.F11(A)_CrNA**  **WSQELKNSAVSLLNATAIAVAEGTDRIIEGVQRAYRAVLHVPRRIRQGFERALL**

**H11668.12.C3_CrNA**  **WSQELKNSAVSLLNATAIAVAEGTDRIIEVIQRAYRAILHVPRRIRQGFERALL**

**H11668.12.D11_CrNA**  **WSQELKNSAVSLLNATAIAVAEGTDRIIEVIQRAYRAVLHVPRRIRQGFERALL**

**H11668.12.H9_CrNA**  **WSQELKNSAVSLLNATAIAVAEGTDRIIEVVRRACRAVLHVPRRIRQGFERALL**

**H11668.12.E10_CrNA**  **WSQELKNSAVSLLNATAIAVAEGTDRIIEVVRRAYRAVLHVPRRIRQGFERALL**

**H19308.26.B1_CrNA**  **WSQELKNSAINLLNATAIAVAEGTDRVIEVVQRICRAILHIPIRIRQGLERLLL**

**H19308.26.F8I_CrNA**  **WSQELKNSAINLLNATAIAVAEGTDRVIEVVQRICRAILHIPIRIRQGLERLLL**

**H19308.26.D8_CrNA**  **WSQELKNSAINLLNATAIAVAEGTDRVIEVVQRICRAILHIPTRIRQGSERLLL**

**H19308.26.D1_CrNA**  **WSQELKNSASSLLNATAIAVAEGTDRVIEVVQRICRAILHIPTRIRQGFERLLL**

**H19308.26.E4_CrNA**  **WSQELKNSATSLLNATAIAVAEGTDRVIEVVQRICRAILHIPTRIRQGFERLLL**

**H19885.31.D9_CrNA**  **WSQELKKSAVSLLNAIAIVVAEGTDRVIEVVQRTCRAIRNIPTRIRQGLERVLL**

**H19885.31.G12_CrNA**  **WSQELKKSAVSLLNAIAIVVAEGTDRVIEVVQRTCRAIRNIPTRIRQGLERALL**

**H19885.31.E2_CrNA**  **WSQELKKSAVSLLNAIAIVVAEGTDRVIEVVQRTCRAIRNIPTRIRQGLERALL**

**H19885.31.G10_CrNA**  **WSQELKKSAVSLLNAIAIVVAEGTDRVIEVVQRTCRAIRNIPTRIRQGSERALL**

**H19885.31.H11_CrNA**  **WSQELKKSAVSLLNAIAIVVAEGTDRVIEVVQRTCRAIRNIPTRIRQGLERALL**

**H19885.31.A5_CrNA**  **WSQELKKSAVSLLNAIAIVVAEGTDRVIEVVQRTCRAIRNIPTRIRQGLERTLL**

**H19885.31.F8_CrNA**  **WSQELKKSAVSLLNAIAIVVAEGTDRVIEVVQRTCRAIRNIPTRIRQGLERALL**

**H19885.31.H10_CrNA**  **WSQELKKSAVSLLNAIAIVVAEGTDRVIEVVQRTCRAIRNIPTRIRQGLERALL**

**H19885.31.G1_CrNA**  **WSQELKKSAVSLLNAIAIVVAEGTDRVIEVVQRTCRAIRNIPTRIRQGLERALL**

**H19885.31.C6_CrNA**  **WSQELKKSAVSLLNAIAIVVAEGTDRVIEVVQRTCRAIRNIPTRIRQGLERALL**

**H19885.31.E11_CrNA**  **WSQELKKSAVSLLNAIAIVVAEGTDRVIEVVQRTCRAIRNIPTRIRQGLERALL**

**H18969.12.9D9_CrNA**  **WSQELKNSAVSLYNTTAIAVAEGTDRVIEVLQRAYRAFLHIPRRIRQGAERALQ**

**H18969.12.8E6_CrNA**  **WSQELKNSAVSLYNTTAIAVAEGTDRVTEVLQRAYRAFLHIPRRIRQGAERALQ**

**H18969.12.8G8_CrNA**  **WSQELKNSAVSLYNTTAIAVAEGTDRIIEVLQRAYRAFLHIPRRIRQGAERALQ**

**H18969.12.6D7_CrNA**  **WSQELKNSAASLYNTTAIAVAEGTDRVIEVLQRAYRAFLHIPRRIRQGAERALQ**

**H18969.12.8B4_CrNA**  **WSQELKNSAVSLYNTTAIAVAEGTDRVLEVLQRAYRAFLHIPRRIRQGAERALQ**

**H18969.12.7D5_CrNA**  **WSQELKNSAVSLYNTTAIAVAEGTDRIIEVLQRAYRAFLHIPRRIRQGAERALQ**

**H18969.12.6C4_CrNA**  **WSQELKNSAVSLYNTTAIAVAEGTDRAIEVLQRAYRAFLHIPRRIRQGAERALQ**

**H18969.12.10H3_CrNA**  **WSQELKNSAVSLYNTTAIAVAEGTDRIIEVLQRAYRAFLHIPRRIRQGAERALQ**

**H19329.32.C9_Non-CrNA**  **WSQELRSSAVSLFNAIAIAVAEGTDRVIETIQRTFRAILHIPRRIRQGLERLLL**

**H19329.32.E6_Non-CrNA**  **WIQELRGSAVSLFNAIAIAVAEGTDRVIETIQRTFRAILHIPRRIRQGLERLLL**

**H19329.32.H7_Non-CrNA**  **WIQELRGSAVSLFNAIAIAVAEGTDRVIETIQRTFRAILHIPRRIRQGLERLLL**

**H19329.32.F1_Non-CrNA**  **WIQELRGSAVSLFNAIAIAVAEGTDRVIETIQRTFRAILHIPRRIRQGLERLLL**

**H19329.32.H5_Non-CrNA**  **WIQELRGSAVSLFNAIAIAVAEGTDRVIETIQRTFRAILHIPRRIRQGLERLLL**

**H19329.32.H9_Non-CrNA**  **WIQELRGSAVSLFNAIAIAVAEGTDRVIETIQRTFRAILHIPRRIRQGLERLLL**

**H19329.13.F12_Non-CrNA**  **WIQELRGSAVSLFNAIAIAVAEGTDRVIETIQRTFRAILHIPRRIRQGLERLLL**

**H18887.21.G2_Non-CrNA**  **WSQELKNSAVSLLNATAIVVAEGTDRVIEVVQRACRAILNIPRRIRQGAERALI**

**H18887.21.roD7_Non-CrNA** **WSQELKNSAVSLLNATAIVVAEGTDRVIEVVQRACRAILNIPRRIRQGAERALI**

**H19861.19.C10_Non-CrNA**  **WSQELKNSAVSLLNATAIAVAEGTDRIIEVVQRAGRAILHIPTRIRQGLERTLL**

**H19861.19.F2_Non-CrNA**  **WSQELKNSAVSLLNATAIAVAEGTDRIIEVVQRAGRAILHIPTRIRQGLERTLL**

**H19861.19.A6_Non-CrNA**  **WSQELKNSAVSLLNATAIAVAEGTDRIIEVVQRAGRAILHIPTRIRQGLERALL**

**H19489.8.G5_Non-CrNA**  **WSQELKNSAVSLLNVTAIAVAEGTDRIIELIQRAYRAVLHVPRRIRQGFERALL**

**H19489.8.1E10_Non-CrNA**  **WSQELKNSAVSLLNVTAIAVAEGTDRIIELIQRAYRAVLHVPRRIRQGFERALL**

**H19489.8.1A11_Non-CrNA**  **WSQELKNSAVSLLNVTAIAVAEGTDRIIELIQRAYRAVLHVPRRIRQGFERALL**

**H19489.8.1H10_Non-CrNA**  **WSQELKNSAVSLLNVTAIAVAEGTDRIIELIQRAYRAVLHVPRRIRQGFERALL**

**H19489.8.2A3_Non-CrNA**  **WSQELKNSAVSLLNVTAIAVAEGTDRIIELIQRAYRAVLHVPRRIRQGFERALL**

**H19974.11.E12_Non-CrNA**  **WIQELKNSAVSLLNATAIAVAEGTDRIIEVVQRACIAILHIPRRVRQGLERALQ**

**H19974.11.E11_Non-CrNA**  **WIQELKNSAVSLLNATAIAVAEGTDRIIEVVQRAWRAILHIPRRVRQGLERALQ**

**H19792.9.F6_Non-CrNA**  **WIQELKNSAVSLLNATAIAVAEGTDRVIEVVQRTCRAIRHIPRRVRQGLERSLL**

**H19792.9.B1_Non-CrNA**  **WIQELKNSAVSLLNATAIAVAEGTDRVIEVVQRTCRAIRHIPRRIRQGLERSLL**

**H19792.9.F1_Non-CrNA**  **WIQELKNSAVSLLNATAIAVAEGTDRVIEVVQRTCRAIRHIPRRIRQGFERSLL**

**H19792.9.D6_Non-CrNA**  **WIQELKNSAVSLLNATAIAVAEGTDRVIEVVQRTCRAIRHIPRRIRQGFERSLL**

**H19792.9.C10_Non-CrNA**  **WIQELKNSAVSLLNATAIAVAEGTDRVIEVVQRTCRAIRHIPRRIRQGFERSLL**

**H18880.10.20_Non-CrNA**  **WSQELKNSAVSLLNATAIAVAEGTDRVIEIVQRAFRAILHIPVRIRQGLERALL**

**H18880.10.21_Non-CrNA**  **WSQELKNSAVSLLNATAIAVAEGTDRVIEVVQRAFRAVLHIPVRIRQGLERALL**

**H19961.14.F10_Non-CrNA**  **WGQELKKSAVSLLNATAIAVAEGTDRVVEILQRAGRAILNIPRRIRQGFERALQ**

**H19961.14.E8_Non-CrNA**  **WGQELKKSAVSLLNATAIAVAEGTDRVVEILQRAGRAILNIPRRIRQGFERALQ**

**H19961.14.G4_Non-CrNA**  **WGQELKKSAVSLLNATAIAVAEGTDRVVEILQRAGRAILNIPRRIRQGFERALQ**

**H19961.14.B10_Non-CrNA**  **WGQELKKSAVSLLNATAIAVAEGTDRVVEILQRAGRAILNIPRRIRQGFERALQ**

**H19961.14.F9_Non-CrNA**  **WGQELKKSAVSLLNATAIAVAEGTDRVVEILQRAGRAILNIPRRIRQGFERALQ**

**H19576.9.H1_Non-CrNA**  **WSQELKNSAVSLLNATAIAVAEGTDRIIEVSQRAFRAILNVPRRIRQGFERVLL**

**H19576.9.F4_Non-CrNA**  **WSQELKNSAVSLLNATAIAVAEGTDRIIEVSQRAFRAILNVPRRIRQGFERALL**
